# Identifying long non-coding RNAs and characterizing their functional roles in swine mammary gland from colostrogenesis to lactogenesis

**DOI:** 10.5713/ab.21.0308

**Published:** 2021-10-29

**Authors:** Lijun Shi, Longchao Zhang, Ligang Wang, Xin Liu, Hongmei Gao, Xinhua Hou, Fuping Zhao, Hua Yan, Wentao Cai, Lixian Wang

**Affiliations:** 1 Institute of Animal Science, Chinese Academy of Agricultural Sciences, Beijing 100193, China

**Keywords:** Colostrum, LncRNA, Mammary Gland, Pig, Weighted Gene Co-expression Network Analysis (WGCNA)

## Abstract

**Objective:**

This study was conducted to identify the functional long non-coding RNAs (lncRNAs) for swine lactation by RNA-seq data of mammary gland.

**Methods:**

According to the RNA-seq data of swine mammary gland, we screened lncRNAs, performed differential expression analysis, and confirmed the functional lncRNAs for swine lactation by validation of genome wide association study (GWAS) signals, functional annotation and weighted gene co-expression network analysis (WGCNA).

**Results:**

We totally identified 286 differentially expressed (DE) lncRNAs in mammary gland at different stages from 14 days prior to (-) parturition to day 1 after (+) parturition, and the expressions of most of lncRNAs were strongly changed from day −2 to day +1. Further, the GWAS signals of sow milk ability trait were significantly enriched in DE lncRNAs. Functional annotation revealed that these DE lncRNAs were mainly involved in mammary gland and lactation developing, milk composition metabolism and colostrum function. By performing weighted WGCNA, we identified 7 out of 12 lncRNA-mRNA modules that were highly associated with the mammary gland at day −14, day −2, and day +1, in which, 35 lncRNAs and 319 mRNAs were involved.

**Conclusion:**

This study suggested that 18 lncRNAs and their 20 target genes were promising candidates for swine parturition and colostrum occurrence processes. Our research provided new insights into lncRNA profiles and their regulating mechanisms from colostrogenesis to lactogenesis in swine.

## INTRODUCTION

Although litter size of sow has made a significant improvement during last two decades, the problem of growth performance and mortality in piglets has become prominent. During gestation and lactation, the growth performance and health status of piglets have been critical factors impacting reproductive performance of modern sows [1]. Sow milk performance is a major limiting factor and contributes to suboptimal growth and survival of piglets [2]. The quality and quantity of milk in sows are highly variable, for example, the colostrum yield is reported to range from <1 kg to 8.5 kg [3,4]. To enhance the sow lactation ability, it is necessary to understand the mammary gland development, and screen their regulatory factors [5]. In 2016, Balzani et al [6] reported the heritability of udder morphology in crossbreed sows (Large White×Meishan), namely, *h*^2^: 0.1 to 0.56. Generally, from colostrogenesis to lactogenesis, mammary glands might undergo significant functional differentiation for swine parturition and colostrum occurrence processes. In 2018, Palombo et al [7] used the RNA sequencing (RNA-seq) of mammary gland to detect the candidate genes impacting swine parturition and lactation in crossbred sows (Danish Landrace×Yorkshire).

Long non-coding RNA (lncRNA) is a recently identified class of non-protein coding transcripts in eukaryotes with a minimum length of 200 nt [8], and it plays an important regulatory role in biological processes [9]. Many lncRNAs have been reported along with the depth and quality of RNA-seq [10]. In swine, many potential regulatory lncRNAs have been identified from various tissues, such as intramuscular adipose [11,12], longissimus dorsi muscle [13], preadipocytes [14], and porcine endometrium [15]. In 2018, Liang et al [9] built a systematic *Sus scrofa* lncRNA database named lncRNAnet that contained 53,468 *S. scrofa* lncRNAs with their sequence characteristics, genomic locations, conservation, overlapping single nucleotide polymorphism (SNP) and quantitative trait loci, and transcript abundance across nine tissues (fat, heart, kidney, liver, lung, muscle, ovarium, spleen, and testis). However, the identification of lncRNAs regulating milk traits from sow mammary gland is still lacking.

In present study, we identified lncRNAs of sow mammary gland at different stages from 14 days prior to parturition to day 1 after parturition according to the published RNA-seq data, and detected the differentially expressed (DE) lncRNAs. Further, validation of genome wide association study (GWAS) signals, functional annotation and weighted gene co-expression network analysis (WGCNA) were conducted to predict the regulatory functions of DE lncRNAs. Our results will pave the way for a better understanding of lncRNA functions in swine parturition and colostrum occurrence processes.

## MATERIALS AND METHODS

### Ethic statement

The data (BioProject ID: GSE101983) was in the Gene Expression Omnibus database.

### RNA-seq dataset

The RNA-seq data sources (GSE101983) were involved in 15 mammary gland tissues, which were collected from 3 crossbred sows (Danish Landrace×Yorkshire) at days 14, 10, 6, and 2 before (−) parturition and day 1 after (+) parturition (Supplementary Table S1)[7].

### Identification of lncRNAs

Clean reads were obtained by removing adapter molecules, reads including poly-N, and low-quality reads using Trimmomatic (0.39) with defaults parameters [16]. All follow-up analyses were performed with the high-quality reads. Through STAR software (2.7.3a), we aligned clean reads to the pig reference genome (Sscrofa11.1).

**Figure 1:**
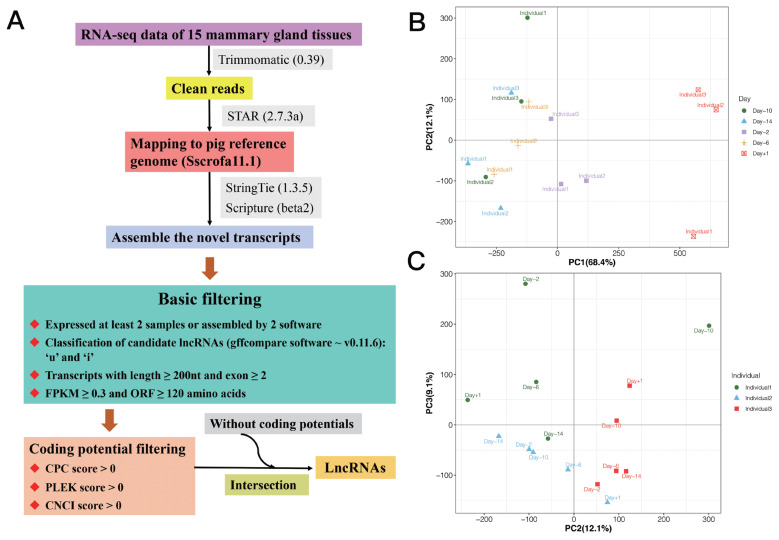
(A) The process for identifying lncRNAs. Principal component analysis (PCA) analysis based on the expression of lncRNAs: (B) PC1 and PC2 could separate the samples by phenotype of stages of lactation; and (C) PC2 and PC3 could separate the samples by individuals.

We used StringTie (1.3.5) and Scripture (beta2) to assemble the novel transcripts. Transcripts occurred in at least two samples or supported by two assembly software were obtained [17,18]. Further, the putative novel lncRNAs were detected via a customized multi-step pipeline: i) The transcripts were removed, which were likely to be assembly artifacts or polymerase chain reaction run-on fragments based on class code annotated by gffcompare (v0.11.6). For the different class, we retained those only annotated by ‘u’ and ‘i’, which indicated novel intergenic and intronic transcripts, respectively. Transcripts with class code “=” were considered as known genes. ii) Transcripts with length ≥200 nt and exon ≥2 were retained to avoid incomplete assemble and too many splicing events. iii) Transcripts with low expression were removed using fragments per kilobase of exon model per million mapped fragments (FPKM) ≥0.3 as cut off. The expression levels of transcripts were calculated in fragments per kilo-base of exon per 10^6^ mapped fragments (FPKM) by StringTie −e −B. iv) Maximum open reading frame lengths of less than 120 amino acids (360 nt) were obtained. v) Transcripts with predicted protein-coding potential were removed, that the protein-coding potential criteria were CPC score >0, PLEK score >0, and CNCI score >0. Finally, the candidate sets of lncRNAs were made up by those without coding potentials. The process for identifying lncRNAs is shown in Figure 1A.

### Differential expression analysis of lncRNAs

To identify the longitudinal transcriptional lncRNAs response close to parturition, and to highlight the metabolic processes underlying mammary changes associated with the colostrogenesis and lactogenesis in the last stages of gestation leading up to parturition, we used the time point −14 day as baseline to detect DE lncRNAs and coding genes with EdegR R package. RNA-Seq read counts were modeled by a generalized linear model that considered the experimental design with two factors (individuals and stages of lactation). The formula in EdegR is shown as follow: Design = ~Individuals+Stages. LncRNAs or genes with adjusted p-value<0.05 were assigned as the differences. Further, we performed expression patterns of DE lncRNAs across five stages by k-mean method [19].

### Validation of genome wide association study signals

We collected milk ability phenotypes of 985 sows from Shanxi and Liaoning Province of China in 2019 through 2020. Their genotypes were measuring using GenSeek Genomic Profiler Porcine 50K (50,697 SNPs), Illumina, San Diego, CA, USA), which contained 50,697 SNPs. The milk ability was calculated by the following formula: litter weight at weaning – litter weight at born – litter weight at the time of fostering in + litter weight at death + litter weight at the time of fostering out. We estimated the breeding value of milk produce ability with ASReml package, in which, fixed effects of herd by farm and production batch (9 levels), parity (5 levels: 1, 2, 3, 4, and 5–8), and days of lactation (3 levels: ≤18, 19 to 21, and >21) were considered. We conducted the quality control for genotyping data by removing the SNPs with minor allele frequencies <0.01, and a deviation from Hardy-Weinburg equilibrium p values <0.001. Finally, 36,871 SNPs and 985 phenotypic breeding values were used for GWAS by the Fixed and random effect model Circulating Probability Unification (FarmCPU). The genotype data used for GWAS and effects of SNPs were submitted to public repositories, and DOI was 10.6084/m9. figshare.16455963 (https://figshare.com/s/2112a49f046dfda5d41f).

For investigating the association of DE lncRNAs with GWAS signals, we performed enrichment analysis of SNPs from GWAS for sow milk ability trait (GitHub: https://github.com/WentaoCai/GWAS_enrichment) [20]. We separately conducted GWAS signals enrichment analysis based on 1,084 identified lncRNAs and 286 DE lncRNAs regions using a sum-based method. The formula was the following:


$$T_{\mathrm{sum}}=\sum_{i=1}^{m_{g}}\beta^{2}$$

Where, ***T****_sum_* was the summary statistics for a tested lncRNA group, ***β*** was the marker effect of milk ability estimated by the GWAS statistics, and ***m****_g_* was the number of SNPs located in the tested lncRNAs or 10 Kb up-/down-stream of the tested lncRNAs, in which, there were 1,571 and 485 SNPs for 1,084 and 286 lncRNAs, respectively. We repeated the permutation for 10,000 times in each studied genomic feature and calculated an empirical p-value according to one-tailed test of the proportion of randomly sampled summary statistics larger than that observed.

### Target gene prediction of lncRNAs and functional analysis

LncRNAs can cis-regulate neighboring target genes and trans-regulate distant target genes [21,22]. To predict cis-regulated target genes of lncRNAs, the coding genes located within 100 kb upstream/downstream of lncRNAs were checked. Further, we computed the expressed correlation coefficients between lncRNAs and their neighboring genes by Spearman method, in which, the significant lncRNA-mRNA correlation pairs with p-value <0.05 were assigned.

To investigate the functions of DE lncRNAs, we performed gene ontology (GO) and Kyoto encyclopedia of genes and genomes (KEGG) enrichments using KOBAS (http://kobas.cbi.pku.edu.cn/kobas3/genelist/) [23]. The GO terms and pathways with p-value <0.05 were considered significant.

### Module construction base on WGCNA

WGCNA is a systematic in silico method for analysis of complex gene regulatory networks, which can be used for finding modules of significantly correlated genes, and for relating modules to one another and to external sample traits [24,25]. Here, we conducted WGCNA to detect significant modules by WGCNA R package. In significant modules, we screened the lncRNAs and genes, which might be candidates to impact the swine lactation developing, milk fat and protein metabolism, and colostrum function.

## RESULTS

### Identification of lncRNAs

In this study, RNA-seq data of 15 samples from mammary gland of three crossbred sows with five time points were used (Supplementary Table S1) [7]. After quality control, reads mapping and lncRNA identification, a total of 1,346 lncRNA transcripts located in 1,084 lncRNA loci were detected (Supplementary Table S2), in which, 483 (613 lncRNA transcripts) were novel lncRNAs, and 601 (733 lncRNA transcripts) were known lncRNAs. Additionally, we used the expression of lncRNAs to perform principal component analysis (PCA) analysis, and the result is shown in Figure 1B–C, in which, PC1 and PC2 could separate the samples by phenotype of stages of lactation, and PC2 and PC3 could separate the samples by individuals.

### Differential expression analysis

**Figure 2:**
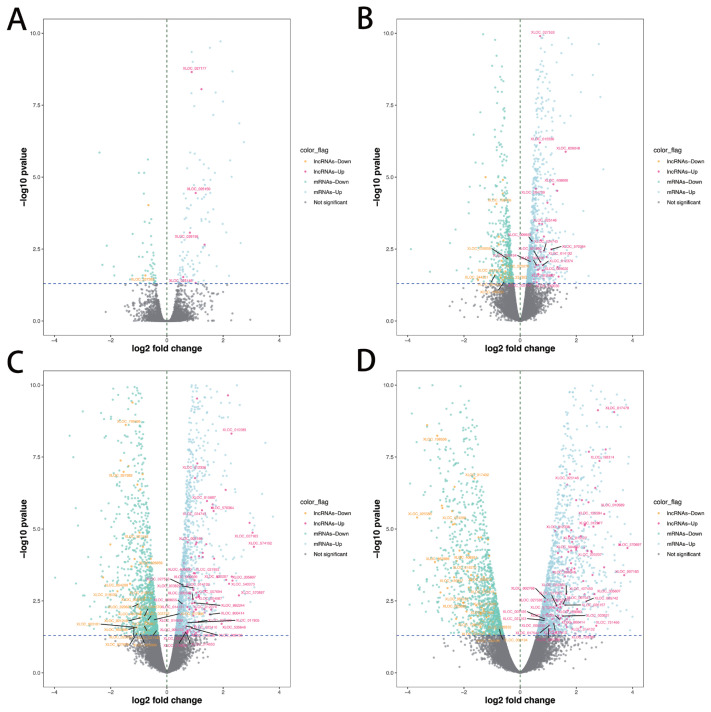
Identification of DE lncRNAs and mRNAs. (A) Volcano plot dispalys DE lncRNAs and mRNAs of day −10 vs day −14. (B) Volcano plot dispalys DE lncRNAs and mRNAs of day −6 vs day −14. (C) Volcano plot dispalys DE lncRNAs and mRNAs of day −2 vs day −14. (D) Volcano plot dispalys DE lncRNAs and mRNAs of day +1 vs day −14. The novel DE lncRNAs was marked. DE, differentially expressed.

**Figure 3:**
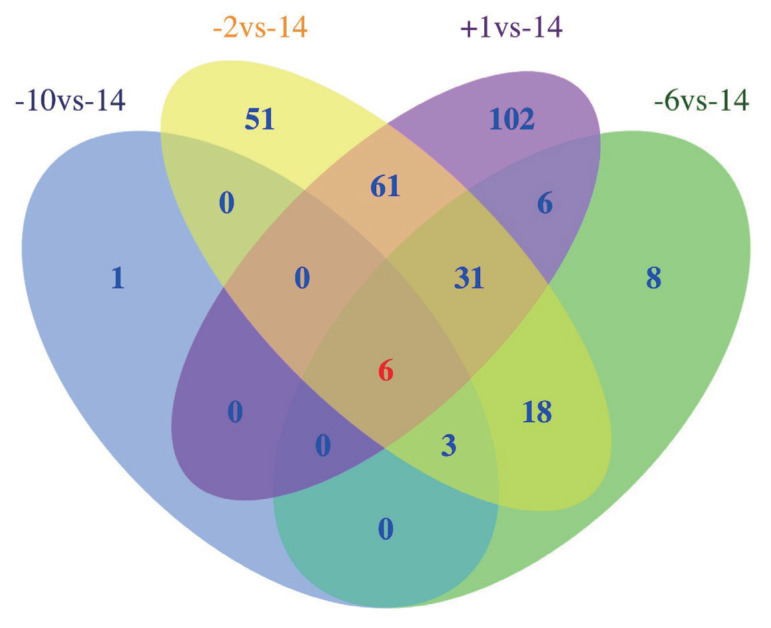
Venn diagram shows the number of differentially expressed (DE) lncRNAs overlapping in different swine mammary gland development stages or unique to each developmental stage. −14, at days 14 before parturition; −10, at days 10 before parturition; −6, at days 6 before parturition; −2, at days 2 before parturition; +1, at days 1 after parturition.

**Figure 4:**
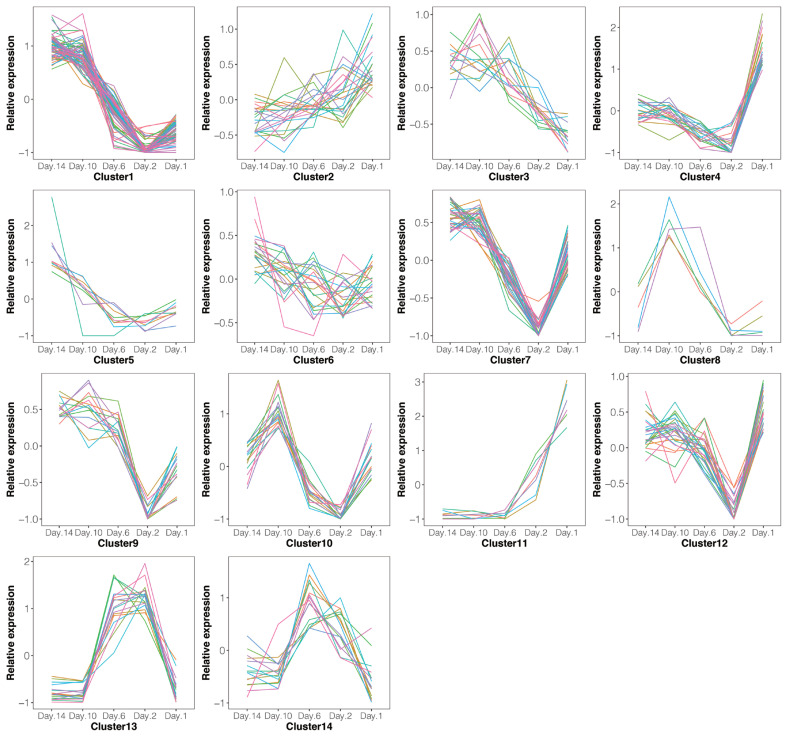
Expression pattern of differentially expressed (DE) lncRNAs across five stages. The colored lines represent that the different lncRNA changes.

A total of 286 lncRNAs (Supplementary Table S3) were DE (adjusted p-value<0.05), including 9 (7 up-regulated and 2 down-regulated), 71 (41 up-regulated and 30 down-regulated), 169 (101 up-regulated and 68 down-regulated) and 206 (128 up-regulated and 78 down-regulated) for −10 vs −14, −6 vs −14, −2 vs −14, and 1 vs −14 comparison groups, respectively (Figure 2A–D). Notably, six lncRNAs were DE in all four comparison groups (Figure 3). Further, we performed the gap statistics analysis for 286 DE lncRNAs, and found that k = 14 was the optimal choice for cluster analysis. The result of gap statistics analysis is shown in Supplementary Figure S1. We examined the expression pattern of DE lncRNAs across five lactation stage using k-means clustering, and these lncRNAs could be divided into 14 distinct clusters. In cluster 4, 7, 9, 10, and 12, most of DE lncRNAs expressions were rapidly decreased at day −6 and day −2, and strongly increased at day +1, which indicated these lncRNAs were dynamic and changed from colostrogenesis to lactogenesis (Figure 4).

### Enrichment analysis of GWAS signals

To assess whether mammary lncNRAs identified in this study were associated with GWAS signals, we performed enrichment analysis of sow milk ability trait for 1,084 detected lncRNAs and 286 DE lncRNAs, separately. For comparison, 10,000 random SNPs sets were generated for the tested lncRNAs. Compared to the 10,000 randomly selected SNP sets, the enrichments of 1,084 lncRNAs including 1,571 SNPs were higher for milk ability trait with enrichment fold 3.9490 (p = 0.0524), and the enrichments of 286 DE lncRNAs containing 485 SNPs were significantly enriched with GWAS signals of milk ability (p = 0.0116), and the enrichment fold was 5.0546.

### Prediction of target genes

To investigate probable roles of the lncRNAs in swine mammary gland, we predicted the target genes of lncRNAs. First, a search for protein-coding genes within 100 kb upstream/downstream of lncRNAs, and 6,589 genes were identified. Further, the expressions correlation between lncRNAs and protein-coding genes were examined, and 804 significant lncRNA-mRNA correlation pairs (p<0.05) were detected, including 685 protein coding genes and 443 lncRNAs. Among the 443 lncRNAs, 149 were DE, including 5, 41, 94, and 109 DE lncRNAs respectively corresponded to −10 vs −14, −6 vs −14, −2 vs −14, and +1 vs −14 (Supplementary Table S4).

In addition, we found that 256 protein-coding genes within 100 kb upstream/downstream of the 149 DE lncRNAs were also confirmed to be strongly correlated with them. To assess the function of these 286 DE lncRNAs, GO and KEGG enrichment analyses of the target genes were performed with KOBAS. There were 311 significant enrichments were presented (p<0.05; Supplementary Table S5), including 298 GO terms and 13 KEGG pathways. These GO and KEGG enrichments were involved in 87 genes, and most of them were directly related to the labor and colostrum. For example, CSN3, SMO, and CSN2 were involved in mammary associated activities. In addition, we performed functional annotation of the lncRNAs in cluster 4, 7, 9, 10 and 12, and found that 107 significant enrichments (p<0.05; Supplementary Table S6) were mainly involved in the process from colostrogenesis to lactogenesis, such as tight junction, response to progesterone, lactation, and ATP binding, which impact.

### Module construction

**Figure 5:**
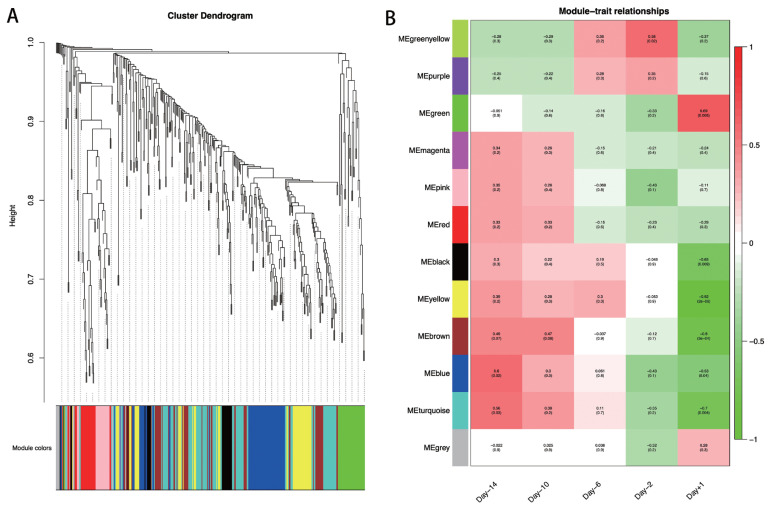
WGCNA at each swine mammary gland development. (A) Hierarchical cluster tree showing co-expression modules identified by WGCNA. (B) Module-sample relationships. Each column indicates a sample, and each row represents a module. The correlation coefficient and p-value of each sample-module relationship are displayed. Red represents high correlation value, and green represents low correlation value. WGCNA, weighted gene co-expression network analysis.

To explore the specific lncRNAs or genes highly associated with lactation, we performed WGCNA using DE lncRNAs and their target genes. A total of 12 modules associated with the specific expression profiles of different samples were identified (Figure 5A). Then, we calculated associations of each module with five lactation stages, and found seven modules including Greenyellow, Green, Black, Yellow, Brown, Blue, and Turquoise, were significantly associated with day −14, day −2, and day +1 (p<0.05; Figure 5B). In detail, the Greenyellow module, including 12 lncRNAs and 6 target genes were highly associated with day −2 (p = 0.02). The Green (including 5 lncRNAs and 30 target genes), Black (including 1 lncRNAs and 23 target genes), Yellow (including 5 lncRNAs and 49 target genes), and Brown (including 1 lncRNAs and 53 target genes) modules were significantly associated with the mammary gland samples at day +1 (p = 3E-04 to 0.009). The Turquoise (including 9 lncRNAs and 91 target genes) and Blue (including 2 lncRNAs and 67 target genes) modules were strongly associated with at day −14 and day +1 (p = 0.004 to 0.04). The lncRNAs and target genes involved in the significant modules are shown in Supplementary Table S7.

### Comprehensive analysis

**Figure 6:**
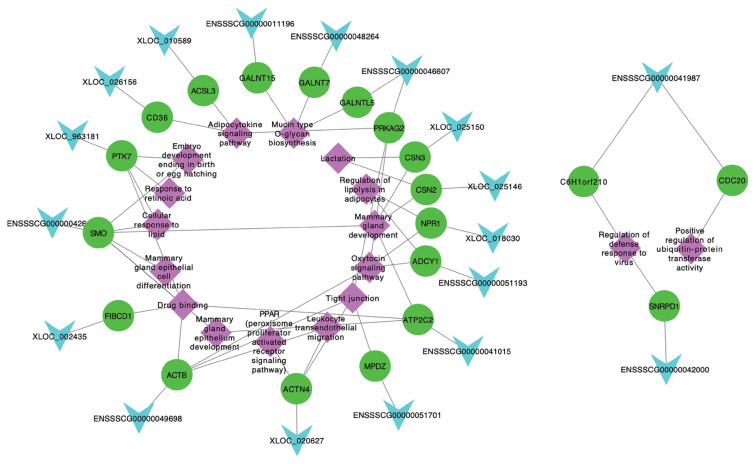
Network plot of the promising candidate lncRNAs, genes and pathways. The light blue, green and purple indicate lncRNAs, genes and pathways, respectively.

Among 256 target genes, 121 were DE across five lactation stages, and function annotation revealed that 25 of them targeted by 23 lncRNAs were involved in lactation developing, milk fat metabolism and colostrum function (Table 1). Interestingly, 20 target genes (*ACTN4*, *ADCY1*, *CSN3*, *SMO*, *PTK7*, *MPDZ*, *NPR1*, *CSN2*, *ATP2C2*, *PRKAG2*, *CD36*, *NPR1*, *ACSL3*, *GALNT15*, *C6H1orf210*, *CDC20*, *SNRPD1*, *FIBCD1*, *ACTB*, and *GALNT7*) and 3 lncRNAs (*XLOC_025150*, *ENSSS CG00000011196*, and *XLOC_010589*) also belong to significant modules by WGCNA. These 20 target genes corresponded to 18 lncRNAs, which also included the 3 lncRNAs (*XLOC_ 025150*, *ENSSSCG00000011196*, and *XLOC_010589*) involved in significant modules. Hence, we suggested that these 18 lncRNAs targeting 20 genes were the candidates involved in lactation of sows, which were considered as the potentially functional candidates impacting mammary gland development from colostrogenesis to lactogenesis. In addition, we conducted the network of promising candidate lncRNAs, genes and pathways (Figure 6), in which, the functions of lncRNAs were clearly shown. For example, XLOC_025150 might regulate *CSN3* gene involved in the mammary gland development and lactation.

## DISCUSSION

In this study, we systematically analyzed the RNA-seq data of 15 swine mammary gland samples collected at days 14, 10, 6, and 2 before (−) parturition to day 1 after (+) parturition and identified a total of 286 DE lncRNAs targeting 256 genes. Further, we found that these DE lncRNAs had significant association with sow lactation, and were involved in delivery and lactation development, milk lipid metabolism, and immune function of colostrum, and 18 lncRNAs targeting 20 genes which were proposed to be the candidates involved in lactation of sow.

At parturition, the preparation for copious milk synthesis and secretion have begun [7,26], and the mammary gland can reach the greatest degree of structural development. In this study, we performed the validation of GWAS signals, and showed that the DE lncRNAs were significantly enriched with GWAS signals of sow milk ability, which indicated that these dynamically changed lncRNAs in different periods of mammary gland progression may be involved in milk ability. We examined the expression pattern of DE lncRNAs, most of the lncRNAs expressions were strongly changed from day −6 to day +1. Further, 18 promising functional lncRNAs targeting 20 genes were mainly identified in −2 vs −14 and 1 vs −14 groups, which reflected a strong activation of many metabolic processes before and after parturition. In the WGCNA, 20 promising functional DE target genes were involved in the significant modules, which were highly associated with the mammary gland samples at day −14, day −2, and day +1. Hence, our results were consistent with the concept: before and after farrowing with the formation of colostrum, mammary glands immediately undergo strong functional differentiation [5].

In general, lncRNAs exert regulatory functions at different levels of gene expression to influence the tumor growth, cell-cycle, and apoptosis [24,27]. For example, lncRNA H19 is involved in regulation of high glucose-induced apoptosis through targeting VDAC1 [28]. Here, 11 lncRNA-mRNA target pairs, *XLOC_020627-ACTN4*, *ENSSSCG00000051193-ADCY1*, *XLOC_025150-CSN3*, *ENSSSCG00000042618-SMO*, *XLOC_963181-PTK7*, *ENSSSCG00000051701-MPDZ*, *XLOC_018030-NPR1*, *XLOC_025146-CSN2*, *ENSSSCG00 000041015-ATP2C2*, *ENSSSCG00000046607-PRKAG2*, and *ENSSSCG00000049698-ACTB*, were involved in delivery and lactation metabolism, such as tight junctions, oxytocin, development of the mammary gland and lactation. Tight junctions of mammary gland from the pregnant animal are leaky, undergoing closure around parturition to become the impermeable tight junctions of the lactating animal [29]. In dairy cattle, after parturition, the start of copious milk production requires the closure of tight junctions to form the blood-milk barrier and prevent paracellular transfer of blood constituents into milk (such as lactate dehydrogenase and serum albumin) and vice versa (such as appearance of α-lactalbumin in blood) [30]. Oxytocin is a nonapeptide hormone that has a central role in the regulation of parturition and lactation [31], and its best-known and most well-established roles are stimulation of uterine contractions during parturition and milk release during lactation [32]. Development of the mammary gland occurs in defined stages, which are connected to sexual development and reproduction (embryonic, prepubertal, pubertal, pregnancy, lactation, and involution) [33]. Lactation is a highly demanding lipid synthesis and transport process that is crucial for the development of newborn mammals [34]. Embryo development ending in birth or egg hatching term was defined as the process whose specific outcome is the progression of an embryo over time, from zygote formation until the end of the embryonic life stage, and for mammals it is usually considered to be birth. The above reports suggested that these GO and KEGG enrichments were related with the parturition and lactation. Hence, we proposed that these lncRNAs in 11 lncRNA-mRNA target pairs might be regulated their target genes to impact the delivery and lactation processes.

Additionally, we found eight lncRNA-mRNA target pairs, *XLOC_020627-ACTN4*, *ENSSSCG00000051193-ADCY1*, *ENSSSCG00000042618-SMO*, *XLOC_963181-PTK7*, *XLOC_ 026156-CD36*, *XLOC_018030-NPR1*, *XLOC_010589-ACSL3*, and *ENSSSCG00000046607-PRKAG2*, were enriched in peroxisome proliferator-activated receptors (PPARs), regulation of lipolysis in adipocytes, cellular response to lipid, and adipocytokine signaling pathways, that might be related with the milk components metabolism.

For the piglets, colostrum plays an important role in ensuring their survival, growth and health by providing energy, nutrients, immunoglobulins, growth factors and many other bioactive components and cells [35]. In the present study, we found that 12 lncRNA-mRNA target pairs, *XLOC_020627-ACTN4*, *ENSSSCG00000042618-SMO*, *XLOC_963181-PTK7*, *ENSSSCG00000011196-GALNT15*, *ENSSSCG00000041987-C6H1orf210*, *ENSSSCG00000041987-CDC20*, *ENSSSCG 00000041015-ATP2C2*, *ENSSSCG00000046607-GALNTL5*, *ENSSSCG00000042000-SNRPD1*, *XLOC_002435-FIBCD1*, *ENSSSCG00000049698-ACTB*, and *ENSSSCG000000482 64-GALNT7*, were involved in leukocyte transendothelial migration, response to retinoic acid, drug binding, mucin type O-glycan biosynthesis, regulation of defense response to virus, and positive regulation of ubiquitin-protein transferase activity enrichments, which might impact the immune function of colostrum.

Based on these advantages, we proposed these 18 lncRNAs (*XLOC_020627, ENSSSCG00000051193*, *XLOC_025150*, *ENSSSCG00000042618*, *XLOC_963181*, *ENSSSCG000000 51701*, *XLOC_018030, XLOC_025146*, *ENSSSCG00000041015*, *ENSSSCG00000046607*, *ENSSSCG00000049698*, *XLOC_ 026156, XLOC_010589*, *ENSSSCG00000011196*, *ENSSSCG 00000041987*, *ENSSSCG00000042000*, *XLOC_002435*, and *ENSSSCG00000048264*) as the promising candidates for swine parturition and lactation. Our network results of 18 lncRNAs, their 20 target genes and corresponding pathways further clearly showed the functions of the candidate lcnRNAs and their target genes. There is a high probability that these lncRNAs and genes interact closely and thus act as key regulators in mammary gland, thus, future work should focus on the potential roles of them in sow lactation.

Sow milk production is the major factor which can limit growth and survival of piglets. To enhance milk performance, it is necessary to understand the process of mammary gland morphogenesis and to identify the regulatory factors of mammary development. In this study, we identified 1,084 lncRNAs in swine mammary gland using RNA-seq data, and 286 were DE lncRNAs in five lactation stages. Further, the enrichment analysis of GWAS signals for sow milk ability trait validated that the identified DE lncRNAs had significant association with milk ability trait. Integrated analysis of the DE lncRNAs expression pattern examination, targets prediction, function annotation and WGCNA, we proposed that 18 lncRNAs (such as *XLOC_020627*, *ENSSSCG00000051193*, *XLOC_025150*, *ENSSSCG00000042618*, *XLOC_963181*, *ENSSSCG000000 51701*, *XLOC_018030*, and *XLOC_025146*) targeting 20 genes (such as *ACTN4*, *ADCY1*, *CSN3*, *SMO*, *CSN2*, *PRKAG2*, *FIBCD1*, and *GALNT7*) as promising candidates which were involved in swine parturition and colostrum occurrence processes. These results provided a new insight for exploring critically regulatory factors involved in reproductive performance of sow.

## Figures and Tables

**Table 1:** GO and KEGG enrichments of 23 potentially functional lncRNAs and their 25 target genes

LncRNA	Group	Target gene	ID	Term	p-value
XLOC_020627	−2vs−14	*ACTN4* ^*^	ssc04530	Tight junction	1.75E-03
			GO:0035357	peroxisome proliferator activated receptor signaling pathway	4.03E-02
			ssc04670	Leukocyte transendothelial migration	1.92E-02
ENSSSCG00000051193	1vs−14	*ADCY1* ^*^	ssc04921	Oxytocin signaling pathway	6.73E-03
			ssc04923	Regulation of lipolysis in adipocytes	3.09E-02
XLOC_025150^*^	−10vs−14, −6vs−14, −2vs−14, 1vs−14	*CSN3* ^*^	GO:0030879	mammary gland development	1.49E-06
			GO:0007595	lactation	1.48E-03
ENSSSCG00000042618	−2vs−14	*SMO* ^*^	GO:0030879	mammary gland development	1.49E-06
			GO:0061180	mammary gland epithelium development	2.94E-03
			GO:0060644	mammary gland epithelial cell differentiation	3.46E-02
			GO:0009792	embryo development ending in birth or egg hatching	4.80E-02
			GO:0071396	cellular response to lipid	2.42E-02
			GO:0008144	drug binding	3.27E-02
XLOC_963181	−2vs−14, 1vs−14	*PTK7* ^*^	GO:0009792	embryo development ending in birth or egg hatching	4.80E-02
			GO:0071396	cellular response to lipid	2.42E-02
			GO:0008144	drug binding	3.27E-02
			GO:0032526	response to retinoic acid	4.35E-03
XLOC_026156	−10vs−14, −6vs−14, −2vs−14, 1vs−14	*CD36* ^*^	ssc04920	Adipocytokine signaling pathway	6.51E-03
ENSSSCG00000011196^*^	−2vs−14, 1vs−14	*GALNT15* ^*^	ssc00512	Mucin type O-glycan biosynthesis	6.33E-04
ENSSSCG00000036096	−6vs−14, −2vs−14, 1vs−14	*CLDN6*	ssc04530	Tight junction	1.75E-03
			ssc04670	Leukocyte transendothelial migration	1.92E-02
ENSSSCG00000042682	−6vs−14	*RHBDF2*	GO:0038127	ERBB signaling pathway	4.87E-03
			GO:0007173	epidermal growth factor receptor signaling pathway	3.85E-03
ENSSSCG00000041987	−6vs−14, −2vs−14, 1vs−14	*C6H1orf210* ^*^	GO:0050688	regulation of defense response to virus	1.50E-02
		*CDC20* ^*^	GO:0051443	positive regulation of ubiquitin-protein transferase activity	4.03E-02
ENSSSCG00000051701	−2vs−14, 1vs−14	*MPDZ* ^*^	ssc04530	Tight junction	1.75E-03
XLOC_018030	−2vs−14	*NPR1* ^*^	ssc04921	Oxytocin signaling pathway	6.73E-03
			ssc04923	Regulation of lipolysis in adipocytes	3.09E-02
XLOC_025146	−10vs−14, −6vs−14, −2vs−14, 1vs−14	*CSN2* ^*^	GO:0030879	mammary gland development	1.49E-06
			GO:0007595	lactation	1.48E-03
ENSSSCG00000041015	1vs−14	*ATP2C2* ^*^	GO:0030879	mammary gland development	1.49E-06
			GO:0061180	mammary gland epithelium development	2.94E-03
			GO:0008144	drug binding	3.27E-02
XLOC_005878	−6vs−14, −2vs−14	*MEOX1*	GO:0009792	embryo development ending in birth or egg hatching	4.80E-02
XLOC_010589^*^	−2vs−14, 1vs−14	*ACSL3* ^*^	ssc04920	Adipocytokine signaling pathway	6.51E-03
ENSSSCG00000046607	−6vs−14, −2vs−14, 1vs−14	*GALNTL5* ^*^	ssc00512	Mucin type O-glycan biosynthesis	6.33E-04
		*PRKAG2* ^*^	ssc04530	Tight junction	1.75E-03
			ssc04921	Oxytocin signaling pathway	6.73E-03
			ssc04920	Adipocytokine signaling pathway	6.51E-03
ENSSSCG00000050015	−6vs−14, −2vs−14, 1vs−14	*SOX9*	GO:0030879	mammary gland development	1.49E-06
			GO:0071396	cellular response to lipid	2.42E-02
			GO:0038127	ERBB signaling pathway	4.87E-03
			GO:0032526	response to retinoic acid	4.35E-03
			GO:0007173	epidermal growth factor receptor signaling pathway	3.85E-03
ENSSSCG00000042000	−2vs−14, 1vs−14	*SNRPD1* ^*^	GO:0050688	regulation of defense response to virus	1.50E-02
XLOC_002435	−2vs−14	*FIBCD1* ^*^	GO:0008144	drug binding	3.27E-02
ENSSSCG00000049698	−2vs−14	*ACTB* ^*^	ssc04530	Tight junction	1.75E-03
			ssc04921	Oxytocin signaling pathway	6.73E-03
			ssc04670	Leukocyte transendothelial migration	1.92E-02
			GO:0008144	drug binding	3.27E-02
ENSSSCG00000048264	1vs−14	*GALNT7* ^*^	ssc00512	Mucin type O-glycan biosynthesis	6.33E-04
XLOC_003621	1vs−14	*MAP3K8*	GO:0008144	drug binding	3.27E-02

GO, gene ontology; KEGG, Kyoto encyclopedia of genes and genomes.

* Indicates the lncRNAs and their target genes involved in the significant module by WGCNA.

## SUPPLEMENTARY MATERIAL

**Supplementary Figure S1**. The result of gap statistics analysis. K = 14 was the optimal choice for cluster analysis. **Supplementary Table S1**. The information of RNA sequencing data for 15 samples of sow mammary gland tissue **Supplementary Table S2**. The results of the identified lncRNAs in this study **Supplementary Table S3**. The detailed information of DE lncRNAs in this study **Supplementary Table S4**. The predicted target genes of DE lncRNAs **Supplementary Table S5**. Significant GO and KEGG pathways of the target genes of DE lncRNAs **Supplementary Table S6**. Significant GO and KEGG pathways of lncRNAs in cluster 4, 7, 9, 10 and 12 **Supplementary Table S7**. The lncRNAs and genes involved in significant modules
