# Supplementary material for: Identifying long non-coding RNAs and characterizing their functional roles in swine mammary gland from colostrogenesis to lactogenesis
Source: Anim Biosci. 2021 Oct 29;35(6):814–25. doi: 10.5713/ab.21.0308 (PMC9066039; doi:10.5713/ab.21.0308)
Supplement: Supplementary file 1 [file ab-21-0308-suppl.pdf]

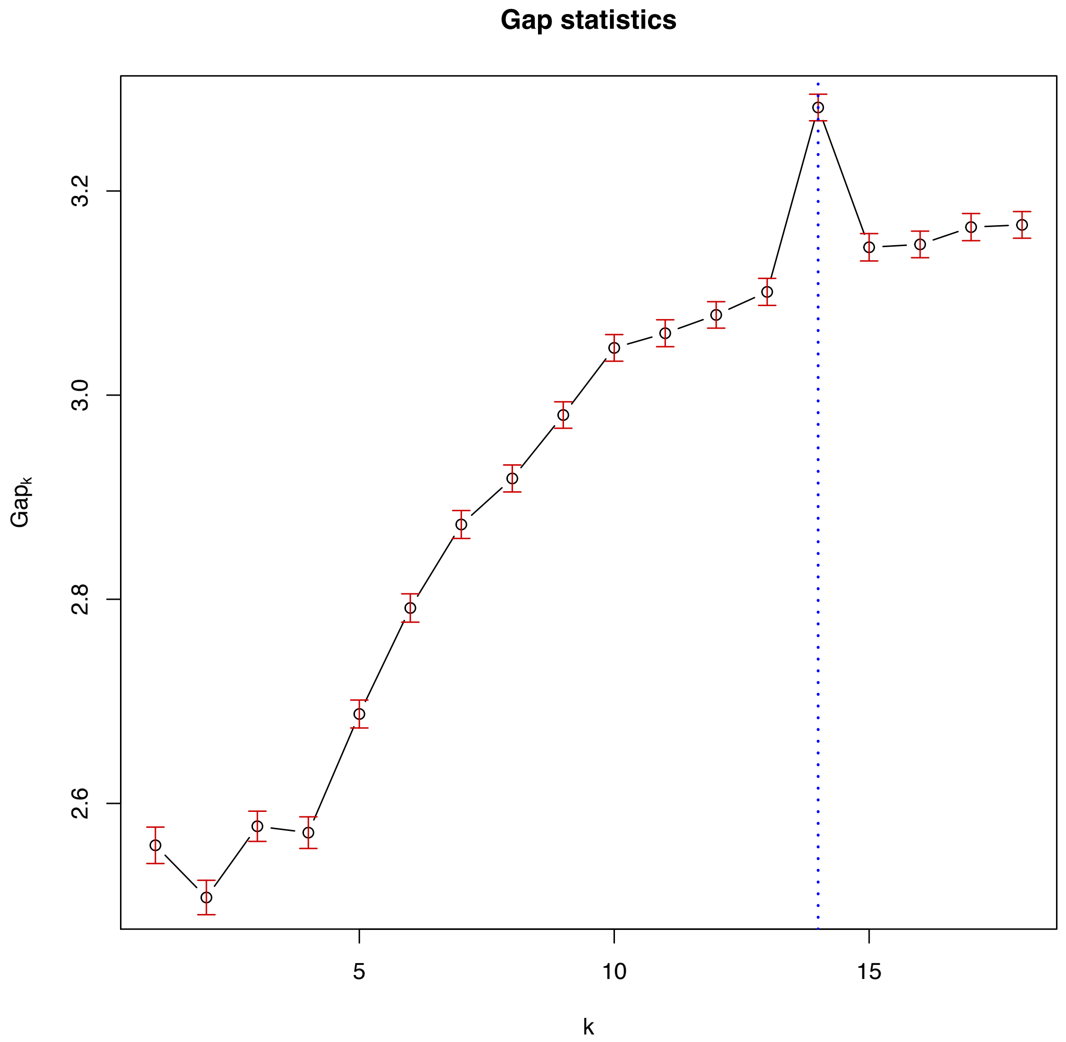

Figure S1. The result of gap statistics analysis. K = 14 was the optimal choice for cluster analysis.

Table S1 The information of RNA sequencing data for 15 samples of sow mammary gland tissue

Series	Sample name	SRA run	Time
GSE101983	GSM2720056	SRR5877105	(-) 14 days to parturition
GSE101983	GSM2720057	SRR5877106	(-) 14 days to parturition
GSE101983	GSM2720058	SRR5877107	(-) 14 days to parturition
GSE101983	GSM2720059	SRR5877108	(-) 10 days to parturition
GSE101983	GSM2720060	SRR5877109	(-) 10 days to parturition
GSE101983	GSM2720061	SRR5877110	(-) 10 days to parturition
GSE101983	GSM2720062	SRR5877111	(-) 6 days to parturition
GSE101983	GSM2720063	SRR5877112	(-) 6 days to parturition
GSE101983	GSM2720064	SRR5877113	(-) 6 days to parturition
GSE101983	GSM2720065	SRR5877114	(-) 2 days to parturition
GSE101983	GSM2720066	SRR5877115	(-) 2 days to parturition
GSE101983	GSM2720067	SRR5877116	(-) 2 days to parturition
GSE101983	GSM2720068	SRR5877117	(+) 1 days after parturition
GSE101983	GSM2720069	SRR5877118	(+) 1 days after parturition
GSE101983	GSM2720070	SRR5877119	(+) 1 days after parturition

Table S2 The results of the identified lncRNAs in this study

Annotated lncRNA transcripts	Annotated lncRNAs	Novel lncRNA transcripts	Novel lncRNAs
ENSSSCT00000066540	ENSSSCG00000048769	TCONS_00000129	XLOC_000152
ENSSSCT00000078085	ENSSSCG00000048769	TCONS_00001277	XLOC_000605
ENSSSCT00000073528	ENSSSCG00000047676	TCONS_00003139	XLOC_001405
ENSSSCT00000068617	ENSSSCG00000047753	TCONS_00003197	XLOC_002603
ENSSSCT00000086080	ENSSSCG00000045280	TCONS_00004909	XLOC_000142
ENSSSCT00000067997	ENSSSCG00000043317	TCONS_00004966	XLOC_000227
ENSSSCT00000074506	ENSSSCG00000043317	TCONS_00005608	XLOC_001291
ENSSSCT00000069752	ENSSSCG00000043814	TCONS_00006263	XLOC_002263
ENSSSCT00000078832	ENSSSCG00000042025	TCONS_00006291	XLOC_002326
ENSSSCT00000075399	ENSSSCG00000048821	TCONS_00006959	XLOC_002632
ENSSSCT00000077375	ENSSSCG00000047176	TCONS_00007474	XLOC_002757
ENSSSCT00000082959	ENSSSCG00000047176	TCONS_00008487	XLOC_000422
ENSSSCT00000085390	ENSSSCG00000046537	TCONS_00008600	XLOC_004323
ENSSSCT00000083240	ENSSSCG00000041354	TCONS_00008830	XLOC_001196
ENSSSCT00000087586	ENSSSCG00000041354	TCONS_00010924	XLOC_000412
ENSSSCT00000076987	ENSSSCG00000050757	TCONS_00011216	XLOC_001196
ENSSSCT00000083341	ENSSSCG00000046259	TCONS_00011779	XLOC_002435
ENSSSCT00000083864	ENSSSCG00000045292	TCONS_00011901	XLOC_020212
ENSSSCT00000069130	ENSSSCG00000050451	TCONS_00012647	XLOC_003100
ENSSSCT00000079258	ENSSSCG00000047571	TCONS_00013100	XLOC_001323
ENSSSCT00000069913	ENSSSCG00000042663	TCONS_00013363	XLOC_002035
ENSSSCT00000086255	ENSSSCG00000044841	TCONS_00014708	XLOC_000993
ENSSSCT00000079762	ENSSSCG00000044841	TCONS_00016390	XLOC_001196
ENSSSCT00000075287	ENSSSCG00000046117	TCONS_00017118	XLOC_050440
ENSSSCT00000071809	ENSSSCG00000004324	TCONS_00018064	XLOC_001339
ENSSSCT00000004780	ENSSSCG00000004324	TCONS_00018770	XLOC_002768
ENSSSCT00000077054	ENSSSCG00000049906	TCONS_00020236	XLOC_067664
ENSSSCT00000087712	ENSSSCG00000050067	TCONS_00020889	XLOC_000864
ENSSSCT00000081336	ENSSSCG00000050067	TCONS_00022026	XLOC_000221
ENSSSCT00000082451	ENSSSCG00000047800	TCONS_00028628	XLOC_003145
ENSSSCT00000066182	ENSSSCG00000034415	TCONS_00029071	XLOC_003504
ENSSSCT00000084100	ENSSSCG00000044010	TCONS_00029369	XLOC_003729
ENSSSCT00000081445	ENSSSCG00000044010	TCONS_00029374	XLOC_003732
ENSSSCT00000087822	ENSSSCG00000041006	TCONS_00029531	XLOC_003829
ENSSSCT00000077378	ENSSSCG00000049706	TCONS_00029629	XLOC_003895
ENSSSCT00000070523	ENSSSCG00000042551	TCONS_00030235	XLOC_003749
ENSSSCT00000067513	ENSSSCG00000046685	TCONS_00030955	XLOC_003864
ENSSSCT00000075833	ENSSSCG00000041906	TCONS_00031132	XLOC_003366
ENSSSCT00000049397	ENSSSCG00000034069	TCONS_00031226	XLOC_000426
ENSSSCT00000078556	ENSSSCG00000034069	TCONS_00031929	XLOC_003300
ENSSSCT00000084175	ENSSSCG00000039179	TCONS_00032589	XLOC_003893

ENSSSCT00000081223	ENSSSCG00000049765	TCONS_00032860	XLOC_003680
ENSSSCT00000090797	ENSSSCG00000049234	TCONS_00033493	XLOC_003680
ENSSSCT00000078054	ENSSSCG00000043563	TCONS_00033584	XLOC_003177
ENSSSCT00000070833	ENSSSCG00000042837	TCONS_00033850	XLOC_003857
ENSSSCT00000005615	ENSSSCG00000005094	TCONS_00034557	XLOC_003177
ENSSSCT00000080855	ENSSSCG00000051482	TCONS_00034724	XLOC_003621
ENSSSCT00000083909	ENSSSCG00000051482	TCONS_00035069	XLOC_004032
ENSSSCT00000088260	ENSSSCG00000043775	TCONS_00035151	XLOC_016233
ENSSSCT00000083691	ENSSSCG00000042822	TCONS_00035237	XLOC_004148
ENSSSCT00000069827	ENSSSCG00000051701	TCONS_00036411	XLOC_004473
ENSSSCT00000080740	ENSSSCG00000042369	TCONS_00036864	XLOC_004325
ENSSSCT00000089563	ENSSSCG00000049199	TCONS_00037040	XLOC_004554
ENSSSCT00000076844	ENSSSCG00000047129	TCONS_00037574	XLOC_004183
ENSSSCT00000073269	ENSSSCG00000048111	TCONS_00037710	XLOC_004530
ENSSSCT00000080149	ENSSSCG00000046947	TCONS_00037793	XLOC_004057
ENSSSCT00000076471	ENSSSCG00000046947	TCONS_00037999	XLOC_004472
ENSSSCT00000073304	ENSSSCG00000045435	TCONS_00038404	XLOC_004163
ENSSSCT00000091103	ENSSSCG00000051033	TCONS_00040278	XLOC_004710
ENSSSCT00000012261	ENSSSCG00000011196	TCONS_00040383	XLOC_004739
ENSSSCT00000051710	ENSSSCG00000033794	TCONS_00040385	XLOC_004739
ENSSSCT00000080892	ENSSSCG00000051746	TCONS_00040386	XLOC_004742
ENSSSCT00000072226	ENSSSCG00000048558	TCONS_00040387	XLOC_004739
ENSSSCT00000073637	ENSSSCG00000042530	TCONS_00041422	XLOC_005186
ENSSSCT00000086990	ENSSSCG00000051293	TCONS_00041729	XLOC_005324
ENSSSCT00000076099	ENSSSCG00000045880	TCONS_00041734	XLOC_005330
ENSSSCT00000071995	ENSSSCG00000044633	TCONS_00041735	XLOC_005330
ENSSSCT00000072199	ENSSSCG00000051313	TCONS_00041736	XLOC_005330
ENSSSCT00000071753	ENSSSCG00000045563	TCONS_00041737	XLOC_005330
ENSSSCT00000079688	ENSSSCG00000048677	TCONS_00042224	XLOC_005604
ENSSSCT00000076147	ENSSSCG00000049696	TCONS_00042466	XLOC_005693
ENSSSCT00000076089	ENSSSCG00000045498	TCONS_00042467	XLOC_005693
ENSSSCT00000067714	ENSSSCG00000042151	TCONS_00042668	XLOC_005785
ENSSSCT00000080485	ENSSSCG00000044855	TCONS_00042734	XLOC_005809
ENSSSCT00000087787	ENSSSCG00000047636	TCONS_00042804	XLOC_005878
ENSSSCT00000077158	ENSSSCG00000042725	TCONS_00042894	XLOC_004713
ENSSSCT00000066468	ENSSSCG00000043503	TCONS_00042929	XLOC_004732
ENSSSCT00000087036	ENSSSCG00000034990	TCONS_00042985	XLOC_004739
ENSSSCT00000068134	ENSSSCG00000034990	TCONS_00043868	XLOC_005309
ENSSSCT00000070177	ENSSSCG00000034990	TCONS_00044082	XLOC_005499
ENSSSCT00000059401	ENSSSCG00000034990	TCONS_00044562	XLOC_005792
ENSSSCT00000081877	ENSSSCG00000034990	TCONS_00045971	XLOC_004742
ENSSSCT00000077830	ENSSSCG00000048298	TCONS_00046444	XLOC_005245
ENSSSCT00000091270	ENSSSCG00000051565	TCONS_00047068	XLOC_004739
ENSSSCT00000078878	ENSSSCG00000046757	TCONS_00047357	XLOC_005110

ENSSSCT00000068082	ENSSSCG00000049995	TCONS_00047676	XLOC_005563
ENSSSCT00000066898	ENSSSCG00000049995	TCONS_00050711	XLOC_002456
ENSSSCT00000077606	ENSSSCG00000046366	TCONS_00053069	XLOC_005330
ENSSSCT00000080548	ENSSSCG00000044764	TCONS_00053922	XLOC_005742
ENSSSCT00000083425	ENSSSCG00000044764	TCONS_00055325	XLOC_006468
ENSSSCT00000074665	ENSSSCG00000048579	TCONS_00055348	XLOC_006489
ENSSSCT00000074754	ENSSSCG00000042665	TCONS_00057521	XLOC_006071
ENSSSCT00000078761	ENSSSCG00000049319	TCONS_00059392	XLOC_006018
ENSSSCT00000079729	ENSSSCG00000043103	TCONS_00059815	XLOC_006468
ENSSSCT00000080321	ENSSSCG00000051023	TCONS_00061336	XLOC_006716
ENSSSCT00000089104	ENSSSCG00000041918	TCONS_00061562	XLOC_007135
ENSSSCT00000067542	ENSSSCG00000044255	TCONS_00061780	XLOC_007497
ENSSSCT00000079332	ENSSSCG00000044255	TCONS_00063311	XLOC_006501
ENSSSCT00000084199	ENSSSCG00000049399	TCONS_00063443	XLOC_006721
ENSSSCT00000030032	ENSSSCG00000027975	TCONS_00063667	XLOC_007183
ENSSSCT00000084152	ENSSSCG00000048463	TCONS_00065551	XLOC_007694
ENSSSCT00000082106	ENSSSCG00000051512	TCONS_00066773	XLOC_006647
ENSSSCT00000074808	ENSSSCG00000049117	TCONS_00067804	XLOC_007547
ENSSSCT00000082504	ENSSSCG00000041015	TCONS_00071197	XLOC_008034
ENSSSCT00000077147	ENSSSCG00000042332	TCONS_00071347	XLOC_008130
ENSSSCT00000085729	ENSSSCG00000046070	TCONS_00071948	XLOC_002995
ENSSSCT00000069021	ENSSSCG00000047270	TCONS_00072136	XLOC_008587
ENSSSCT00000077794	ENSSSCG00000047270	TCONS_00073028	XLOC_009155
ENSSSCT00000073890	ENSSSCG00000047270	TCONS_00073721	XLOC_009655
ENSSSCT00000074196	ENSSSCG00000047270	TCONS_00073980	XLOC_008257
ENSSSCT00000082754	ENSSSCG00000048405	TCONS_00075167	XLOC_009248
ENSSSCT00000086222	ENSSSCG00000041536	TCONS_00076434	XLOC_009020
ENSSSCT00000073811	ENSSSCG00000046766	TCONS_00076435	XLOC_009020
ENSSSCT00000072770	ENSSSCG00000044425	TCONS_00076967	XLOC_008121
ENSSSCT00000070604	ENSSSCG00000047349	TCONS_00077543	XLOC_008831
ENSSSCT00000074831	ENSSSCG00000051232	TCONS_00077765	XLOC_009155
ENSSSCT00000073489	ENSSSCG00000048693	TCONS_00077860	XLOC_009194
ENSSSCT00000090846	ENSSSCG00000051668	TCONS_00077861	XLOC_009194
ENSSSCT00000085032	ENSSSCG00000051668	TCONS_00077862	XLOC_009194
ENSSSCT00000078798	ENSSSCG00000042051	TCONS_00078746	XLOC_009020
ENSSSCT00000082317	ENSSSCG00000047379	TCONS_00078747	XLOC_009020
ENSSSCT00000090691	ENSSSCG00000045200	TCONS_00079671	XLOC_009155
ENSSSCT00000067728	ENSSSCG00000045200	TCONS_00086453	XLOC_009790
ENSSSCT00000088606	ENSSSCG00000042769	TCONS_00086475	XLOC_009810
ENSSSCT00000072429	ENSSSCG00000046897	TCONS_00086787	XLOC_010030
ENSSSCT00000041219	ENSSSCG00000032594	TCONS_00087583	XLOC_010463
ENSSSCT00000051515	ENSSSCG00000032594	TCONS_00088297	XLOC_009745
ENSSSCT00000085449	ENSSSCG00000050217	TCONS_00088555	XLOC_010030
ENSSSCT00000079047	ENSSSCG00000041123	TCONS_00089942	XLOC_010310

ENSSSCT00000090934	ENSSSCG00000046890	TCONS_00091254	XLOC_010813
ENSSSCT00000072379	ENSSSCG00000047234	TCONS_00091792	XLOC_010506
ENSSSCT00000077341	ENSSSCG00000041238	TCONS_00093647	XLOC_010589
ENSSSCT00000089388	ENSSSCG00000041160	TCONS_00094988	XLOC_010253
ENSSSCT00000075367	ENSSSCG00000041160	TCONS_00097609	XLOC_011232
ENSSSCT00000079664	ENSSSCG00000050419	TCONS_00097643	XLOC_011266
ENSSSCT00000081743	ENSSSCG00000049887	TCONS_00101777	XLOC_111090
ENSSSCT00000073217	ENSSSCG00000041030	TCONS_00102639	XLOC_012071
ENSSSCT00000072943	ENSSSCG00000041487	TCONS_00102688	XLOC_012097
ENSSSCT00000070520	ENSSSCG00000043238	TCONS_00103139	XLOC_012336
ENSSSCT00000083041	ENSSSCG00000051473	TCONS_00103192	XLOC_012374
ENSSSCT00000080181	ENSSSCG00000047361	TCONS_00103328	XLOC_012406
ENSSSCT00000071282	ENSSSCG00000047147	TCONS_00103476	XLOC_012457
ENSSSCT00000074148	ENSSSCG00000047147	TCONS_00103632	XLOC_011817
ENSSSCT00000076852	ENSSSCG00000042438	TCONS_00104066	XLOC_012238
ENSSSCT00000084868	ENSSSCG00000041401	TCONS_00104750	XLOC_012080
ENSSSCT00000087236	ENSSSCG00000042252	TCONS_00104968	XLOC_012336
ENSSSCT00000084442	ENSSSCG00000049071	TCONS_00104970	XLOC_012336
ENSSSCT00000075690	ENSSSCG00000048434	TCONS_00105688	XLOC_012407
ENSSSCT00000091090	ENSSSCG00000048434	TCONS_00106033	XLOC_012182
ENSSSCT00000077678	ENSSSCG00000049192	TCONS_00106684	XLOC_012536
ENSSSCT00000090260	ENSSSCG00000030438	TCONS_00106958	XLOC_012336
ENSSSCT00000087513	ENSSSCG00000030438	TCONS_00106959	XLOC_012336
ENSSSCT00000074599	ENSSSCG00000047925	TCONS_00107343	XLOC_012336
ENSSSCT00000080668	ENSSSCG00000049166	TCONS_00107675	XLOC_012336
ENSSSCT00000077194	ENSSSCG00000051754	TCONS_00107676	XLOC_012336
ENSSSCT00000072492	ENSSSCG00000051754	TCONS_00107679	XLOC_012336
ENSSSCT00000076052	ENSSSCG00000048215	TCONS_00107680	XLOC_012336
ENSSSCT00000068004	ENSSSCG00000051726	TCONS_00108302	XLOC_012082
ENSSSCT00000073494	ENSSSCG00000044910	TCONS_00108415	XLOC_012336
ENSSSCT00000086706	ENSSSCG00000042000	TCONS_00109419	XLOC_012200
ENSSSCT00000074269	ENSSSCG00000045581	TCONS_00109953	XLOC_012588
ENSSSCT00000067031	ENSSSCG00000043832	TCONS_00109954	XLOC_012591
ENSSSCT00000085878	ENSSSCG00000041327	TCONS_00109955	XLOC_012591
ENSSSCT00000081457	ENSSSCG00000047167	TCONS_00109958	XLOC_012591
ENSSSCT00000038686	ENSSSCG00000036505	TCONS_00111003	XLOC_012786
ENSSSCT00000067017	ENSSSCG00000047088	TCONS_00112043	XLOC_012823
ENSSSCT00000071698	ENSSSCG00000050815	TCONS_00112204	XLOC_013095
ENSSSCT00000088461	ENSSSCG00000041959	TCONS_00112270	XLOC_013215
ENSSSCT00000090297	ENSSSCG00000051147	TCONS_00112308	XLOC_013304
ENSSSCT00000086257	ENSSSCG00000042401	TCONS_00112394	XLOC_012730
ENSSSCT00000079963	ENSSSCG00000046729	TCONS_00112969	XLOC_012823
ENSSSCT00000075854	ENSSSCG00000045223	TCONS_00113318	XLOC_013083
ENSSSCT00000071645	ENSSSCG00000046815	TCONS_00113740	XLOC_012977

ENSSSCT00000074623	ENSSSCG00000041987	TCONS_00115567	XLOC_013521
ENSSSCT00000076505	ENSSSCG00000041170	TCONS_00115632	XLOC_013551
ENSSSCT00000062826	ENSSSCG00000037852	TCONS_00116580	XLOC_014000
ENSSSCT00000075249	ENSSSCG00000038005	TCONS_00116828	XLOC_014131
ENSSSCT00000088968	ENSSSCG00000044406	TCONS_00116832	XLOC_014132
ENSSSCT00000074263	ENSSSCG00000044406	TCONS_00116833	XLOC_014132
ENSSSCT00000090567	ENSSSCG00000044406	TCONS_00117061	XLOC_014265
ENSSSCT00000014044	ENSSSCG00000044406	TCONS_00117481	XLOC_001405
ENSSSCT00000088022	ENSSSCG00000044406	TCONS_00117496	XLOC_014527
ENSSSCT00000081255	ENSSSCG00000050649	TCONS_00118357	XLOC_015041
ENSSSCT00000078829	ENSSSCG00000050649	TCONS_00118506	XLOC_015106
ENSSSCT00000078300	ENSSSCG00000044638	TCONS_00118555	XLOC_015127
ENSSSCT00000076335	ENSSSCG00000049480	TCONS_00119001	XLOC_013440
ENSSSCT00000077709	ENSSSCG00000043204	TCONS_00121302	XLOC_013325
ENSSSCT00000084832	ENSSSCG00000049464	TCONS_00121394	XLOC_013419
ENSSSCT00000089109	ENSSSCG00000044730	TCONS_00123812	XLOC_014191
ENSSSCT00000088188	ENSSSCG00000047432	TCONS_00125238	XLOC_014132
ENSSSCT00000077869	ENSSSCG00000048856	TCONS_00125239	XLOC_014132
ENSSSCT00000080860	ENSSSCG00000048856	TCONS_00125581	XLOC_014650
ENSSSCT00000079369	ENSSSCG00000048856	TCONS_00127312	XLOC_013602
ENSSSCT00000069110	ENSSSCG00000041050	TCONS_00127676	XLOC_014131
ENSSSCT00000082209	ENSSSCG00000041050	TCONS_00128316	XLOC_013379
ENSSSCT00000090294	ENSSSCG00000044913	TCONS_00133930	XLOC_014830
ENSSSCT00000081373	ENSSSCG00000043175	TCONS_00136321	XLOC_015687
ENSSSCT00000073945	ENSSSCG00000041814	TCONS_00136322	XLOC_015687
ENSSSCT00000090258	ENSSSCG00000043447	TCONS_00137358	XLOC_016230
ENSSSCT00000090326	ENSSSCG00000043766	TCONS_00137928	XLOC_016545
ENSSSCT00000072626	ENSSSCG00000048886	TCONS_00138000	XLOC_016592
ENSSSCT00000082187	ENSSSCG00000044035	TCONS_00138014	XLOC_016600
ENSSSCT00000088971	ENSSSCG00000044088	TCONS_00138089	XLOC_016636
ENSSSCT00000075261	ENSSSCG00000043074	TCONS_00138476	XLOC_016878
ENSSSCT00000078922	ENSSSCG00000049971	TCONS_00139013	XLOC_015558
ENSSSCT00000077811	ENSSSCG00000045617	TCONS_00139254	XLOC_015702
ENSSSCT00000015027	ENSSSCG00000013753	TCONS_00139564	XLOC_015982
ENSSSCT00000068431	ENSSSCG00000042811	TCONS_00140436	XLOC_016650
ENSSSCT00000077218	ENSSSCG00000049610	TCONS_00141548	XLOC_015988
ENSSSCT00000074723	ENSSSCG00000044985	TCONS_00143693	XLOC_016664
ENSSSCT00000079484	ENSSSCG00000041220	TCONS_00143895	XLOC_016963
ENSSSCT00000089982	ENSSSCG00000033949	TCONS_00145051	XLOC_016962
ENSSSCT00000074099	ENSSSCG00000033949	TCONS_00145316	XLOC_015667
ENSSSCT00000038086	ENSSSCG00000033949	TCONS_00146344	XLOC_015844
ENSSSCT00000022324	ENSSSCG00000022757	TCONS_00147172	XLOC_015701
ENSSSCT00000047801	ENSSSCG00000039815	TCONS_00147299	XLOC_015964
ENSSSCT00000038404	ENSSSCG00000039815	TCONS_00150862	XLOC_016636

ENSSSCT00000086618	ENSSSCG00000048936	TCONS_00154296	XLOC_017510
ENSSSCT00000083747	ENSSSCG00000051369	TCONS_00155662	XLOC_018266
ENSSSCT00000072090	ENSSSCG00000043000	TCONS_00155663	XLOC_018270
ENSSSCT00000080883	ENSSSCG00000045478	TCONS_00156478	XLOC_017432
ENSSSCT00000071517	ENSSSCG00000048661	TCONS_00156480	XLOC_017432
ENSSSCT00000073286	ENSSSCG00000048661	TCONS_00156960	XLOC_017805
ENSSSCT00000056775	ENSSSCG00000035571	TCONS_00156994	XLOC_017835
ENSSSCT00000068842	ENSSSCG00000046723	TCONS_00158160	XLOC_017432
ENSSSCT00000084279	ENSSSCG00000043940	TCONS_00158582	XLOC_017846
ENSSSCT00000081564	ENSSSCG00000051426	TCONS_00158694	XLOC_017920
ENSSSCT00000069011	ENSSSCG00000045414	TCONS_00158827	XLOC_018030
ENSSSCT00000010559	ENSSSCG00000009638	TCONS_00159586	XLOC_017544
ENSSSCT00000010566	ENSSSCG00000009638	TCONS_00160211	XLOC_018383
ENSSSCT00000090395	ENSSSCG00000045477	TCONS_00160337	XLOC_018658
ENSSSCT00000075649	ENSSSCG00000045477	TCONS_00160517	XLOC_017432
ENSSSCT00000074962	ENSSSCG00000047090	TCONS_00160524	XLOC_017432
ENSSSCT00000071999	ENSSSCG00000043089	TCONS_00161054	XLOC_018104
ENSSSCT00000071772	ENSSSCG00000042705	TCONS_00162300	XLOC_017432
ENSSSCT00000078005	ENSSSCG00000048264	TCONS_00163187	XLOC_017479
ENSSSCT00000082010	ENSSSCG00000046622	TCONS_00164542	XLOC_017479
ENSSSCT00000057137	ENSSSCG00000040422	TCONS_00164895	XLOC_018195
ENSSSCT00000037405	ENSSSCG00000040413	TCONS_00165302	XLOC_017646
ENSSSCT00000088877	ENSSSCG00000040413	TCONS_00170166	XLOC_019797
ENSSSCT00000089426	ENSSSCG00000041866	TCONS_00170232	XLOC_019830
ENSSSCT00000086597	ENSSSCG00000048933	TCONS_00171978	XLOC_018761
ENSSSCT00000037611	ENSSSCG00000040740	TCONS_00172022	XLOC_018836
ENSSSCT00000076902	ENSSSCG00000040740	TCONS_00172954	XLOC_019836
ENSSSCT00000087772	ENSSSCG00000046495	TCONS_00173200	XLOC_018919
ENSSSCT00000081651	ENSSSCG00000050163	TCONS_00175442	XLOC_018697
ENSSSCT00000069804	ENSSSCG00000051194	TCONS_00176934	XLOC_019282
ENSSSCT00000075274	ENSSSCG00000050213	TCONS_00177724	XLOC_019830
ENSSSCT00000072596	ENSSSCG00000048101	TCONS_00181249	XLOC_020360
ENSSSCT00000083034	ENSSSCG00000044237	TCONS_00181269	XLOC_020379
ENSSSCT00000083486	ENSSSCG00000044237	TCONS_00181778	XLOC_020634
ENSSSCT00000068887	ENSSSCG00000050433	TCONS_00182021	XLOC_020737
ENSSSCT00000068138	ENSSSCG00000049992	TCONS_00182659	XLOC_021024
ENSSSCT00000075751	ENSSSCG00000041951	TCONS_00183039	XLOC_021247
ENSSSCT00000043324	ENSSSCG00000049635	TCONS_00183567	XLOC_021553
ENSSSCT00000070120	ENSSSCG00000045365	TCONS_00185087	XLOC_022569
ENSSSCT00000073363	ENSSSCG00000050824	TCONS_00185683	XLOC_020460
ENSSSCT00000070380	ENSSSCG00000042146	TCONS_00185931	XLOC_020627
ENSSSCT00000076793	ENSSSCG00000041274	TCONS_00185938	XLOC_020627
ENSSSCT00000074637	ENSSSCG00000045438	TCONS_00186110	XLOC_020742
ENSSSCT00000075426	ENSSSCG00000045849	TCONS_00186348	XLOC_020922

ENSSSCT00000072699	ENSSSCG00000044394	TCONS_00186785	XLOC_021226
ENSSSCT00000088330	ENSSSCG00000041958	TCONS_00186817	XLOC_021244
ENSSSCT00000076070	ENSSSCG00000044518	TCONS_00186819	XLOC_021245
ENSSSCT00000073589	ENSSSCG00000047818	TCONS_00186823	XLOC_021247
ENSSSCT00000076554	ENSSSCG00000045888	TCONS_00186826	XLOC_021249
ENSSSCT00000085448	ENSSSCG00000042176	TCONS_00187843	XLOC_022240
ENSSSCT00000080737	ENSSSCG00000048356	TCONS_00187924	XLOC_022301
ENSSSCT00000082269	ENSSSCG00000050752	TCONS_00187994	XLOC_022337
ENSSSCT00000077775	ENSSSCG00000015889	TCONS_00188139	XLOC_022572
ENSSSCT00000035160	ENSSSCG00000015889	TCONS_00188365	XLOC_020215
ENSSSCT00000091199	ENSSSCG00000046554	TCONS_00188938	XLOC_020742
ENSSSCT00000071812	ENSSSCG00000047236	TCONS_00189830	XLOC_021759
ENSSSCT00000070486	ENSSSCG00000048724	TCONS_00190027	XLOC_021992
ENSSSCT00000087812	ENSSSCG00000050974	TCONS_00190193	XLOC_022241
ENSSSCT00000078613	ENSSSCG00000048031	TCONS_00190279	XLOC_022308
ENSSSCT00000073779	ENSSSCG00000041385	TCONS_00190535	XLOC_020123
ENSSSCT00000090440	ENSSSCG00000048149	TCONS_00191062	XLOC_020741
ENSSSCT00000071737	ENSSSCG00000041092	TCONS_00191327	XLOC_021024
ENSSSCT00000077290	ENSSSCG00000048787	TCONS_00191418	XLOC_021204
ENSSSCT00000066755	ENSSSCG00000042914	TCONS_00191461	XLOC_021247
ENSSSCT00000017798	ENSSSCG00000016343	TCONS_00192088	XLOC_022250
ENSSSCT00000067776	ENSSSCG00000048785	TCONS_00193094	XLOC_021264
ENSSSCT00000087871	ENSSSCG00000044891	TCONS_00193133	XLOC_021342
ENSSSCT00000074331	ENSSSCG00000044891	TCONS_00194860	XLOC_021657
ENSSSCT00000066870	ENSSSCG00000047513	TCONS_00195342	XLOC_020123
ENSSSCT00000049491	ENSSSCG00000016379	TCONS_00196541	XLOC_022337
ENSSSCT00000059571	ENSSSCG00000016379	TCONS_00196936	XLOC_020738
ENSSSCT00000017834	ENSSSCG00000016379	TCONS_00196969	XLOC_020815
ENSSSCT00000057527	ENSSSCG00000016379	TCONS_00197879	XLOC_020262
ENSSSCT00000049527	ENSSSCG00000016379	TCONS_00202136	XLOC_020207
ENSSSCT00000084516	ENSSSCG00000042185	TCONS_00205595	XLOC_022768
ENSSSCT00000066537	ENSSSCG00000046669	TCONS_00205923	XLOC_022979
ENSSSCT00000070224	ENSSSCG00000046669	TCONS_00206238	XLOC_023099
ENSSSCT00000087939	ENSSSCG00000042439	TCONS_00206526	XLOC_023214
ENSSSCT00000090366	ENSSSCG00000041869	TCONS_00206527	XLOC_023214
ENSSSCT00000081389	ENSSSCG00000046332	TCONS_00206792	XLOC_023369
ENSSSCT00000084901	ENSSSCG00000049185	TCONS_00208672	XLOC_023053
ENSSSCT00000078574	ENSSSCG00000044109	TCONS_00208879	XLOC_023216
ENSSSCT00000087971	ENSSSCG00000042234	TCONS_00210522	XLOC_023214
ENSSSCT00000074472	ENSSSCG00000042234	TCONS_00212332	XLOC_023946
ENSSSCT00000083608	ENSSSCG00000041370	TCONS_00212528	XLOC_022896
ENSSSCT00000089944	ENSSSCG00000044939	TCONS_00212803	XLOC_023214
ENSSSCT00000077202	ENSSSCG00000044939	TCONS_00212805	XLOC_023214
ENSSSCT00000047639	ENSSSCG00000041267	TCONS_00214299	XLOC_022768

ENSSSCT00000079091	ENSSSCG00000041267	TCONS_00214926	XLOC_023689
ENSSSCT00000077039	ENSSSCG00000047709	TCONS_00221195	XLOC_024295
ENSSSCT00000075192	ENSSSCG00000049358	TCONS_00221409	XLOC_024420
ENSSSCT00000089910	ENSSSCG00000045889	TCONS_00221948	XLOC_024715
ENSSSCT00000077601	ENSSSCG00000051491	TCONS_00222006	XLOC_024743
ENSSSCT00000088640	ENSSSCG00000050772	TCONS_00222257	XLOC_024889
ENSSSCT00000072088	ENSSSCG00000050122	TCONS_00222391	XLOC_024971
ENSSSCT00000028661	ENSSSCG00000028322	TCONS_00222565	XLOC_025049
ENSSSCT00000068916	ENSSSCG00000049385	TCONS_00222585	XLOC_025059
ENSSSCT00000070118	ENSSSCG00000046054	TCONS_00222606	XLOC_025062
ENSSSCT00000067760	ENSSSCG00000046054	TCONS_00222687	XLOC_025097
ENSSSCT00000016697	ENSSSCG00000015324	TCONS_00222729	XLOC_025146
ENSSSCT00000073424	ENSSSCG00000015324	TCONS_00223092	XLOC_024508
ENSSSCT00000078028	ENSSSCG00000041560	TCONS_00223152	XLOC_024563
ENSSSCT00000076962	ENSSSCG00000045695	TCONS_00223352	XLOC_024770
ENSSSCT00000085781	ENSSSCG00000046599	TCONS_00223451	XLOC_024870
ENSSSCT00000078538	ENSSSCG00000048177	TCONS_00223797	XLOC_025146
ENSSSCT00000081878	ENSSSCG00000043982	TCONS_00223798	XLOC_025146
ENSSSCT00000090187	ENSSSCG00000048430	TCONS_00224075	XLOC_024508
ENSSSCT00000067348	ENSSSCG00000048430	TCONS_00224650	XLOC_025067
ENSSSCT00000069285	ENSSSCG00000049323	TCONS_00224890	XLOC_024483
ENSSSCT00000073982	ENSSSCG00000047638	TCONS_00225266	XLOC_024971
ENSSSCT00000069096	ENSSSCG00000046791	TCONS_00228221	XLOC_025150
ENSSSCT00000070504	ENSSSCG00000048806	TCONS_00229895	XLOC_024728
ENSSSCT00000057445	ENSSSCG00000034251	TCONS_00229911	XLOC_024728
ENSSSCT00000083177	ENSSSCG00000051354	TCONS_00229940	XLOC_024728
ENSSSCT00000089740	ENSSSCG00000049380	TCONS_00229941	XLOC_024728
ENSSSCT00000081885	ENSSSCG00000051296	TCONS_00229942	XLOC_024728
ENSSSCT00000066444	ENSSSCG00000041490	TCONS_00230743	XLOC_025344
ENSSSCT00000067059	ENSSSCG00000041490	TCONS_00230796	XLOC_025374
ENSSSCT00000081797	ENSSSCG00000044256	TCONS_00230797	XLOC_025374
ENSSSCT00000066781	ENSSSCG00000043129	TCONS_00230852	XLOC_025410
ENSSSCT00000075604	ENSSSCG00000043799	TCONS_00231197	XLOC_025626
ENSSSCT00000072709	ENSSSCG00000048118	TCONS_00231550	XLOC_025820
ENSSSCT00000088928	ENSSSCG00000045716	TCONS_00231551	XLOC_025820
ENSSSCT00000088592	ENSSSCG00000042491	TCONS_00231658	XLOC_025886
ENSSSCT00000066896	ENSSSCG00000050540	TCONS_00231894	XLOC_026048
ENSSSCT00000088858	ENSSSCG00000050540	TCONS_00231896	XLOC_026048
ENSSSCT00000071751	ENSSSCG00000044029	TCONS_00231897	XLOC_026048
ENSSSCT00000087006	ENSSSCG00000047296	TCONS_00232818	XLOC_025340
ENSSSCT00000078597	ENSSSCG00000042273	TCONS_00232819	XLOC_025340
ENSSSCT00000086401	ENSSSCG00000045723	TCONS_00232865	XLOC_025374
ENSSSCT00000091400	ENSSSCG00000047463	TCONS_00233091	XLOC_025586
ENSSSCT00000069636	ENSSSCG00000048789	TCONS_00233688	XLOC_026156

ENSSSCT00000087097	ENSSSCG00000039821	TCONS_00235308	XLOC_025374
ENSSSCT00000089869	ENSSSCG00000047440	TCONS_00235807	XLOC_026048
ENSSSCT00000072295	ENSSSCG00000043057	TCONS_00235808	XLOC_026048
ENSSSCT00000070020	ENSSSCG00000037652	TCONS_00235889	XLOC_026196
ENSSSCT00000076908	ENSSSCG00000041708	TCONS_00236247	XLOC_025406
ENSSSCT00000074302	ENSSSCG00000041121	TCONS_00236800	XLOC_026388
ENSSSCT00000085860	ENSSSCG00000049698	TCONS_00236808	XLOC_026399
ENSSSCT00000046823	ENSSSCG00000033217	TCONS_00236903	XLOC_026656
ENSSSCT00000085187	ENSSSCG00000041407	TCONS_00237117	XLOC_125668
ENSSSCT00000073163	ENSSSCG00000044261	TCONS_00237305	XLOC_026048
ENSSSCT00000070519	ENSSSCG00000049720	TCONS_00237307	XLOC_026048
ENSSSCT00000081556	ENSSSCG00000041587	TCONS_00237632	XLOC_125901
ENSSSCT00000075899	ENSSSCG00000050347	TCONS_00237980	XLOC_026048
ENSSSCT00000068101	ENSSSCG00000036379	TCONS_00238031	XLOC_026187
ENSSSCT00000053319	ENSSSCG00000036379	TCONS_00238315	XLOC_126166
ENSSSCT00000073632	ENSSSCG00000042386	TCONS_00238800	XLOC_126357
ENSSSCT00000087185	ENSSSCG00000045906	TCONS_00239187	XLOC_026041
ENSSSCT00000083679	ENSSSCG00000049077	TCONS_00239191	XLOC_026048
ENSSSCT00000082590	ENSSSCG00000050997	TCONS_00239198	XLOC_026048
ENSSSCT00000066967	ENSSSCG00000043494	TCONS_00239199	XLOC_026048
ENSSSCT00000074659	ENSSSCG00000043625	TCONS_00239843	XLOC_026341
ENSSSCT00000090577	ENSSSCG00000041682	TCONS_00240271	XLOC_026048
ENSSSCT00000049770	ENSSSCG00000036096	TCONS_00240333	XLOC_026157
ENSSSCT00000067259	ENSSSCG00000036096	TCONS_00240661	XLOC_025628
ENSSSCT00000077740	ENSSSCG00000036096	TCONS_00242867	XLOC_026728
ENSSSCT00000069967	ENSSSCG00000050063	TCONS_00242871	XLOC_026723
ENSSSCT00000080725	ENSSSCG00000050063	TCONS_00243030	XLOC_026779
ENSSSCT00000085647	ENSSSCG00000041715	TCONS_00243132	XLOC_026825
ENSSSCT00000083307	ENSSSCG00000041715	TCONS_00243171	XLOC_026859
ENSSSCT00000080047	ENSSSCG00000044588	TCONS_00243172	XLOC_026859
ENSSSCT00000074914	ENSSSCG00000048074	TCONS_00243176	XLOC_026859
ENSSSCT00000081341	ENSSSCG00000050566	TCONS_00243950	XLOC_027070
ENSSSCT00000086386	ENSSSCG00000050566	TCONS_00244087	XLOC_027150
ENSSSCT00000090965	ENSSSCG00000043219	TCONS_00244088	XLOC_027150
ENSSSCT00000083120	ENSSSCG00000051070	TCONS_00244195	XLOC_027177
ENSSSCT00000086402	ENSSSCG00000046619	TCONS_00244196	XLOC_027177
ENSSSCT00000074492	ENSSSCG00000038394	TCONS_00244197	XLOC_027177
ENSSSCT00000080343	ENSSSCG00000038394	TCONS_00244202	XLOC_027177
ENSSSCT00000089181	ENSSSCG00000049465	TCONS_00244203	XLOC_027177
ENSSSCT00000077768	ENSSSCG00000041317	TCONS_00244207	XLOC_027177
ENSSSCT00000068147	ENSSSCG00000042714	TCONS_00244208	XLOC_027177
ENSSSCT00000075710	ENSSSCG00000049122	TCONS_00244209	XLOC_027177
ENSSSCT00000066779	ENSSSCG00000051523	TCONS_00244219	XLOC_027177
ENSSSCT00000084355	ENSSSCG00000048711	TCONS_00244220	XLOC_027177

ENSSSCT00000071991	ENSSSCG00000040267	TCONS_00244360	XLOC_027177
ENSSSCT00000089899	ENSSSCG00000040267	TCONS_00244454	XLOC_027177
ENSSSCT00000066924	ENSSSCG00000048192	TCONS_00244489	XLOC_027150
ENSSSCT00000090865	ENSSSCG00000036880	TCONS_00244536	XLOC_027177
ENSSSCT00000064274	ENSSSCG00000036880	TCONS_00244580	XLOC_027150
ENSSSCT00000078212	ENSSSCG00000036880	TCONS_00245003	XLOC_027163
ENSSSCT00000069248	ENSSSCG00000036880	TCONS_00245167	XLOC_027210
ENSSSCT00000083321	ENSSSCG00000036880	TCONS_00245175	XLOC_027205
ENSSSCT00000084502	ENSSSCG00000036880	TCONS_00246237	XLOC_027539
ENSSSCT00000046804	ENSSSCG00000040582	TCONS_00246287	XLOC_027559
ENSSSCT00000090068	ENSSSCG00000050178	TCONS_00246321	XLOC_027565
ENSSSCT00000091525	ENSSSCG00000046806	TCONS_00246323	XLOC_027565
ENSSSCT00000037621	ENSSSCG00000032483	TCONS_00246908	XLOC_027533
ENSSSCT00000070837	ENSSSCG00000046817	TCONS_00247099	XLOC_027559
ENSSSCT00000072287	ENSSSCG00000047210	TCONS_00247437	XLOC_125594
ENSSSCT00000076990	ENSSSCG00000046686	TCONS_00247782	XLOC_125890
ENSSSCT00000073015	ENSSSCG00000051090	TCONS_00248523	XLOC_028289
ENSSSCT00000080719	ENSSSCG00000049098	TCONS_00250327	XLOC_028336
ENSSSCT00000081789	ENSSSCG00000042798	TCONS_00251356	XLOC_028458
ENSSSCT00000068397	ENSSSCG00000048678	TCONS_00251422	XLOC_027699
ENSSSCT00000072262	ENSSSCG00000048678	TCONS_00253727	XLOC_125593
ENSSSCT00000069975	ENSSSCG00000046151	TCONS_00253962	XLOC_125809
ENSSSCT00000075880	ENSSSCG00000046151	TCONS_00254464	XLOC_126357
ENSSSCT00000077704	ENSSSCG00000046151	TCONS_00254772	XLOC_028730
ENSSSCT00000075650	ENSSSCG00000042468	TCONS_00260942	XLOC_125668
ENSSSCT00000087261	ENSSSCG00000050230	TCONS_00272645	XLOC_126113
ENSSSCT00000071286	ENSSSCG00000047133	TCONS_00272797	XLOC_126352
ENSSSCT00000087691	ENSSSCG00000045865	TCONS_00277252	XLOC_126166
ENSSSCT00000067639	ENSSSCG00000042179	TCONS_00377373	XLOC_196963
ENSSSCT00000067080	ENSSSCG00000046247	TCONS_00379975	XLOC_198053
ENSSSCT00000088112	ENSSSCG00000051779	TCONS_00381169	XLOC_198536
ENSSSCT00000073756	ENSSSCG00000048719	TCONS_00387643	XLOC_222011
ENSSSCT00000078854	ENSSSCG00000044016	TCONS_00392519	XLOC_197252
ENSSSCT00000079402	ENSSSCG00000044016	TCONS_00392836	XLOC_197519
ENSSSCT00000084987	ENSSSCG00000044016	TCONS_00393367	XLOC_198088
ENSSSCT00000075773	ENSSSCG00000048499	TCONS_00393375	XLOC_198088
ENSSSCT00000070078	ENSSSCG00000043319	TCONS_00401852	XLOC_197162
ENSSSCT00000090712	ENSSSCG00000051418	TCONS_00402182	XLOC_197518
ENSSSCT00000074431	ENSSSCG00000047157	TCONS_00402630	XLOC_198116
ENSSSCT00000076275	ENSSSCG00000047157	TCONS_00402699	XLOC_198162
ENSSSCT00000072509	ENSSSCG00000042062	TCONS_00402861	XLOC_198314
ENSSSCT00000067062	ENSSSCG00000042114	TCONS_00410373	XLOC_197162
ENSSSCT00000081814	ENSSSCG00000044662	TCONS_00410725	XLOC_197834
ENSSSCT00000046000	ENSSSCG00000006581	TCONS_00417445	XLOC_197008

ENSSSCT00000036447	ENSSSCG00000006581	TCONS_00424817	XLOC_197008
ENSSSCT00000074087	ENSSSCG00000051301	TCONS_00424818	XLOC_197008
ENSSSCT00000067154	ENSSSCG00000043747	TCONS_00425419	XLOC_198042
ENSSSCT00000090804	ENSSSCG00000041824	TCONS_00438326	XLOC_197989
ENSSSCT00000084405	ENSSSCG00000040282	TCONS_00443601	XLOC_196877
ENSSSCT00000067900	ENSSSCG00000042103	TCONS_00443828	XLOC_197252
ENSSSCT00000086271	ENSSSCG00000042103	TCONS_00444825	XLOC_200348
ENSSSCT00000075632	ENSSSCG00000042741	TCONS_00448579	XLOC_197044
ENSSSCT00000087410	ENSSSCG00000042741	TCONS_00485453	XLOC_244231
ENSSSCT00000077813	ENSSSCG00000049063	TCONS_00485701	XLOC_244372
ENSSSCT00000074954	ENSSSCG00000042541	TCONS_00486583	XLOC_244815
ENSSSCT00000072238	ENSSSCG00000050434	TCONS_00549117	XLOC_244773
ENSSSCT00000070433	ENSSSCG00000050434	TCONS_00593310	XLOC_244004
ENSSSCT00000079495	ENSSSCG00000042787	TCONS_00600334	XLOC_244797
ENSSSCT00000072772	ENSSSCG00000042787	TCONS_00641650	XLOC_243806
ENSSSCT00000090916	ENSSSCG00000044694	TCONS_00660965	XLOC_336058
ENSSSCT00000082508	ENSSSCG00000045831	TCONS_00662972	XLOC_336944
ENSSSCT00000090834	ENSSSCG00000048462	TCONS_00665478	XLOC_338607
ENSSSCT00000072961	ENSSSCG00000044011	TCONS_00675969	XLOC_394182
ENSSSCT00000076581	ENSSSCG00000044011	TCONS_00678723	XLOC_335554
ENSSSCT00000089124	ENSSSCG00000043770	TCONS_00680094	XLOC_337115
ENSSSCT00000082498	ENSSSCG00000042641	TCONS_00680183	XLOC_337383
ENSSSCT00000084086	ENSSSCG00000045387	TCONS_00690482	XLOC_336058
ENSSSCT00000082660	ENSSSCG00000047910	TCONS_00691123	XLOC_336944
ENSSSCT00000089613	ENSSSCG00000043604	TCONS_00730252	XLOC_335592
ENSSSCT00000073575	ENSSSCG00000047186	TCONS_00738982	XLOC_335785
ENSSSCT00000036268	ENSSSCG00000028695	TCONS_00748274	XLOC_335554
ENSSSCT00000013696	ENSSSCG00000012527	TCONS_00762145	XLOC_335607
ENSSSCT00000080226	ENSSSCG00000041483	TCONS_00801077	XLOC_406823
ENSSSCT00000077136	ENSSSCG00000044160	TCONS_00804582	XLOC_408409
ENSSSCT00000072277	ENSSSCG00000041717	TCONS_00817555	XLOC_407696
ENSSSCT00000083771	ENSSSCG00000041717	TCONS_00818006	XLOC_408080
ENSSSCT00000069044	ENSSSCG00000041717	TCONS_00826103	XLOC_407048
ENSSSCT00000082512	ENSSSCG00000041717	TCONS_00826340	XLOC_407406
ENSSSCT00000072884	ENSSSCG00000049494	TCONS_00826375	XLOC_407450
ENSSSCT00000086001	ENSSSCG00000050144	TCONS_00826909	XLOC_408080
ENSSSCT00000077576	ENSSSCG00000049849	TCONS_00827166	XLOC_408951
ENSSSCT00000074247	ENSSSCG00000045404	TCONS_00888108	XLOC_408092
ENSSSCT00000075388	ENSSSCG00000048330	TCONS_00922943	XLOC_469598
ENSSSCT00000068800	ENSSSCG00000043173	TCONS_00946121	XLOC_470257
ENSSSCT00000067332	ENSSSCG00000043173	TCONS_00953765	XLOC_469719
ENSSSCT00000013877	ENSSSCG00000042098	TCONS_00986305	XLOC_504010
ENSSSCT00000085192	ENSSSCG00000042098	TCONS_00986361	XLOC_504041
ENSSSCT00000083598	ENSSSCG00000042098	TCONS_00987036	XLOC_504322

ENSSSCT00000044246	ENSSSCG00000031801	TCONS_00987037	XLOC_504322
ENSSSCT00000048121	ENSSSCG00000031801	TCONS_00994973	XLOC_535646
ENSSSCT00000077720	ENSSSCG00000043554	TCONS_00995537	XLOC_537632
ENSSSCT00000088443	ENSSSCG00000046331	TCONS_00996369	XLOC_504175
ENSSSCT00000055372	ENSSSCG00000037520	TCONS_01002748	XLOC_505378
ENSSSCT00000088428	ENSSSCG00000050795	TCONS_01011275	XLOC_521952
ENSSSCT00000069865	ENSSSCG00000044527	TCONS_01013804	XLOC_504141
ENSSSCT00000082392	ENSSSCG00000050158	TCONS_01018894	XLOC_504106
ENSSSCT00000069012	ENSSSCG00000044922	TCONS_01044196	XLOC_504687
ENSSSCT00000074722	ENSSSCG00000044922	TCONS_01060471	XLOC_540223
ENSSSCT00000085317	ENSSSCG00000047516	TCONS_01066688	XLOC_565805
ENSSSCT00000080920	ENSSSCG00000050204	TCONS_01068235	XLOC_540256
ENSSSCT00000079661	ENSSSCG00000041566	TCONS_01073095	XLOC_539762
ENSSSCT00000076164	ENSSSCG00000044908	TCONS_01073599	XLOC_540362
ENSSSCT00000084963	ENSSSCG00000041400	TCONS_01078704	XLOC_540332
ENSSSCT00000069210	ENSSSCG00000045495	TCONS_01087313	XLOC_540073
ENSSSCT00000089857	ENSSSCG00000047706	TCONS_01104375	XLOC_539767
ENSSSCT00000085391	ENSSSCG00000045069	TCONS_01121036	XLOC_570248
ENSSSCT00000089195	ENSSSCG00000049534	TCONS_01126194	XLOC_572358
ENSSSCT00000074989	ENSSSCG00000041508	TCONS_01129558	XLOC_574152
ENSSSCT00000085926	ENSSSCG00000041434	TCONS_01145444	XLOC_570364
ENSSSCT00000079411	ENSSSCG00000041410	TCONS_01160321	XLOC_570746
ENSSSCT00000091606	ENSSSCG00000041410	TCONS_01174756	XLOC_570303
ENSSSCT00000072786	ENSSSCG00000051432	TCONS_01176148	XLOC_572507
ENSSSCT00000091407	ENSSSCG00000044205	TCONS_01198652	XLOC_570248
ENSSSCT00000075350	ENSSSCG00000045946	TCONS_01198661	XLOC_570259
ENSSSCT00000001826	ENSSSCG00000026741	TCONS_01199681	XLOC_572239
ENSSSCT00000072418	ENSSSCG00000042003	TCONS_01220358	XLOC_571628
ENSSSCT00000090141	ENSSSCG00000047853	TCONS_01230505	XLOC_570259
ENSSSCT00000075156	ENSSSCG00000042487	TCONS_01230824	XLOC_570897
ENSSSCT00000089735	ENSSSCG00000043650	TCONS_01247769	XLOC_570956
ENSSSCT00000082554	ENSSSCG00000050687	TCONS_01248875	XLOC_574267
ENSSSCT00000080936	ENSSSCG00000050687	TCONS_01293818	XLOC_653073
ENSSSCT00000081970	ENSSSCG00000050687	TCONS_01295672	XLOC_653742
ENSSSCT00000089316	ENSSSCG00000050687	TCONS_01311030	XLOC_707729
ENSSSCT00000091094	ENSSSCG00000045730	TCONS_01314699	XLOC_652927
ENSSSCT00000075191	ENSSSCG00000048983	TCONS_01315146	XLOC_653220
ENSSSCT00000087190	ENSSSCG00000047067	TCONS_01320819	XLOC_676896
ENSSSCT00000088863	ENSSSCG00000049161	TCONS_01329228	XLOC_653748
ENSSSCT00000002642	ENSSSCG00000050616	TCONS_01329688	XLOC_654288
ENSSSCT00000078864	ENSSSCG00000050616	TCONS_01342306	XLOC_653238
ENSSSCT00000078294	ENSSSCG00000048500	TCONS_01353930	XLOC_653374
ENSSSCT00000050073	ENSSSCG00000049596	TCONS_01364778	XLOC_653128
ENSSSCT00000091115	ENSSSCG00000049596	TCONS_01364955	XLOC_653352

ENSSSCT00000085149	ENSSSCG00000047625	TCONS_01378076	XLOC_680332
ENSSSCT00000077598	ENSSSCG00000045310	TCONS_01410031	XLOC_654702
ENSSSCT00000022745	ENSSSCG00000021146	TCONS_01455400	XLOC_730253
ENSSSCT00000067919	ENSSSCG00000051016	TCONS_01471736	XLOC_730210
ENSSSCT00000089312	ENSSSCG00000047048	TCONS_01481593	XLOC_728800
ENSSSCT00000085551	ENSSSCG00000042615	TCONS_01482609	XLOC_730210
ENSSSCT00000089330	ENSSSCG00000048556	TCONS_01494064	XLOC_728787
ENSSSCT00000067125	ENSSSCG00000048556	TCONS_01495343	XLOC_731466
ENSSSCT00000046651	ENSSSCG00000051274	TCONS_01553843	XLOC_728787
ENSSSCT00000074083	ENSSSCG00000047414	TCONS_01554441	XLOC_730195
ENSSSCT00000087631	ENSSSCG00000035352	TCONS_01592997	XLOC_799347
ENSSSCT00000078931	ENSSSCG00000042568	TCONS_01593406	XLOC_799496
ENSSSCT00000082732	ENSSSCG00000046072	TCONS_01609566	XLOC_799240
ENSSSCT00000068789	ENSSSCG00000046072	TCONS_01610160	XLOC_799879
ENSSSCT00000079328	ENSSSCG00000041214	TCONS_01620726	XLOC_800274
ENSSSCT00000089374	ENSSSCG00000051360	TCONS_01621135	XLOC_801070
ENSSSCT00000067469	ENSSSCG00000051710	TCONS_01631229	XLOC_799638
ENSSSCT00000066632	ENSSSCG00000042788	TCONS_01650214	XLOC_800414
ENSSSCT00000068213	ENSSSCG00000042788	TCONS_01665407	XLOC_800534
ENSSSCT00000079593	ENSSSCG00000044454	TCONS_01721700	XLOC_862572
ENSSSCT00000082438	ENSSSCG00000043669	TCONS_01722610	XLOC_862837
ENSSSCT00000066874	ENSSSCG00000042272	TCONS_01723915	XLOC_863302
ENSSSCT00000088196	ENSSSCG00000049407	TCONS_01724083	XLOC_863333
ENSSSCT00000091501	ENSSSCG00000045128	TCONS_01724189	XLOC_863330
ENSSSCT00000089262	ENSSSCG00000044335	TCONS_01725064	XLOC_863710
ENSSSCT00000069188	ENSSSCG00000050692	TCONS_01725791	XLOC_864016
ENSSSCT00000082261	ENSSSCG00000045667	TCONS_01725903	XLOC_864055
ENSSSCT00000073202	ENSSSCG00000050137	TCONS_01753842	XLOC_864682
ENSSSCT00000082384	ENSSSCG00000044141	TCONS_01769728	XLOC_862244
ENSSSCT00000069597	ENSSSCG00000050339	TCONS_01770904	XLOC_863330
ENSSSCT00000083921	ENSSSCG00000043418	TCONS_01770908	XLOC_863330
ENSSSCT00000069088	ENSSSCG00000042692	TCONS_01790695	XLOC_864534
ENSSSCT00000074468	ENSSSCG00000047986	TCONS_01804704	XLOC_862244
ENSSSCT00000073030	ENSSSCG00000041180	TCONS_01819770	XLOC_862176
ENSSSCT00000081483	ENSSSCG00000045402	TCONS_01832439	XLOC_863046
ENSSSCT00000078333	ENSSSCG00000044736	TCONS_01834053	XLOC_866814
ENSSSCT00000068804	ENSSSCG00000048060	TCONS_01846548	XLOC_863972
ENSSSCT00000066953	ENSSSCG00000049042	TCONS_01868478	XLOC_862886
ENSSSCT00000072250	ENSSSCG00000051151	TCONS_01892626	XLOC_862548
ENSSSCT00000074751	ENSSSCG00000051151	TCONS_01938557	XLOC_962631
ENSSSCT00000072182	ENSSSCG00000047972	TCONS_01943964	XLOC_965153
ENSSSCT00000041878	ENSSSCG00000039883	TCONS_01957779	XLOC_962514
ENSSSCT00000088953	ENSSSCG00000045534	TCONS_01959340	XLOC_964085
ENSSSCT00000079538	ENSSSCG00000043931	TCONS_01959863	XLOC_965573

ENSSSCT00000090673	ENSSSCG00000046389	TCONS_01970074	XLOC_963181
ENSSSCT00000070949	ENSSSCG00000050385	TCONS_01970899	XLOC_964449
ENSSSCT00000067914	ENSSSCG00000050382	TCONS_01982328	XLOC_962534
ENSSSCT00000078982	ENSSSCG00000047338	TCONS_01982614	XLOC_962948
ENSSSCT00000077492	ENSSSCG00000049125	TCONS_01982743	XLOC_963141
ENSSSCT00000068145	ENSSSCG00000043900	TCONS_01983233	XLOC_963908
ENSSSCT00000075195	ENSSSCG00000043013	TCONS_02002930	XLOC_962863
ENSSSCT00000090799	ENSSSCG00000046743	TCONS_02003462	XLOC_963912
ENSSSCT00000077102	ENSSSCG00000045895	TCONS_02011718	XLOC_964978
ENSSSCT00000049222	ENSSSCG00000041341	TCONS_02082236	XLOC_1032494
ENSSSCT00000074957	ENSSSCG00000041341	TCONS_02083387	XLOC_1033021
ENSSSCT00000066708	ENSSSCG00000046352	TCONS_02104769	XLOC_1032150
ENSSSCT00000081677	ENSSSCG00000041994	TCONS_02104943	XLOC_1032405
ENSSSCT00000084346	ENSSSCG00000047250	TCONS_02105054	XLOC_1032494
ENSSSCT00000081995	ENSSSCG00000045445	TCONS_02115074	XLOC_1032027
ENSSSCT00000090963	ENSSSCG00000050550	TCONS_02123582	XLOC_1032494
ENSSSCT00000087162	ENSSSCG00000041012	TCONS_02123794	XLOC_1032885
ENSSSCT00000074562	ENSSSCG00000045152	TCONS_02131757	XLOC_1033049
ENSSSCT00000077835	ENSSSCG00000047793	TCONS_02144661	XLOC_1031951
ENSSSCT00000080028	ENSSSCG00000045154	TCONS_02145194	XLOC_1033050
ENSSSCT00000081802	ENSSSCG00000045154	TCONS_02152702	XLOC_1032494
ENSSSCT00000088651	ENSSSCG00000050664	TCONS_02196594	XLOC_1093415
ENSSSCT00000069321	ENSSSCG00000044618	TCONS_02198377	XLOC_1094145
ENSSSCT00000067351	ENSSSCG00000049805	TCONS_02199343	XLOC_1094570
ENSSSCT00000071360	ENSSSCG00000046444	TCONS_02205514	XLOC_1113213
ENSSSCT00000066604	ENSSSCG00000047944	TCONS_02214328	XLOC_1093322
ENSSSCT00000054610	ENSSSCG00000037997	TCONS_02215007	XLOC_1094023
ENSSSCT00000082437	ENSSSCG00000041187	TCONS_02215055	XLOC_1094090
ENSSSCT00000087852	ENSSSCG00000046945	TCONS_02215177	XLOC_1094172
ENSSSCT00000069001	ENSSSCG00000042888	TCONS_02215587	XLOC_1094679
ENSSSCT00000079176	ENSSSCG00000046868	TCONS_02225799	XLOC_1094145
ENSSSCT00000069875	ENSSSCG00000048325	TCONS_02237649	XLOC_1094595
ENSSSCT00000037412	ENSSSCG00000031683	TCONS_02247192	XLOC_1093981
ENSSSCT00000073978	ENSSSCG00000031683	TCONS_02247215	XLOC_1093993
ENSSSCT00000085962	ENSSSCG00000047204	TCONS_02247610	XLOC_1094677
ENSSSCT00000066501	ENSSSCG00000044397	TCONS_02256937	XLOC_1094647
ENSSSCT00000071695	ENSSSCG00000043552	TCONS_02263851	XLOC_1093161
ENSSSCT00000073620	ENSSSCG00000045657	TCONS_02264268	XLOC_1094090
ENSSSCT00000087611	ENSSSCG00000050251	TCONS_02270253	XLOC_1147720
ENSSSCT00000088051	ENSSSCG00000051266	TCONS_02271969	XLOC_1093601
ENSSSCT00000086268	ENSSSCG00000049283	TCONS_02286347	XLOC_1095352
ENSSSCT00000081330	ENSSSCG00000049283	TCONS_02333413	XLOC_1163206
ENSSSCT00000082884	ENSSSCG00000046263	TCONS_02364308	XLOC_1178942
ENSSSCT00000079425	ENSSSCG00000045098	TCONS_02378604	XLOC_1218634

ENSSSCT00000075031	ENSSSCG00000048861
ENSSSCT00000066646	ENSSSCG00000045417
ENSSSCT00000084955	ENSSSCG00000045440
ENSSSCT00000081694	ENSSSCG00000043907
ENSSSCT00000077031	ENSSSCG00000049828
ENSSSCT00000051403	ENSSSCG00000031414
ENSSSCT00000069592	ENSSSCG00000042043
ENSSSCT00000070271	ENSSSCG00000038429
ENSSSCT00000070569	ENSSSCG00000038429
ENSSSCT00000082140	ENSSSCG00000041820
ENSSSCT00000087572	ENSSSCG00000050830
ENSSSCT00000077749	ENSSSCG00000051557
ENSSSCT00000075418	ENSSSCG00000037324
ENSSSCT00000081323	ENSSSCG00000037324
ENSSSCT00000085804	ENSSSCG00000044004
ENSSSCT00000069671	ENSSSCG00000047985
ENSSSCT00000082329	ENSSSCG00000048082
ENSSSCT00000079784	ENSSSCG00000048082
ENSSSCT00000008000	ENSSSCG00000007311
ENSSSCT00000090878	ENSSSCG00000045086
ENSSSCT00000083036	ENSSSCG00000045086
ENSSSCT00000088584	ENSSSCG00000045287
ENSSSCT00000075007	ENSSSCG00000051590
ENSSSCT00000090112	ENSSSCG00000046624
ENSSSCT00000075432	ENSSSCG00000044986
ENSSSCT00000090197	ENSSSCG00000048041
ENSSSCT00000090820	ENSSSCG00000047757
ENSSSCT00000069708	ENSSSCG00000042682
ENSSSCT00000091358	ENSSSCG00000033537
ENSSSCT00000071780	ENSSSCG00000033537
ENSSSCT00000036938	ENSSSCG00000033537
ENSSSCT00000066689	ENSSSCG00000033537
ENSSSCT00000071659	ENSSSCG00000046608
ENSSSCT00000086302	ENSSSCG00000050015
ENSSSCT00000073209	ENSSSCG00000050015
ENSSSCT00000088232	ENSSSCG00000050015
ENSSSCT00000066997	ENSSSCG00000051615
ENSSSCT00000070888	ENSSSCG00000051788
ENSSSCT00000075041	ENSSSCG00000049532
ENSSSCT00000074255	ENSSSCG00000051629
ENSSSCT00000078695	ENSSSCG00000050463
ENSSSCT00000079949	ENSSSCG00000051405
ENSSSCT00000085766	ENSSSCG00000043360
ENSSSCT00000077796	ENSSSCG00000050447

ENSSSCT00000026445	ENSSSCG00000024743
ENSSSCT00000084558	ENSSSCG00000041588
ENSSSCT00000090529	ENSSSCG00000050184
ENSSSCT00000078505	ENSSSCG00000050184
ENSSSCT00000079980	ENSSSCG00000051634
ENSSSCT00000066678	ENSSSCG00000041793
ENSSSCT00000072963	ENSSSCG00000046684
ENSSSCT00000080400	ENSSSCG00000044492
ENSSSCT00000083362	ENSSSCG00000046841
ENSSSCT00000078842	ENSSSCG00000048019
ENSSSCT00000083560	ENSSSCG00000041710
ENSSSCT00000084261	ENSSSCG00000049865
ENSSSCT00000089383	ENSSSCG00000047466
ENSSSCT00000075300	ENSSSCG00000047466
ENSSSCT00000089585	ENSSSCG00000047466
ENSSSCT00000080136	ENSSSCG00000047466
ENSSSCT00000073899	ENSSSCG00000046461
ENSSSCT00000087125	ENSSSCG00000046461
ENSSSCT00000079032	ENSSSCG00000051298
ENSSSCT00000087681	ENSSSCG00000048095
ENSSSCT00000067579	ENSSSCG00000033707
ENSSSCT00000088761	ENSSSCG00000044134
ENSSSCT00000082336	ENSSSCG00000046607
ENSSSCT00000082666	ENSSSCG00000047041
ENSSSCT00000079737	ENSSSCG00000043139
ENSSSCT00000084466	ENSSSCG00000049100
ENSSSCT00000079415	ENSSSCG00000042618
ENSSSCT00000081733	ENSSSCG00000047771
ENSSSCT00000077940	ENSSSCG00000046026
ENSSSCT00000070250	ENSSSCG00000042667
ENSSSCT00000076112	ENSSSCG00000043869
ENSSSCT00000073433	ENSSSCG00000051193
ENSSSCT00000087593	ENSSSCG00000049481
ENSSSCT00000084379	ENSSSCG00000045735
ENSSSCT00000089760	ENSSSCG00000045735
ENSSSCT00000090430	ENSSSCG00000051552
ENSSSCT00000069507	ENSSSCG00000047942
ENSSSCT00000075312	ENSSSCG00000041685
ENSSSCT00000088608	ENSSSCG00000051336
ENSSSCT00000088553	ENSSSCG00000051650
ENSSSCT00000071625	ENSSSCG00000045485
ENSSSCT00000077026	ENSSSCG00000048754
ENSSSCT00000083205	ENSSSCG00000034024
ENSSSCT00000089846	ENSSSCG00000034024

ENSSSCT00000090407	ENSSSCG00000049640
ENSSSCT00000074499	ENSSSCG00000046512
ENSSSCT00000072985	ENSSSCG00000046512
ENSSSCT00000066893	ENSSSCG00000048433
ENSSSCT00000085290	ENSSSCG00000026140
ENSSSCT00000031425	ENSSSCG00000026140
ENSSSCT00000087167	ENSSSCG00000047081
ENSSSCT00000091510	ENSSSCG00000047843
ENSSSCT00000084160	ENSSSCG00000048420
ENSSSCT00000066453	ENSSSCG00000045378
ENSSSCT00000090314	ENSSSCG00000041231
ENSSSCT00000082659	ENSSSCG00000041985
ENSSSCT00000075005	ENSSSCG00000041985
ENSSSCT00000077602	ENSSSCG00000045717
ENSSSCT00000086154	ENSSSCG00000043190
ENSSSCT00000078252	ENSSSCG00000048905
ENSSSCT00000089447	ENSSSCG00000041163
ENSSSCT00000079382	ENSSSCG00000049417
ENSSSCT00000067335	ENSSSCG00000041875
ENSSSCT00000067299	ENSSSCG00000041461
ENSSSCT00000085131	ENSSSCG00000041461
ENSSSCT00000075068	ENSSSCG00000041461
ENSSSCT00000079903	ENSSSCG00000041461
ENSSSCT00000081099	ENSSSCG00000045913
ENSSSCT00000074118	ENSSSCG00000048204
ENSSSCT00000068216	ENSSSCG00000033469
ENSSSCT00000055350	ENSSSCG00000033469
ENSSSCT00000079890	ENSSSCG00000045433
ENSSSCT00000064970	ENSSSCG00000034215
ENSSSCT00000050236	ENSSSCG00000039672
ENSSSCT00000083151	ENSSSCG00000041596
ENSSSCT00000077712	ENSSSCG00000041596

Table S3 The detailed information of DE lncRNAs identified in this study

Time comparison	DE LncRNAs	logFC	*P*-value	Adjusted *P*-value
(-10)vs-14	XLOC_027177	0.88	1.49E-12	2.22E-09
	ENSSSCG00000050649	1.23	6.51E-12	8.81E-09
	XLOC_025150	1.02	9.05E-08	3.55E-05
	ENSSSCG00000024743	-0.66	2.94E-07	9.44E-05
	XLOC_026156	0.82	3.76E-06	8.50E-04
	ENSSSCG00000039672	1.34	1.15E-05	2.23E-03
	XLOC_337383	-0.77	2.33E-04	2.60E-02
	XLOC_025146	0.58	2.80E-04	2.98E-02
	ENSSSCG00000046247	1.02	5.37E-04	4.78E-02
(-6)vs-14	XLOC_025150	1.69	7.98E-18	1.91E-15
	ENSSSCG00000050649	1.24	1.05E-13	1.60E-11
	XLOC_026156	1.40	2.55E-13	3.63E-11
	XLOC_027533	0.71	9.65E-13	1.26E-10
	XLOC_012336	0.70	9.02E-09	6.34E-07
	XLOC_026048	1.62	1.99E-08	1.31E-06
	ENSSSCG00000048149	-1.23	1.85E-07	1.00E-05
	ENSSSCG00000024743	-0.61	2.43E-07	1.25E-05
	XLOC_408080	1.18	3.57E-07	1.76E-05
	XLOC_004739	0.56	5.42E-07	2.52E-05
	ENSSSCG00000046743	1.32	6.50E-07	2.99E-05
	ENSSSCG00000047250	-0.62	8.25E-07	3.71E-05
	ENSSSCG00000036096	0.97	1.94E-06	7.71E-05
	XLOC_799496	-0.84	2.15E-06	8.45E-05
	ENSSSCG00000041987	0.68	1.30E-05	4.16E-04
	XLOC_025146	0.78	1.35E-05	4.29E-04
	ENSSSCG00000049480	0.61	2.44E-05	7.11E-04
	ENSSSCG00000041508	0.85	4.38E-05	1.16E-03
	ENSSSCG00000049323	-0.76	4.44E-05	1.17E-03
	ENSSSCG00000047361	0.74	6.27E-05	1.56E-03
	ENSSSCG00000051023	-0.59	6.43E-05	1.59E-03
	XLOC_009655	0.42	6.93E-05	1.69E-03
	ENSSSCG00000044256	-0.76	9.47E-05	2.19E-03
	XLOC_570364	1.11	1.55E-04	3.24E-03
	XLOC_024743	0.85	1.78E-04	3.63E-03
	ENSSSCG00000051523	0.73	1.97E-04	3.95E-03
	ENSSSCG00000045200	-0.38	3.28E-04	5.89E-03
	XLOC_014132	0.95	3.35E-04	5.98E-03

ENSSSCG00000006581	-0.48	3.51E-04	6.20E-03
XLOC_026859	-0.45	4.01E-04	6.90E-03
ENSSSCG00000033794	-0.66	4.92E-04	8.08E-03
XLOC_009194	0.35	5.27E-04	8.54E-03
XLOC_021992	0.72	5.47E-04	8.84E-03
XLOC_504106	0.57	7.54E-04	1.13E-02
ENSSSCG00000046841	0.65	7.67E-04	1.15E-02
XLOC_012374	0.81	7.71E-04	1.15E-02
ENSSSCG00000040582	-0.61	7.85E-04	1.17E-02
ENSSSCG00000046247	1.33	9.40E-04	1.33E-02
ENSSSCG00000047513	0.52	9.58E-04	1.35E-02
XLOC_009020	0.69	1.11E-03	1.52E-02
XLOC_015687	0.64	1.25E-03	1.67E-02
ENSSSCG00000048060	0.86	1.31E-03	1.73E-02
ENSSSCG00000039672	0.80	1.34E-03	1.76E-02
XLOC_005878	-0.52	1.47E-03	1.90E-02
ENSSSCG00000042682	-0.36	1.77E-03	2.17E-02
XLOC_021553	-0.59	1.82E-03	2.21E-02
XLOC_337383	-0.53	1.89E-03	2.28E-02
ENSSSCG00000045831	0.64	1.92E-03	2.31E-02
ENSSSCG00000044764	0.51	2.01E-03	2.38E-02
ENSSSCG00000043175	-0.39	2.13E-03	2.48E-02
XLOC_244231	-1.04	2.18E-03	2.52E-02
ENSSSCG00000048405	1.12	2.21E-03	2.55E-02
ENSSSCG00000040422	-0.66	2.21E-03	2.55E-02
XLOC_654288	-0.89	2.23E-03	2.57E-02
ENSSSCG00000048031	0.79	2.27E-03	2.60E-02
ENSSSCG00000046444	1.37	2.58E-03	2.87E-02
ENSSSCG00000046607	-0.55	2.79E-03	3.04E-02
ENSSSCG00000012527	-0.70	2.91E-03	3.14E-02
ENSSSCG00000046599	-0.69	3.01E-03	3.20E-02
ENSSSCG00000050015	-0.26	3.20E-03	3.35E-02
ENSSSCG00000045128	-0.53	3.23E-03	3.37E-02
ENSSSCG00000043982	0.83	3.25E-03	3.38E-02
ENSSSCG00000042185	0.48	3.47E-03	3.53E-02
ENSSSCG00000045895	-0.62	3.81E-03	3.77E-02
ENSSSCG00000051650	-1.50	3.81E-03	3.77E-02
XLOC_198536	-0.58	4.76E-03	4.46E-02
XLOC_005792	0.48	4.83E-03	4.51E-02
XLOC_010030	0.59	4.90E-03	4.56E-02
ENSSSCG00000041560	1.23	5.08E-03	4.68E-02
ENSSSCG00000046945	-0.29	5.16E-03	4.73E-02

	ENSSSCG00000042663	-0.33	5.46E-03	4.94E-02
	XLOC_025150	3.10	7.47E-66	6.53E-63
	ENSSSCG00000050649	1.78	3.49E-25	7.64E-23
	XLOC_026156	2.14	6.06E-24	1.19E-21
	ENSSSCG00000048149	-2.45	1.41E-17	1.56E-15
	XLOC_026048	2.60	8.92E-16	8.19E-14
	XLOC_017479	3.14	8.38E-15	7.09E-13
	ENSSSCG00000024743	-1.29	4.66E-14	3.67E-12
	XLOC_025146	1.49	1.02E-13	7.53E-12
	ENSSSCG00000036096	1.57	1.80E-13	1.28E-11
	ENSSSCG00000046743	2.17	3.97E-12	2.28E-10
	ENSSSCG00000050616	1.07	5.25E-12	2.94E-10
	ENSSSCG00000047250	-1.23	7.43E-12	4.05E-10
	XLOC_799496	-1.46	5.12E-11	2.43E-09
	XLOC_010589	2.30	1.06E-10	4.85E-09
	ENSSSCG00000049323	-1.65	1.11E-09	4.18E-08
	XLOC_012336	1.08	1.43E-09	5.27E-08
	ENSSSCG00000033794	-1.37	1.80E-09	6.51E-08
	XLOC_337383	-1.54	2.98E-09	1.03E-07
	ENSSSCG00000006581	-0.86	3.66E-09	1.25E-07
	ENSSSCG00000049480	1.00	5.21E-09	1.71E-07
(-2)vs-14	ENSSSCG00000050015	-1.05	9.23E-09	2.88E-07
	ENSSSCG00000033537	-0.95	1.41E-08	4.24E-07
	ENSSSCG00000046247	2.09	1.47E-08	4.39E-07
	XLOC_015687	1.43	3.89E-08	1.07E-06
	XLOC_570364	1.63	5.33E-08	1.40E-06
	ENSSSCG00000043982	1.61	7.23E-08	1.84E-06
	XLOC_024743	1.25	9.04E-08	2.25E-06
	ENSSSCG00000045365	1.66	9.64E-08	2.38E-06
	ENSSSCG00000046389	2.94	2.73E-07	6.13E-06
	XLOC_021553	-0.97	6.36E-07	1.33E-05
	XLOC_007183	3.05	6.45E-07	1.34E-05
	XLOC_005186	0.74	1.51E-06	2.90E-05
	ENSSSCG00000045831	1.06	1.55E-06	2.97E-05
	ENSSSCG00000051701	1.40	1.80E-06	3.38E-05
	ENSSSCG00000047361	1.03	1.82E-06	3.41E-05
	ENSSSCG00000051650	-2.01	1.84E-06	3.46E-05
	XLOC_574152	3.10	2.27E-06	4.16E-05
	ENSSSCG00000042618	-0.85	2.28E-06	4.19E-05
	ENSSSCG00000046495	1.27	3.93E-06	6.74E-05
	ENSSSCG00000044425	1.14	5.30E-06	8.85E-05
	ENSSSCG00000039672	1.28	5.82E-06	9.51E-05

ENSSSCG00000041987	0.87	5.95E-06	9.70E-05
ENSSSCG00000042692	1.68	6.68E-06	1.07E-04
ENSSSCG00000046945	-0.57	6.76E-06	1.09E-04
ENSSSCG00000040582	-1.15	6.86E-06	1.10E-04
ENSSSCG00000016379	0.61	1.03E-05	1.58E-04
ENSSSCG00000046608	-1.26	1.33E-05	1.99E-04
XLOC_026859	-0.68	1.40E-05	2.07E-04
XLOC_408080	0.96	2.49E-05	3.44E-04
XLOC_021992	1.00	2.55E-05	3.50E-04
ENSSSCG00000044134	2.31	3.42E-05	4.52E-04
ENSSSCG00000046444	1.79	3.54E-05	4.65E-04
ENSSSCG00000048579	-2.27	3.69E-05	4.83E-04
XLOC_020207	2.11	4.60E-05	5.88E-04
XLOC_009020	0.98	4.78E-05	6.06E-04
XLOC_335607	2.33	4.97E-05	6.23E-04
ENSSSCG00000049534	-0.61	5.92E-05	7.22E-04
XLOC_540073	2.21	6.65E-05	8.04E-04
XLOC_654288	-1.31	6.70E-05	8.07E-04
XLOC_027533	0.57	7.47E-05	8.85E-04
ENSSSCG00000049696	0.70	8.47E-05	9.85E-04
ENSSSCG00000047270	-0.71	8.97E-05	1.04E-03
XLOC_003829	0.92	9.77E-05	1.11E-03
XLOC_014132	0.94	1.19E-04	1.31E-03
ENSSSCG00000049805	1.36	1.28E-04	1.40E-03
XLOC_018030	-1.76	1.35E-04	1.47E-03
XLOC_007694	1.05	1.55E-04	1.64E-03
ENSSSCG00000048678	0.82	1.70E-04	1.78E-03
ENSSSCG00000051023	-0.77	1.86E-04	1.92E-03
ENSSSCG00000045069	-0.56	1.86E-04	1.93E-03
ENSSSCG00000049640	1.13	1.92E-04	1.97E-03
XLOC_570897	2.56	2.00E-04	2.04E-03
ENSSSCG00000050385	1.14	2.23E-04	2.24E-03
ENSSSCG00000048060	1.12	2.28E-04	2.28E-03
ENSSSCG00000046607	-0.82	2.38E-04	2.36E-03
XLOC_504687	1.05	2.45E-04	2.42E-03
ENSSSCG00000041180	-0.78	2.47E-04	2.43E-03
XLOC_018919	-0.69	2.58E-04	2.52E-03
XLOC_005878	-0.69	2.64E-04	2.56E-03
ENSSSCG00000041327	0.77	2.65E-04	2.57E-03
ENSSSCG00000047379	-0.56	2.76E-04	2.66E-03
XLOC_200348	0.93	2.89E-04	2.76E-03
ENSSSCG00000044256	-0.79	3.03E-04	2.87E-03

ENSSSCG00000046815	1.68	3.44E-04	3.19E-03
ENSSSCG00000045657	1.43	4.03E-04	3.66E-03
XLOC_862244	0.98	4.06E-04	3.69E-03
XLOC_012374	0.90	4.29E-04	3.86E-03
XLOC_009655	0.54	4.43E-04	3.97E-03
ENSSSCG00000042438	-0.60	4.51E-04	4.03E-03
XLOC_020627	-1.04	4.75E-04	4.20E-03
ENSSSCG00000045440	-0.73	5.96E-04	5.08E-03
XLOC_014131	0.73	6.08E-04	5.16E-03
ENSSSCG00000033217	-0.97	7.12E-04	5.86E-03
ENSSSCG00000046599	-0.81	7.18E-04	5.89E-03
ENSSSCG00000039815	-0.64	7.20E-04	5.91E-03
ENSSSCG00000042332	0.80	7.68E-04	6.22E-03
ENSSSCG00000048724	1.34	7.73E-04	6.25E-03
ENSSSCG00000034251	1.02	7.79E-04	6.29E-03
ENSSSCG00000041560	1.57	7.89E-04	6.35E-03
ENSSSCG00000045280	1.03	7.90E-04	6.36E-03
ENSSSCG00000045485	0.43	8.71E-04	6.89E-03
ENSSSCG00000046723	0.95	9.57E-04	7.49E-03
ENSSSCG00000048177	-0.93	9.78E-04	7.62E-03
ENSSSCG00000045200	-0.54	1.04E-03	8.02E-03
ENSSSCG00000048693	1.34	1.05E-03	8.05E-03
ENSSSCG00000049698	-0.94	1.24E-03	9.28E-03
ENSSSCG00000044141	-0.57	1.25E-03	9.32E-03
XLOC_800414	1.52	1.26E-03	9.34E-03
ENSSSCG00000011196	0.49	1.34E-03	9.88E-03
ENSSSCG00000043747	0.94	1.40E-03	1.02E-02
XLOC_1094570	-0.79	1.41E-03	1.03E-02
ENSSSCG00000044841	-0.38	1.69E-03	1.19E-02
XLOC_407048	-0.98	1.72E-03	1.21E-02
XLOC_021249	-0.95	1.77E-03	1.24E-02
XLOC_017835	-0.45	2.11E-03	1.43E-02
XLOC_003732	-0.67	2.15E-03	1.44E-02
ENSSSCG00000041508	1.02	2.58E-03	1.67E-02
XLOC_024420	1.12	2.60E-03	1.68E-02
ENSSSCG00000016343	-0.76	2.70E-03	1.73E-02
XLOC_017805	0.74	2.83E-03	1.80E-02
ENSSSCG00000043173	-0.68	2.87E-03	1.83E-02
ENSSSCG00000042663	-0.38	3.02E-03	1.90E-02
XLOC_535646	1.95	3.18E-03	1.97E-02
XLOC_014830	0.45	3.25E-03	2.01E-02
ENSSSCG00000047147	-0.54	3.41E-03	2.10E-02

ENSSSCG00000048420	-0.50	3.51E-03	2.14E-02
ENSSSCG00000044985	0.99	3.96E-03	2.35E-02
XLOC_004739	0.42	4.03E-03	2.38E-02
XLOC_025410	0.69	4.12E-03	2.42E-02
ENSSSCG00000049610	0.58	4.48E-03	2.59E-02
XLOC_963181	-1.08	4.59E-03	2.64E-02
ENSSSCG00000042185	0.59	4.73E-03	2.70E-02
ENSSSCG00000042000	-0.79	4.79E-03	2.73E-02
XLOC_862886	-1.18	4.82E-03	2.74E-02
XLOC_025586	-1.17	4.83E-03	2.74E-02
XLOC_002435	0.88	4.86E-03	2.76E-02
ENSSSCG00000050339	0.67	4.98E-03	2.81E-02
ENSSSCG00000036880	0.34	5.03E-03	2.83E-02
ENSSSCG00000041317	-0.77	5.21E-03	2.91E-02
ENSSSCG00000048215	0.81	5.95E-03	3.23E-02
XLOC_126357	-0.72	6.26E-03	3.37E-02
ENSSSCG00000046669	0.73	6.33E-03	3.40E-02
XLOC_021342	-0.37	6.38E-03	3.43E-02
ENSSSCG00000051016	-0.65	6.79E-03	3.60E-02
ENSSSCG00000033707	-0.59	6.86E-03	3.63E-02
ENSSSCG00000050217	0.74	6.91E-03	3.65E-02
ENSSSCG00000047986	0.96	6.96E-03	3.67E-02
ENSSSCG00000037852	1.13	6.96E-03	3.67E-02
XLOC_799638	0.86	7.35E-03	3.83E-02
XLOC_016636	0.67	7.50E-03	3.90E-02
ENSSSCG00000037520	-0.80	7.67E-03	3.96E-02
ENSSSCG00000048031	0.73	7.75E-03	4.00E-02
ENSSSCG00000043832	0.65	7.77E-03	4.01E-02
ENSSSCG00000051746	1.04	7.84E-03	4.03E-02
ENSSSCG00000044764	0.57	8.04E-03	4.11E-02
ENSSSCG00000048434	-0.46	8.21E-03	4.17E-02
ENSSSCG00000047067	-0.36	8.21E-03	4.17E-02
ENSSSCG00000050213	0.71	8.22E-03	4.18E-02
XLOC_014650	0.70	8.61E-03	4.32E-02
XLOC_027699	-1.08	8.78E-03	4.38E-02
XLOC_025886	-0.94	9.13E-03	4.51E-02
ENSSSCG00000009638	-0.47	9.19E-03	4.54E-02
ENSSSCG00000042439	0.89	9.32E-03	4.58E-02
ENSSSCG00000043175	-0.40	9.43E-03	4.62E-02
ENSSSCG00000048405	0.99	9.49E-03	4.65E-02
XLOC_006071	0.58	9.50E-03	4.65E-02
ENSSSCG00000050419	0.69	9.64E-03	4.70E-02

	ENSSSCG00000046947	-0.71	9.97E-03	4.80E-02
	ENSSSCG00000036505	0.31	1.05E-02	4.99E-02
(+)1vs-14	XLOC_024728	5.65	1.44E-33	5.47E-31
	ENSSSCG00000051193	4.84	4.73E-33	1.71E-30
	XLOC_025150	3.66	2.21E-32	7.44E-30
	ENSSSCG00000050015	-2.92	3.25E-27	8.50E-25
	XLOC_026156	2.96	5.70E-26	1.37E-23
	ENSSSCG00000045292	5.29	4.80E-22	8.08E-20
	ENSSSCG00000044134	4.80	1.25E-20	1.83E-18
	ENSSSCG00000047944	5.54	5.41E-20	7.59E-18
	XLOC_574152	5.26	2.21E-18	2.62E-16
	XLOC_012200	8.24	4.14E-17	4.21E-15
	ENSSSCG00000036096	2.64	8.03E-17	7.66E-15
	ENSSSCG00000033794	-3.76	6.61E-16	5.82E-14
	XLOC_017835	-2.57	1.26E-15	1.08E-13
	ENSSSCG00000042665	3.47	2.21E-15	1.84E-13
	XLOC_540073	4.33	7.29E-15	5.61E-13
	XLOC_015687	2.38	1.45E-13	9.53E-12
	XLOC_015106	-2.42	2.44E-13	1.53E-11
	ENSSSCG00000051426	2.70	1.09E-12	6.37E-11
	XLOC_010030	-4.11	2.33E-12	1.31E-10
	ENSSSCG00000045280	2.77	1.46E-11	7.48E-10
	XLOC_017479	3.35	1.71E-11	8.68E-10
	ENSSSCG00000048149	-3.31	5.24E-11	2.47E-09
	XLOC_198536	-2.95	1.31E-10	5.74E-09
	ENSSSCG00000048060	3.04	4.36E-10	1.72E-08
	ENSSSCG00000046495	2.45	5.40E-10	2.07E-08
	XLOC_198314	2.82	1.20E-09	4.32E-08
	XLOC_025146	1.77	3.81E-09	1.25E-07
	XLOC_017432	-1.63	5.49E-09	1.76E-07
	ENSSSCG00000044913	1.68	9.46E-09	2.91E-07
	ENSSSCG00000044986	-2.33	1.15E-08	3.44E-07
	ENSSSCG00000049805	2.67	1.26E-08	3.74E-07
	ENSSSCG00000046806	1.99	3.72E-08	9.98E-07
	ENSSSCG00000045414	2.20	3.74E-08	9.99E-07
	XLOC_010589	3.41	4.08E-08	1.08E-06
	ENSSSCG00000041918	2.43	4.53E-08	1.18E-06
	ENSSSCG00000048356	-5.13	4.56E-08	1.19E-06
	ENSSSCG00000047706	-2.79	6.13E-08	1.55E-06
	ENSSSCG00000028695	-2.76	7.59E-08	1.87E-06

ENSSSCG00000043103	-2.42	9.81E-08	2.40E-06
XLOC_125594	2.99	1.27E-07	3.01E-06
XLOC_025586	-3.67	1.74E-07	4.00E-06
XLOC_654288	-2.44	2.49E-07	5.53E-06
ENSSSCG00000050649	2.78	2.87E-07	6.30E-06
ENSSSCG00000040582	-2.33	3.03E-07	6.63E-06
ENSSSCG00000050204	2.31	3.14E-07	6.80E-06
ENSSSCG00000049385	-2.39	3.26E-07	7.02E-06
ENSSSCG00000042692	2.30	3.50E-07	7.49E-06
XLOC_012977	2.61	3.91E-07	8.31E-06
XLOC_018195	6.70	5.06E-07	1.05E-05
ENSSSCG00000045831	1.82	5.33E-07	1.10E-05
XLOC_012336	1.24	5.78E-07	1.18E-05
ENSSSCG00000042332	1.74	9.24E-07	1.78E-05
ENSSSCG00000051701	2.16	9.33E-07	1.79E-05
ENSSSCG00000044141	-1.48	1.03E-06	1.96E-05
ENSSSCG00000039815	-1.39	1.03E-06	1.96E-05
ENSSSCG00000041401	-1.14	1.26E-06	2.33E-05
XLOC_015127	1.85	1.57E-06	2.82E-05
ENSSSCG00000046389	3.54	2.03E-06	3.55E-05
ENSSSCG00000049828	2.28	2.65E-06	4.51E-05
XLOC_570897	3.82	2.70E-06	4.58E-05
ENSSSCG00000044425	2.00	2.71E-06	4.60E-05
ENSSSCG00000043982	1.99	3.18E-06	5.33E-05
ENSSSCG00000024743	-2.16	3.43E-06	5.63E-05
XLOC_504687	1.82	3.45E-06	5.66E-05
XLOC_020207	2.40	3.59E-06	5.86E-05
ENSSSCG00000049185	2.53	3.61E-06	5.88E-05
ENSSSCG00000046723	1.72	3.86E-06	6.23E-05
ENSSSCG00000046791	2.55	4.01E-06	6.42E-05
ENSSSCG00000041987	1.35	4.33E-06	6.89E-05
XLOC_408951	-1.84	4.56E-06	7.23E-05
ENSSSCG00000044841	-1.10	5.51E-06	8.56E-05
ENSSSCG00000045154	-2.12	6.09E-06	9.36E-05
ENSSSCG00000042369	1.47	6.12E-06	9.39E-05
ENSSSCG00000049323	-2.26	8.80E-06	1.29E-04
XLOC_862886	-3.03	9.92E-06	1.42E-04
ENSSSCG00000050385	1.81	1.09E-05	1.54E-04
ENSSSCG00000050122	2.99	1.59E-05	2.15E-04
ENSSSCG00000048693	1.93	1.80E-05	2.38E-04

XLOC_200348	1.67	1.81E-05	2.39E-04
ENSSSCG00000048678	1.70	1.98E-05	2.58E-04
ENSSSCG00000047638	1.82	2.05E-05	2.66E-04
ENSSSCG00000049887	1.51	2.15E-05	2.78E-04
XLOC_018270	-1.88	2.28E-05	2.91E-04
ENSSSCG00000048111	-1.74	3.25E-05	3.95E-04
ENSSSCG00000041824	2.57	3.27E-05	3.97E-04
XLOC_007183	3.70	3.34E-05	4.03E-04
XLOC_009810	-1.53	3.59E-05	4.30E-04
ENSSSCG00000045417	1.59	3.72E-05	4.44E-04
ENSSSCG00000047147	-1.29	3.92E-05	4.65E-04
XLOC_126357	-2.35	4.05E-05	4.77E-04
ENSSSCG00000048264	-1.73	5.31E-05	6.03E-04
ENSSSCG00000047088	2.25	5.78E-05	6.45E-04
ENSSSCG00000047379	-1.08	1.05E-04	1.09E-03
ENSSSCG00000044662	-1.83	1.06E-04	1.10E-03
ENSSSCG00000048215	1.50	1.10E-04	1.13E-03
ENSSSCG00000043650	-2.58	1.11E-04	1.13E-03
XLOC_018919	-1.50	1.11E-04	1.14E-03
ENSSSCG00000042438	-1.28	1.28E-04	1.28E-03
ENSSSCG00000016343	-1.77	1.35E-04	1.34E-03
ENSSSCG00000051650	-2.83	1.67E-04	1.61E-03
ENSSSCG00000046461	-0.88	1.86E-04	1.77E-03
XLOC_020360	1.64	1.93E-04	1.82E-03
XLOC_335607	2.65	1.96E-04	1.85E-03
XLOC_407450	2.29	1.98E-04	1.87E-03
XLOC_002768	0.89	2.87E-04	2.56E-03
ENSSSCG00000049481	1.30	3.35E-04	2.92E-03
ENSSSCG00000050213	1.31	3.77E-04	3.24E-03
XLOC_005742	2.36	3.80E-04	3.26E-03
ENSSSCG00000011196	0.86	3.94E-04	3.35E-03
XLOC_027533	0.87	3.98E-04	3.38E-03
ENSSSCG00000042439	1.46	4.10E-04	3.46E-03
XLOC_963181	-2.03	4.35E-04	3.63E-03
XLOC_025886	-2.01	4.41E-04	3.67E-03
XLOC_007547	1.53	4.87E-04	4.00E-03
XLOC_012823	1.42	5.07E-04	4.11E-03
XLOC_026157	1.60	5.51E-04	4.41E-03
ENSSSCG00000044109	-1.56	5.69E-04	4.54E-03
XLOC_017805	1.29	5.72E-04	4.56E-03

ENSSSCG00000046624	1.29	5.93E-04	4.70E-03
ENSSSCG00000048031	1.44	6.51E-04	5.09E-03
XLOC_018266	-1.37	7.05E-04	5.44E-03
XLOC_570364	1.36	7.09E-04	5.46E-03
ENSSSCG00000046669	1.22	7.34E-04	5.62E-03
XLOC_408080	1.45	7.41E-04	5.67E-03
ENSSSCG00000047067	-0.92	7.49E-04	5.71E-03
ENSSSCG00000028322	2.03	8.03E-04	6.07E-03
ENSSSCG00000045895	-1.30	8.10E-04	6.12E-03
ENSSSCG00000045128	1.00	8.47E-04	6.34E-03
ENSSSCG00000005094	0.84	8.89E-04	6.58E-03
ENSSSCG00000045865	1.33	9.25E-04	6.82E-03
ENSSSCG00000045657	1.70	9.32E-04	6.86E-03
ENSSSCG00000042000	-1.29	9.81E-04	7.15E-03
ENSSSCG00000042667	-1.91	1.02E-03	7.38E-03
ENSSSCG00000043219	-1.39	1.16E-03	8.18E-03
ENSSSCG00000042468	-1.41	1.17E-03	8.25E-03
ENSSSCG00000049319	-1.11	1.20E-03	8.41E-03
ENSSSCG00000042185	0.96	1.30E-03	9.08E-03
ENSSSCG00000045440	-1.40	1.35E-03	9.35E-03
ENSSSCG00000048711	1.10	1.35E-03	9.36E-03
ENSSSCG00000051033	1.34	1.54E-03	1.04E-02
XLOC_021553	-1.50	1.57E-03	1.06E-02
XLOC_003621	1.52	1.58E-03	1.07E-02
ENSSSCG00000043766	-1.12	1.60E-03	1.08E-02
XLOC_800414	1.75	1.66E-03	1.11E-02
ENSSSCG00000047985	-0.77	1.76E-03	1.16E-02
ENSSSCG00000046607	-1.20	1.80E-03	1.19E-02
ENSSSCG00000034251	1.36	1.83E-03	1.20E-02
ENSSSCG00000042176	-1.76	1.91E-03	1.25E-02
ENSSSCG00000041508	1.44	1.92E-03	1.26E-02
ENSSSCG00000039672	1.21	2.00E-03	1.30E-02
ENSSSCG00000048905	-2.28	2.05E-03	1.33E-02
XLOC_005186	0.87	2.06E-03	1.33E-02
ENSSSCG00000047625	1.66	2.12E-03	1.36E-02
ENSSSCG00000035352	1.14	2.13E-03	1.37E-02
XLOC_570259	1.40	2.14E-03	1.37E-02
ENSSSCG00000043175	-0.82	2.15E-03	1.38E-02
ENSSSCG00000030438	0.70	2.33E-03	1.47E-02
XLOC_027163	0.86	2.38E-03	1.50E-02

ENSSSCG00000046945	-0.73	2.42E-03	1.51E-02
ENSSSCG00000048101	1.59	2.45E-03	1.53E-02
ENSSSCG00000043494	1.29	2.54E-03	1.58E-02
ENSSSCG00000046352	-1.37	2.58E-03	1.60E-02
XLOC_1094570	-1.31	2.64E-03	1.63E-02
ENSSSCG00000047250	-1.07	2.64E-03	1.63E-02
ENSSSCG00000033537	-0.96	2.91E-03	1.76E-02
ENSSSCG00000047090	-1.44	3.02E-03	1.81E-02
ENSSSCG00000048856	0.74	3.07E-03	1.83E-02
ENSSSCG00000046947	1.82	3.14E-03	1.87E-02
XLOC_005330	0.96	3.33E-03	1.97E-02
XLOC_337115	1.20	3.33E-03	1.97E-02
ENSSSCG00000047349	1.38	3.70E-03	2.14E-02
ENSSSCG00000049464	1.63	3.73E-03	2.16E-02
ENSSSCG00000048754	-1.61	3.79E-03	2.19E-02
ENSSSCG00000016379	0.91	3.97E-03	2.27E-02
XLOC_017544	1.03	3.98E-03	2.27E-02
XLOC_731466	2.71	4.06E-03	2.31E-02
ENSSSCG00000036880	0.68	4.48E-03	2.51E-02
ENSSSCG00000051523	2.16	4.58E-03	2.55E-02
ENSSSCG00000047270	-0.74	4.64E-03	2.57E-02
XLOC_014132	1.66	4.66E-03	2.58E-02
ENSSSCG00000051296	-1.41	4.67E-03	2.58E-02
XLOC_005499	1.02	5.48E-03	2.95E-02
ENSSSCG00000044255	1.07	5.61E-03	3.00E-02
ENSSSCG00000051023	-0.86	5.66E-03	3.02E-02
ENSSSCG00000049640	1.10	5.68E-03	3.04E-02
ENSSSCG00000050616	0.83	5.69E-03	3.04E-02
ENSSSCG00000021146	-1.11	6.04E-03	3.18E-02
ENSSSCG00000046766	-1.16	6.21E-03	3.25E-02
ENSSSCG00000041461	0.77	6.21E-03	3.25E-02
ENSSSCG00000041015	1.03	6.55E-03	3.38E-02
ENSSSCG00000044985	1.18	7.07E-03	3.58E-02
ENSSSCG00000048579	-1.69	7.28E-03	3.67E-02
ENSSSCG00000041370	-1.29	7.70E-03	3.84E-02
ENSSSCG00000038429	1.43	7.96E-03	3.94E-02
ENSSSCG00000042043	1.27	8.00E-03	3.95E-02
ENSSSCG00000046743	1.48	8.32E-03	4.07E-02
ENSSSCG00000043747	1.19	8.70E-03	4.22E-02
ENSSSCG00000041050	0.76	8.98E-03	4.32E-02

XLOC_728787	1.97	9.00E-03	4.33E-02
XLOC_026859	-0.70	9.19E-03	4.40E-02
ENSSSCG00000041866	1.16	9.33E-03	4.45E-02
XLOC_024743	-1.08	9.38E-03	4.46E-02
ENSSSCG00000049532	-1.10	9.79E-03	4.62E-02
ENSSSCG00000044736	0.94	1.00E-02	4.70E-02
XLOC_009194	-0.69	1.02E-02	4.77E-02
XLOC_799496	-0.92	1.07E-02	4.97E-02

Table S4 The predicted target genes of DE LncRNAs

(-10)vs-14			(-6)vs-14			(-2)vs-14			(+)1vs-14		
DE LncRNAs	Target genes	Gene symbol	DE LncRNAs	Target genes	Gene symbol	DE LncRNAs	Target genes	Gene symbol	DE LncRNAs	Target genes	Gene symbol
XLOC_025146	ENSSSCG00000009261*	*CSN2*	ENSSSCG00000045128	ENSSSCG00000000171*	*CKAP4*	XLOC_018919	ENSSSCG00000000114*	*PICK1*	XLOC_018919	ENSSSCG00000000114*	*PICK1*
	ENSSSCG00000009262*	*CSN1S1*	XLOC_026859	ENSSSCG00000000220*	*POU6F1*	XLOC_026859	ENSSSCG00000000220*	*POU6F1*	ENSSSCG00000045128	ENSSSCG00000000171*	*CKAP4*
XLOC_025150	ENSSSCG00000009266*	*ODAM*		ENSSSCG00000000223	*BIN2*		ENSSSCG00000000223	*BIN2*	XLOC_026859	ENSSSCG00000000220*	*POU6F1*
	ENSSSCG00000009267*	*CSN3*	XLOC_021553	ENSSSCG00000003547*	*NCMAP*	ENSSSCG00000044141	ENSSSCG00000000521*	*PHLDA1*		ENSSSCG00000000223	*BIN2*
	ENSSSCG00000039238	*CABS1*	XLOC_021992	ENSSSCG00000003755*	*MCOLN2*		ENSSSCG00000000522*	*NAP1L1*	ENSSSCG00000044141	ENSSSCG00000000521*	*PHLDA1*
XLOC_026156	ENSSSCG00000015405*	*CD36*	ENSSSCG00000041987	ENSSSCG00000003949*	*CDC20*	XLOC_963181	ENSSSCG00000001657*	*CUL7*		ENSSSCG00000000522*	*NAP1L1*
ENSSSCG00000024743	ENSSSCG00000017516	*SP2*		ENSSSCG00000003951*	*C1orf210*		ENSSSCG00000001658	*MRPL2*	XLOC_963181	ENSSSCG00000001657*	*CUL7*
XLOC_027177	ENSSSCG00000037775*	*NA*		ENSSSCG00000030177*	*EBNA1BP2*		ENSSSCG00000001659	*KLC4*		ENSSSCG00000001658	*MRPL2*
	ENSSSCG00000040849*	*NA*	ENSSSCG00000042663	ENSSSCG00000004279*	*OGFRL1*		ENSSSCG00000001660*	*PTK7*		ENSSSCG00000001659	*KLC4*
				ENSSSCG00000019520	*ssc-mir-30c-2*	ENSSSCG00000047067	ENSSSCG00000002285	*GPHN*		ENSSSCG00000001660*	*PTK7*
			ENSSSCG00000040582	ENSSSCG00000005904	*VPS28*		ENSSSCG00000002287	*MPP5*	ENSSSCG00000043650	ENSSSCG00000002257*	*MCTP2*
				ENSSSCG00000005907	*ADCK5*		ENSSSCG00000033295*	*EIF2S1*		ENSSSCG00000035989	*NA*
				ENSSSCG00000005908	*SLC52A2*	ENSSSCG00000050616	ENSSSCG00000002375*	*RPS6KL1*	ENSSSCG00000047067	ENSSSCG00000002285	*GPHN*
				ENSSSCG00000005909	*FBXL6*	ENSSSCG00000051016	ENSSSCG00000002440*	*CCDC88C*		ENSSSCG00000002287	*MPP5*
				ENSSSCG00000005910*	*TMEM249*		ENSSSCG00000034114	*GPR68*		ENSSSCG00000033295*	*EIF2S1*
				ENSSSCG00000005917	*HSF1*	ENSSSCG00000044425	ENSSSCG00000002953*	*NA*	ENSSSCG00000050616	ENSSSCG00000002375*	*RPS6KL1*
				ENSSSCG00000005920*	*NA*		ENSSSCG00000002955*	*CATSPERG*	ENSSSCG00000041015	ENSSSCG00000002671*	*ATP2C2*
				ENSSSCG00000025116*	*TONSL*		ENSSSCG00000002959	*FAM98C*		ENSSSCG00000002672*	*MEAK7*
				ENSSSCG00000046182	*NA*		ENSSSCG00000038902*	*KCNK6*		ENSSSCG00000002937	*NA*
			ENSSSCG00000006581	ENSSSCG00000006578	*S100A4*	XLOC_020627	ENSSSCG00000002965*	*ACTN4*	ENSSSCG00000046766	ENSSSCG00000029420	*ZNF569*
				ENSSSCG00000006582	*S100A14*	ENSSSCG00000050217	ENSSSCG00000003278	*NA*		ENSSSCG00000029600*	*NA*
				ENSSSCG00000028553	*ILF2*	XLOC_021249	ENSSSCG00000003329*	*ACAP3*	ENSSSCG00000044425	ENSSSCG00000002953*	*NA*

XLOC_012336	ENSSSCG00000007469	PTPN1		ENSSSCG00000003342	DVL1		ENSSSCG00000002955*	CATSPERG
	ENSSSCG00000027206	PARD6B		ENSSSCG00000003344	VWA1		ENSSSCG00000002959	FAM98C
XLOC_012374	ENSSSCG00000007494	CSTF1		ENSSSCG00000003346*	ANKRD65		ENSSSCG00000038902*	KCNK6
	ENSSSCG00000007497*	GCNT7		ENSSSCG00000021880	MXRA8	XLOC_021553	ENSSSCG00000003547*	NCMAP
	ENSSSCG00000007713	BUD23	XLOC_021553	ENSSSCG00000003547*	NCMAP	ENSSSCG00000047147	ENSSSCG00000003577	WASF2
	ENSSSCG00000007715	ABHD11	ENSSSCG00000047147	ENSSSCG00000003577	WASF2	ENSSSCG00000042438	ENSSSCG00000003577	WASF2
XLOC_015687	ENSSSCG00000007717*	METTL27	ENSSSCG00000042438	ENSSSCG00000003577	WASF2		ENSSSCG00000003578	FGR
	ENSSSCG00000033358*	VPS37D		ENSSSCG00000003578	FGR		ENSSSCG00000003701*	SNRPD1
	ENSSSCG00000039800	ssc-mir-7137		ENSSSCG00000003701*	SNRPD1	ENSSSCG00000042000	ENSSSCG00000025478	MIB1
XLOC_024743	ENSSSCG00000008973*	NA	ENSSSCG00000042000	ENSSSCG00000025478	MIB1		ENSSSCG00000028010	ABHD3
XLOC_025146	ENSSSCG00000009261*	CSN2		ENSSSCG00000028010	ABHD3		ENSSSCG00000003949*	CDC20
	ENSSSCG00000009262*	CSN1S1	ENSSSCG00000041327	ENSSSCG00000003755*	MCOLN2	ENSSSCG00000041987	ENSSSCG00000003951*	C1orf210
	ENSSSCG00000009266*	ODAM	XLOC_021992	ENSSSCG00000003755*	MCOLN2		ENSSSCG00000030177*	EBNA1BP2
XLOC_025150	ENSSSCG00000009267*	CSN3		ENSSSCG00000003949*	CDC20	ENSSSCG00000044841	ENSSSCG00000004293	SNX14
	ENSSSCG00000039238	CABS1	ENSSSCG00000041987	ENSSSCG00000003951*	C1orf210		ENSSSCG00000004294*	SYNCRIP
XLOC_009020	ENSSSCG00000010370*	ANXA8		ENSSSCG00000030177*	EBNA1BP2	XLOC_002768	ENSSSCG00000004657*	CEP152
	ENSSSCG00000043778*	NA		ENSSSCG00000004279*	OGFRL1	ENSSSCG00000005094	ENSSSCG00000005095	PRKCH
XLOC_014132	ENSSSCG00000013839	RASAL3	ENSSSCG00000042663	ENSSSCG00000019520	ssc-mir-30c-2	ENSSSCG00000051701	ENSSSCG00000005191*	MPDZ
	ENSSSCG00000023607	CYP4F22	ENSSSCG00000044841	ENSSSCG00000004293	SNX14	ENSSSCG00000042369	ENSSSCG00000005383*	ALG2
XLOC_026156	ENSSSCG00000015405*	CD36		ENSSSCG00000004294*	SYNCRIP	ENSSSCG00000048111	ENSSSCG00000005582	STRBP
ENSSSCG00000048149	ENSSSCG00000016232	MRPL44	ENSSSCG00000051701	ENSSSCG00000005191*	MPDZ		ENSSSCG00000005904	VPS28
ENSSSCG00000042185	ENSSSCG00000016378	PASK	XLOC_002435	ENSSSCG00000005707*	FIBCD1		ENSSSCG00000005907	ADCK5
ENSSSCG00000047513	ENSSSCG00000016381*	SNED1		ENSSSCG00000005904	VPS28	ENSSSCG00000040582	ENSSSCG00000005908	SLC52A2
XLOC_408080	ENSSSCG00000016381*	SNED1	ENSSSCG00000040582	ENSSSCG00000005907	ADCK5		ENSSSCG00000005909	FBXL6
ENSSSCG00000046607	ENSSSCG00000016430	GALNT11		ENSSSCG00000005908	SLC52A2		ENSSSCG00000005910*	TMEM249

	ENSSSCG00000016431*	*GALNTL5*		ENSSSCG00000005909	*FBXL6*		ENSSSCG00000005917	*HSF1*
	ENSSSCG00000016432*	*PRKAG2*		ENSSSCG00000005910*	*TMEM249*		ENSSSCG00000005920*	*NA*
ENSSSCG00000048060	ENSSSCG00000016894*	*ARL15*		ENSSSCG00000005917	*HSF1*		ENSSSCG00000025116*	*TONSL*
	ENSSSCG00000017177	*ST6GALNAC2*		ENSSSCG00000005920*	*NA*		ENSSSCG00000046182	*NA*
ENSSSCG00000042682	ENSSSCG00000017181	*CYGB*		ENSSSCG00000025116*	*TONSL*	XLOC_017805	ENSSSCG00000006307*	*RCSD1*
	ENSSSCG00000019245	*NA*		ENSSSCG00000046182	*NA*	XLOC_017835	ENSSSCG00000006347*	*DUSP12*
	ENSSSCG00000023362*	*RHBDF2*	XLOC_017805	ENSSSCG00000006307*	*RCSD1*		ENSSSCG00000006350*	*FCGR2B*
ENSSSCG00000050015	ENSSSCG00000017251*	*SOX9*	XLOC_017835	ENSSSCG00000006347*	*DUSP12*	ENSSSCG00000044662	ENSSSCG00000006487	*CCT3*
	ENSSSCG00000017376*	*MEOX1*		ENSSSCG00000006350*	*FCGR2B*		ENSSSCG00000006490	*SMG5*
XLOC_005878	ENSSSCG00000017378	*DHX8*		ENSSSCG00000006572*	*NPR1*	ENSSSCG00000043747	ENSSSCG00000006665	*SF3B4*
	ENSSSCG00000017379*	*ETV4*	XLOC_018030	ENSSSCG00000006578	*S100A4*	XLOC_018195	ENSSSCG00000006748*	*TSPAN2*
ENSSSCG00000024743	ENSSSCG00000017516	*SP2*		ENSSSCG00000006582	*S100A14*		ENSSSCG00000007206	*RBCK1*
ENSSSCG00000046841	ENSSSCG00000017904	*ENO3*		ENSSSCG00000028553	*ILF2*	ENSSSCG00000047985	ENSSSCG00000007212*	*C20orf96*
	ENSSSCG00000017908*	*GP1BA*		ENSSSCG00000006578	*S100A4*		ENSSSCG00000031519	*NRSN2*
ENSSSCG00000043982	ENSSSCG00000019156	*U6*	ENSSSCG00000006581	ENSSSCG00000006582	*S100A14*	XLOC_012200	ENSSSCG00000007344	*KIAA1755*
	ENSSSCG00000020665	*TAF6L*		ENSSSCG00000028553	*ILF2*	XLOC_012336	ENSSSCG00000007469	*PTPN1*
	ENSSSCG00000025513*	*SNORD22*	ENSSSCG00000043747	ENSSSCG00000006665	*SF3B4*		ENSSSCG00000027206	*PARD6B*
ENSSSCG00000043175	ENSSSCG00000026212*	*SNORD29*	XLOC_012336	ENSSSCG00000007469	*PTPN1*		ENSSSCG00000007713	*BUD23*
	ENSSSCG00000026293	*STX5*		ENSSSCG00000027206	*PARD6B*		ENSSSCG00000007715	*ABHD11*
	ENSSSCG00000038711	*TMEM223*		ENSSSCG00000007493*	*AURKA*	XLOC_015687	ENSSSCG00000007717*	*METTL27*
	ENSSSCG00000021536	*CLDN9*	XLOC_535646	ENSSSCG00000007494	*CSTF1*		ENSSSCG00000033358*	*VPS37D*
ENSSSCG00000036096	ENSSSCG00000023304*	*SRRM2*		ENSSSCG00000007496	*RTF2*		ENSSSCG00000039800	*ssc-mir-7137*
	ENSSSCG00000023315	*THOC6*	XLOC_012374	ENSSSCG00000007494	*CSTF1*	ENSSSCG00000043494	ENSSSCG00000007896	*TXNDC11*
	ENSSSCG00000023743*	*CLDN6*		ENSSSCG00000007497*	*GCNT7*	ENSSSCG00000043219	ENSSSCG00000008122	*ADRA2B*
	ENSSSCG00000029264	*PKMYT1*	ENSSSCG00000049698	ENSSSCG00000007585*	*ACTB*		ENSSSCG00000039523	*NA*

	ENSSSCG00000030518*	PRSS33		ENSSSCG00000007713	BUD23	XLOC_024728	ENSSSCG00000008959*	CXCL2
	ENSSSCG00000035052*	KREMEN2		ENSSSCG00000007715	ABHD11		ENSSSCG00000008961	MTHFD2L
ENSSSCG00000051023	ENSSSCG00000021991	NDUFV3	XLOC_015687	ENSSSCG00000007717*	METTL27	XLOC_024743	ENSSSCG00000008973*	NA
	ENSSSCG00000023078*	WDR4		ENSSSCG00000033358*	VPS37D		ENSSSCG00000009261*	CSN2
						XLOC_025146		
ENSSSCG00000047361	ENSSSCG00000023636	TMEM222		ENSSSCG00000039800	ssc-mir-7137		ENSSSCG00000009262*	CSN1S1
	ENSSSCG00000024018	SLC16A3	XLOC_024743	ENSSSCG00000008973*	NA		ENSSSCG00000009266*	ODAM
	ENSSSCG00000033689	GPS1	XLOC_025146	ENSSSCG00000009261*	CSN2	XLOC_025150	ENSSSCG00000009267*	CSN3
	ENSSSCG00000035852	CSNK1D		ENSSSCG00000009262*	CSN1S1		ENSSSCG00000039238	CABS1
XLOC_004739	ENSSSCG00000039873	DCXR		ENSSSCG00000009266*	ODAM	ENSSSCG00000045154	ENSSSCG00000009560*	TFDP1
	ENSSSCG00000040352	DUS1L	XLOC_025150	ENSSSCG00000009267*	CSN3		ENSSSCG00000009563	TMCO3
	ENSSSCG00000040385	RFNG		ENSSSCG00000039238	CABS1	ENSSSCG00000049805	ENSSSCG00000009565*	GAS6
	ENSSSCG00000040503	LRRC45	ENSSSCG00000049805	ENSSSCG00000009565*	GAS6	ENSSSCG00000047090	ENSSSCG00000009653	CDCA2
ENSSSCG00000044256	ENSSSCG00000030102	NA	ENSSSCG00000046495	ENSSSCG00000010143*	MTR	ENSSSCG00000048264	ENSSSCG00000009705*	GALNT7
ENSSSCG00000046599	ENSSSCG00000031780	MAGI2	XLOC_009020	ENSSSCG00000010370*	ANXA8		ENSSSCG00000030118*	SAP30
ENSSSCG00000045200	ENSSSCG00000032199	SNORD88B		ENSSSCG00000043778*	NA	ENSSSCG00000046495	ENSSSCG00000010143*	MTR
	ENSSSCG00000032902	KLK7		ENSSSCG00000010994	NFX1	ENSSSCG00000047944	ENSSSCG00000010816*	TGFB2
ENSSSCG00000048405	ENSSSCG00000033591	CHD9	ENSSSCG00000045657	ENSSSCG00000010995	CHMP5		ENSSSCG00000010994	NFX1
XLOC_570364	ENSSSCG00000038304	RBM4B		ENSSSCG00000010999	SMU1	ENSSSCG00000045657	ENSSSCG00000010995	CHMP5
	ENSSSCG00000038508*	SPTBN2	XLOC_003732	ENSSSCG00000011075	KIAA1217		ENSSSCG00000010999	SMU1
XLOC_799496	ENSSSCG00000038966*	KRT7	ENSSSCG00000045440	ENSSSCG00000011107	CCNY	ENSSSCG00000045440	ENSSSCG00000011107	CCNY
	ENSSSCG00000039587*	NA	XLOC_003829	ENSSSCG00000011125*	GATA3	ENSSSCG00000011196	ENSSSCG00000011195*	GALNT15
XLOC_005792	ENSSSCG00000039878	PLD6	ENSSSCG00000011196	ENSSSCG00000011195*	GALNT15		ENSSSCG00000011197	OXNAD1
				ENSSSCG00000011197	OXNAD1	XLOC_007183	ENSSSCG00000011850*	MUC4
			XLOC_007183	ENSSSCG00000011850*	MUC4		ENSSSCG00000011853	RUBCN
				ENSSSCG00000011853	RUBCN	ENSSSCG00000043103	ENSSSCG00000012071	NA

ENSSSCG00000037520	ENSSSCG00000012767	*HAUS7*	ENSSSCG00000028695	ENSSSCG00000012519	*GPRASP1*
XLOC_014132	ENSSSCG00000013839	*RASAL3*		ENSSSCG00000012520*	*NA*
	ENSSSCG00000023607	*CYP4F22*	XLOC_570259	ENSSSCG00000012836	*AP2A2*
ENSSSCG00000039815	ENSSSCG00000014003	*MIER2*		ENSSSCG00000012842	*RPLP2*
	ENSSSCG00000014007	*FLT4*		ENSSSCG00000012846	*GATD1*
XLOC_014650	ENSSSCG00000014061*	*THOC3*		ENSSSCG00000012847	*TALDO1*
	ENSSSCG00000014062*	*SIMC1*		ENSSSCG00000023933*	*CRACR2B*
XLOC_025886	ENSSSCG00000015267*	*FMO2*	ENSSSCG00000043766	ENSSSCG00000013276	*PRDM11*
	ENSSSCG00000023472	*FMO3*	XLOC_014132	ENSSSCG00000013839	*RASAL3*
XLOC_026156	ENSSSCG00000015405*	*CD36*		ENSSSCG00000023607	*CYP4F22*
XLOC_1094570	ENSSSCG00000015524	*FAM20B*	ENSSSCG00000039815	ENSSSCG00000014003	*MIER2*
	ENSSSCG00000024674	*ABL2*		ENSSSCG00000014007	*FLT4*
XLOC_407048	ENSSSCG00000015780*	*STOX2*	ENSSSCG00000051426	ENSSSCG00000014436*	*ARHGEF37*
XLOC_010589	ENSSSCG00000016223*	*ACSL3*	XLOC_015106	ENSSSCG00000014443*	*CAMK2A*
ENSSSCG00000048149	ENSSSCG00000016232	*MRPL44*	ENSSSCG00000041370	ENSSSCG00000014893	*NARS2*
ENSSSCG00000016379	ENSSSCG00000016370	*SEPTIN2*	XLOC_025886	ENSSSCG00000015267*	*FMO2*
	ENSSSCG00000016378	*PASK*		ENSSSCG00000023472	*FMO3*
	ENSSSCG00000016381*	*SNED1*	ENSSSCG00000049385	ENSSSCG00000015270*	*FMOD*
ENSSSCG00000042185	ENSSSCG00000016378	*PASK*		ENSSSCG00000015271	*PRELP*
XLOC_408080	ENSSSCG00000016381*	*SNED1*	ENSSSCG00000050122	ENSSSCG00000015270*	*FMOD*
ENSSSCG00000044134	ENSSSCG00000016420*	*INSIG1*	XLOC_026156	ENSSSCG00000015405*	*CD36*
ENSSSCG00000046607	ENSSSCG00000016430	*GALNT11*	XLOC_026157	ENSSSCG00000015405*	*CD36*
	ENSSSCG00000016431*	*GALNTL5*	XLOC_1094570	ENSSSCG00000015524	*FAM20B*
	ENSSSCG00000016432*	*PRKAG2*		ENSSSCG00000024674	*ABL2*
ENSSSCG00000042618	ENSSSCG00000016569*	*SMO*	XLOC_009810	ENSSSCG00000015717*	*C1QL2*
	ENSSSCG00000016572	*TNPO3*	XLOC_010589	ENSSSCG00000016223*	*ACSL3*

ENSSSCG00000048060	ENSSSCG00000016894*	*ARL15*	ENSSSCG00000048149	ENSSSCG00000016232	*MRPL44*
ENSSSCG00000050015	ENSSSCG00000017251*	*SOX9*		ENSSSCG00000016370	*SEPTIN2*
ENSSSCG00000033537	ENSSSCG00000017251*	*SOX9*	ENSSSCG00000016379	ENSSSCG00000016378	*PASK*
	ENSSSCG00000037754*	*SLC39A11*		ENSSSCG00000016381*	*SNED1*
	ENSSSCG00000017376*	*MEOX1*	ENSSSCG00000042185	ENSSSCG00000016378	*PASK*
XLOC_005878	ENSSSCG00000017378	*DHX8*	XLOC_408080	ENSSSCG00000016381*	*SNED1*
	ENSSSCG00000017379*	*ETV4*	ENSSSCG00000044134	ENSSSCG00000016420*	*INSIG1*
ENSSSCG00000024743	ENSSSCG00000017516	*SP2*		ENSSSCG00000016430	*GALNT11*
ENSSSCG00000043982	ENSSSCG00000019156	*U6*	ENSSSCG00000046607	ENSSSCG00000016431*	*GALNTL5*
	ENSSSCG00000020665	*TAF6L*		ENSSSCG00000016432*	*PRKAG2*
	ENSSSCG00000025513*	*SNORD22*	XLOC_012823	ENSSSCG00000016549	*MKLN1*
ENSSSCG00000043175	ENSSSCG00000026212*	*SNORD29*	ENSSSCG00000051193	ENSSSCG00000016726*	*ADCY1*
	ENSSSCG00000026293	*STX5*	ENSSSCG00000048060	ENSSSCG00000016894*	*ARL15*
	ENSSSCG00000038711	*TMEM223*	ENSSSCG00000050015	ENSSSCG00000017251*	*SOX9*
	ENSSSCG00000021536	*CLDN9*	ENSSSCG00000033537	ENSSSCG00000017251*	*SOX9*
	ENSSSCG00000023743*	*CLDN6*		ENSSSCG00000037754*	*SLC39A11*
ENSSSCG00000036096	ENSSSCG00000029264	*PKMYT1*	ENSSSCG00000049532	ENSSSCG00000017367	*MPP2*
	ENSSSCG00000030518*	*PRSS33*	ENSSSCG00000024743	ENSSSCG00000017516	*SP2*
	ENSSSCG00000035052*	*KREMEN2*	XLOC_005330	ENSSSCG00000017615	*DGKE*
XLOC_025410	ENSSSCG00000021822*	*RNF169*	XLOC_005499	ENSSSCG00000017783*	*NA*
ENSSSCG00000051023	ENSSSCG00000021991	*NDUFV3*		ENSSSCG00000035445*	*SEZ6*
	ENSSSCG00000023078*	*WDR4*	ENSSSCG00000046461	ENSSSCG00000018031	*ZNF287*
XLOC_027699	ENSSSCG00000022129	*ARSL*	ENSSSCG00000046791	ENSSSCG00000019034*	*ssc-mir-29b-2*
ENSSSCG00000048693	ENSSSCG00000023228*	*NA*	ENSSSCG00000043982	ENSSSCG00000019156	*U6*
	ENSSSCG00000023304*	*SRRM2*	ENSSSCG00000043175	ENSSSCG00000020665	*TAF6L*

	ENSSSCG00000023315	*THOC6*		ENSSSCG00000025513*	*SNORD22*
	ENSSSCG00000040533	*C6H19orf54*		ENSSSCG00000026212*	*SNORD29*
ENSSSCG00000047361	ENSSSCG00000023636	*TMEM222*		ENSSSCG00000026293	*STX5*
	ENSSSCG00000024018	*SLC16A3*		ENSSSCG00000038711	*TMEM223*
	ENSSSCG00000033689	*GPS1*	XLOC_003621	ENSSSCG00000020705*	*MAP3K8*
	ENSSSCG00000035852	*CSNK1D*	ENSSSCG00000041050	ENSSSCG00000020737	*ZNRD2*
XLOC_004739	ENSSSCG00000039873	*DCXR*	ENSSSCG00000048856	ENSSSCG00000020737	*ZNRD2*
	ENSSSCG00000040352	*DUS1L*		ENSSSCG00000021536	*CLDN9*
	ENSSSCG00000040385	*RFNG*		ENSSSCG00000023304*	*SRRM2*
	ENSSSCG00000040503	*LRRC45*		ENSSSCG00000023315	*THOC6*
ENSSSCG00000050339	ENSSSCG00000027144*	*LMNTD1*	ENSSSCG00000036096	ENSSSCG00000023743*	*CLDN6*
XLOC_007694	ENSSSCG00000027357*	*CSTB*		ENSSSCG00000029264	*PKMYT1*
ENSSSCG00000048434	ENSSSCG00000029991	*SNIP1*		ENSSSCG00000030518*	*PRSS33*
	ENSSSCG00000039034*	*DNALI1*		ENSSSCG00000035052*	*KREMEN2*
ENSSSCG00000044256	ENSSSCG00000030102	*NA*	ENSSSCG00000051023	ENSSSCG00000021991	*NDUFV3*
	ENSSSCG00000031166*	*ZNF296*		ENSSSCG00000023078*	*WDR4*
	ENSSSCG00000033194	*GEMIN7*	ENSSSCG00000044109	ENSSSCG00000022347	*WNT11*
ENSSSCG00000047379	ENSSSCG00000035374*	*TRAPPC6A*	ENSSSCG00000048693	ENSSSCG00000023228*	*NA*
	ENSSSCG00000035706*	*NA*		ENSSSCG00000040533	*C6H19orf54*
	ENSSSCG00000037056	*PPP1R37*	ENSSSCG00000047638	ENSSSCG00000026412	*PLXNA2*
	ENSSSCG00000040264	*CLASRP*		ENSSSCG00000027280*	*NA*
	ENSSSCG00000031166*	*ZNF296*	ENSSSCG00000041401	ENSSSCG00000028973	*PHACTR4*
	ENSSSCG00000033194	*GEMIN7*		ENSSSCG00000030284	*TRNAU1AP*
XLOC_862886	ENSSSCG00000035374*	*TRAPPC6A*		ENSSSCG00000030477*	*NA*
	ENSSSCG00000035706*	*NA*	ENSSSCG00000030438	ENSSSCG00000028411	*MC5R*
	ENSSSCG00000037056	*PPP1R37*	XLOC_018266	ENSSSCG00000031125*	*AMPD2*

ENSSSCG00000046599	ENSSSCG00000031780	*MAGI2*	XLOC_018270	ENSSSCG00000031125*	*AMPD2*
ENSSSCG00000045200	ENSSSCG00000032199	*SNORD88B*		ENSSSCG00000037808*	*NA*
	ENSSSCG00000032902	*KLK7*	ENSSSCG00000047379	ENSSSCG00000031166*	*ZNF296*
ENSSSCG00000041317	ENSSSCG00000033099	*ASPRV1*		ENSSSCG00000033194	*GEMIN7*
ENSSSCG00000036880	ENSSSCG00000033193	*TPO*		ENSSSCG00000035374*	*TRAPPC6A*
ENSSSCG00000048405	ENSSSCG00000033591	*CHD9*		ENSSSCG00000035706*	*NA*
ENSSSCG00000046815	ENSSSCG00000034727*	*CCDC24*		ENSSSCG00000037056	*PPP1R37*
XLOC_020207	ENSSSCG00000035388	*C16orf46*		ENSSSCG00000040264	*CLASRP*
ENSSSCG00000034251	ENSSSCG00000037035	*UVSSA*	XLOC_862886	ENSSSCG00000031166*	*ZNF296*
XLOC_570364	ENSSSCG00000038304	*RBM4B*		ENSSSCG00000033194	*GEMIN7*
	ENSSSCG00000038508*	*SPTBN2*		ENSSSCG00000035374*	*TRAPPC6A*
ENSSSCG00000048420	ENSSSCG00000038809	*SNORD116*		ENSSSCG00000035706*	*NA*
	ENSSSCG00000039794	*SNORD116*		ENSSSCG00000037056	*PPP1R37*
XLOC_799496	ENSSSCG00000038966*	*KRT7*	XLOC_020360	ENSSSCG00000032985*	*GOT2*
	ENSSSCG00000039587*	*NA*		ENSSSCG00000037660	*NA*
XLOC_504687	ENSSSCG00000040554*	*COL9A3*	ENSSSCG00000036880	ENSSSCG00000033193	*TPO*
ENSSSCG00000049640	ENSSSCG00000040967	*NACC2*	XLOC_020207	ENSSSCG00000035388	*C16orf46*
			ENSSSCG00000034251	ENSSSCG00000037035	*UVSSA*
			XLOC_198314	ENSSSCG00000037336	*GID4*
				ENSSSCG00000037557	*DRC3*
			XLOC_570364	ENSSSCG00000038304	*RBM4B*
				ENSSSCG00000038508*	*SPTBN2*
			XLOC_799496	ENSSSCG00000038966*	*KRT7*
				ENSSSCG00000039587*	*NA*
			ENSSSCG00000041866	ENSSSCG00000039626*	*DERL3*
			ENSSSCG00000049185	ENSSSCG00000040207*	*P2RY2*

		XLOC_504687	ENSSSCG00000040554*	*COL9A3*
		ENSSSCG00000049640	ENSSSCG00000040967	*NACC2*

Note: * indicates the differentially expressed target genes. NA indicates novel gene.

Table S5 Significant GO and KEGG pathways of the target genes of DE lncRNAs

Database	ID	Term	*P*-value	Genes	Gene symbol
KEGG PATHWAY	ssc00512	Mucin type O-glycan biosynthesis	3.59E-04	ENSSSCG00000011195|ENSSSCG00000016430|ENSSSCG00000016431|ENSSSCG00000009705	*GALNT15|GALNT11|GALNTL5|GALNT7*
KEGG PATHWAY	ssc04530	Tight junction	1.69E-03	ENSSSCG00000016432|ENSSSCG00000002965|ENSSSCG00000002287|ENSSSCG00000005191|ENSSSCG00000007585|ENSSSCG00000023743|ENSSSCG00000012071	*PRKAG2|ACTN4|MPP5|MPDZ|ACTB|CLDN6|NA*
KEGG PATHWAY	ssc04520	Adherens junction	5.82E-03	ENSSSCG00000007469|ENSSSCG00000003577|ENSSSCG00000002965|ENSSSCG00000007585	*PTPN1|WASF2|ACTN4|ACTB*
KEGG PATHWAY	ssc05132	Salmonella infection	7.23E-03	ENSSSCG00000008959|ENSSSCG00000003577|ENSSSCG00000001659|ENSSSCG00000007585	*CXCL2|WASF2|KLC4|ACTB*
KEGG PATHWAY	ssc05217	Basal cell carcinoma	2.05E-02	ENSSSCG00000016569|ENSSSCG00000022347|ENSSSCG00000003342	*SMO|WNT11|DVL1*
KEGG PATHWAY	ssc00670	One carbon pool by folate	2.58E-02	ENSSSCG00000008961|ENSSSCG00000010143	*MTHFD2L|MTR*
KEGG PATHWAY	ssc04114	Oocyte meiosis	2.77E-02	ENSSSCG00000029264|ENSSSCG00000016726|ENSSSCG00000003949|ENSSSCG00000007493	*PKMYT1|ADCY1|CDC20|AURKA*
KEGG PATHWAY	ssc04714	Thermogenesis	2.99E-02	ENSSSCG00000016432|ENSSSCG00000006572|ENSSSCG00000016726|ENSSSCG00000016223|ENSSSCG00000007585|ENSSSCG00000021991	*PRKAG2|NPR1|ADCY1|ACSL3|ACTB|NDUFV3*
KEGG PATHWAY	ssc05100	Bacterial invasion of epithelial cells	3.68E-02	ENSSSCG00000016370|ENSSSCG00000003577|ENSSSCG00000007585	*SEPTIN2|WASF2|ACTB*
KEGG PATHWAY	ssc04920	Adipocytokine signaling pathway	3.83E-02	ENSSSCG00000015405|ENSSSCG00000016223|ENSSSCG00000016432	*CD36|ACSL3|PRKAG2*
KEGG PATHWAY	ssc04390	Hippo signaling pathway	3.96E-02	ENSSSCG00000002287|ENSSSCG00000022347|ENSSSCG00000003342|ENSSSCG00000007585	*MPP5|WNT11|DVL1|ACTB*
KEGG PATHWAY	ssc03040	Spliceosome	4.53E-02	ENSSSCG00000014061|ENSSSCG00000006665|ENSSSCG00000017378|ENSSSCG00000003701	*THOC3|SF3B4|DHX8|SNRPD1*
KEGG PATHWAY	ssc01230	Biosynthesis of amino acids	4.77E-02	ENSSSCG00000012847|ENSSSCG00000017904|ENSSSCG00000010143	*TALDO1|ENO3|MTR*

Gene Ontology	GO:0030879	Mammary gland development	3.92E-05	ENSSSCG00000009267|ENSSSCG00000016569|ENSSSCG00000009261|ENSSSCG00000017251|ENSSSCG00000002671	*CSN3|SMO|CSN2|SOX9|ATP2C2*
Gene Ontology	GO:0010001	Glial cell differentiation	5.21E-05	ENSSSCG00000002287|ENSSSCG00000016569|ENSSSCG00000006748|ENSSSCG00000017251|ENSSSCG00000021880	*MPP5|SMO|TSPAN2|SOX9|MXRA8*
Gene Ontology	GO:0021782	Glial cell development	6.93E-05	ENSSSCG00000002287|ENSSSCG00000016569|ENSSSCG00000006748|ENSSSCG00000021880	*MPP5|SMO|TSPAN2|MXRA8*
Gene Ontology	GO:0042063	Gliogenesis	1.25E-04	ENSSSCG00000002287|ENSSSCG00000016569|ENSSSCG00000006748|ENSSSCG00000017251|ENSSSCG00000021880	*MPP5|SMO|TSPAN2|SOX9|MXRA8*
Gene Ontology	GO:0042472	Inner ear morphogenesis	1.73E-04	ENSSSCG00000001660|ENSSSCG00000016420|ENSSSCG00000017251|ENSSSCG00000003342	*PTK7|INSIG1|SOX9|DVL1*
Gene Ontology	GO:0051130	Positive regulation of cellular component organization	2.66E-04	ENSSSCG00000000114|ENSSSCG00000002965|ENSSSCG00000003949|ENSSSCG00000003577|ENSSSCG00000003342|ENSSSCG00000012520|ENSSSCG00000015524|ENSSSCG00000017251|ENSSSCG00000001660|ENSSSCG00000003701	*PICK1|ACTN4|CDC20|WASF2|DVL1|NA|FAM20B|SOX9|PTK7|SNRPD1*
Gene Ontology	GO:0090103	Cochlea morphogenesis	3.00E-04	ENSSSCG00000001660|ENSSSCG00000017251|ENSSSCG00000003342	*PTK7|SOX9|DVL1*
Gene Ontology	GO:0042471	Ear morphogenesis	4.08E-04	ENSSSCG00000001660|ENSSSCG00000016420|ENSSSCG00000017251|ENSSSCG00000003342	*PTK7|INSIG1|SOX9|DVL1*
Gene Ontology	GO:0090102	Cochlea development	4.87E-04	ENSSSCG00000001660|ENSSSCG00000017251|ENSSSCG00000003342	*PTK7|SOX9|DVL1*
Gene Ontology	GO:1905330	Regulation of morphogenesis of an epithelium	5.21E-04	ENSSSCG00000001660|ENSSSCG00000016569|ENSSSCG00000017251|ENSSSCG00000003342	*PTK7|SMO|SOX9|DVL1*
Gene Ontology	GO:0048708	Astrocyte differentiation	7.37E-04	ENSSSCG00000006748|ENSSSCG00000017251|ENSSSCG00000016569	*TSPAN2|SOX9|SMO*

Gene Ontology	GO:0051128	Regulation of cellular component organization	7.77E-04	ENSSSCG00000000114|ENSSSCG00000002965|ENSSSCG00000003949|ENSSSCG00000026293|ENSSSCG00000011853|ENSSSCG00000016420|ENSSSCG00000003577|ENSSSCG00000003342|ENSSSCG00000012520|ENSSSCG00000015524|ENSSSCG00000007469|ENSSSCG00000017251|ENSSSCG00000001660|ENSSSCG00000003701	*PICK1|ACTN4|CDC20|STX5|RUBCN|INSIG1|WASF2|DVL1|NA|FAM20B|PTPN1|SOX9|PTK7|SNRPD1*
Gene Ontology	GO:0007010	Cytoskeleton organization	8.83E-04	ENSSSCG00000000114|ENSSSCG00000012767|ENSSSCG00000002965|ENSSSCG00000003577|ENSSSCG00000003342|ENSSSCG00000007469|ENSSSCG00000017251|ENSSSCG00000001660|ENSSSCG00000004657	*PICK1|HAUS7|ACTN4|WASF2|DVL1|PTPN1|SOX9|PTK7|CEP152*
Gene Ontology	GO:0048839	Inner ear development	8.99E-04	ENSSSCG00000001660|ENSSSCG00000016420|ENSSSCG00000017251|ENSSSCG00000003342	*PTK7|INSIG1|SOX9|DVL1*

Gene Ontology	GO:0110165	Cellular anatomical entity	9.02E-04	ENSSSCG00000000114|ENSSSCG00000009267|ENSSSCG00000009266|ENSSSCG00000009261|ENSSSCG00000002965|ENSSSCG00000003949|ENSSSCG00000023636|ENSSSCG00000003755|ENSSSCG00000011853|ENSSSCG00000017251|ENSSSCG00000012520|ENSSSCG00000021991|ENSSSCG00000007497|ENSSSCG00000002671|ENSSSCG00000001660|ENSSSCG00000004657|ENSSSCG00000008973|ENSSSCG00000015405|ENSSSCG00000007715|ENSSSCG00000006578|ENSSSCG00000017177|ENSSSCG00000000522|ENSSSCG00000014061|ENSSSCG00000026293|ENSSSCG00000007496|ENSSSCG00000016420|ENSSSCG00000003577|ENSSSCG00000005707|ENSSSCG00000003342|ENSSSCG00000012842|ENSSSCG00000023472|ENSSSCG00000015524|ENSSSCG00000023078|ENSSSCG00000023743|ENSSSCG00000023607|ENSSSCG00000017376|ENSSSCG00000006748|ENSSSCG00000014003|ENSSSCG00000030177|ENSSSCG00000015717|ENSSSCG00000006582|ENSSSCG00000002287|ENSSSCG00000028973|ENSSSCG00000003951|ENSSSCG00000017181|ENSSSCG00000016370|ENSSSCG00000016569|ENSSSCG00000001658|ENSSSCG00000016659|ENSSSCG00000006665|ENSSSCG00000012767|ENSSSCG00000005908|ENSSSCG00000020705|ENSSSCG00000021880|ENSSSCG00000018031|ENSSSCG00000009262|ENSSSCG00000016431|ENSSSCG00000023362|ENSSSCG00000015267|ENSSSCG00000008959|ENSSSCG00000015270|ENSSSCG00000016232|ENSSSCG00000007585|ENSSSCG00000017904|ENSSSCG00000012071|ENSSSCG00000007469|ENSSSCG00000008122|ENSSSCG00000003701	*PICK1|CSN3|ODAM|CSN2|ACTN4|CDC20|TMEM222|MCOLN2|RUBCN|SOX9|NA|NDUFV3|GCNT7|ATP2C2|PTK7|CEP152|NA|CD36|ABHD11|S100A4|ST6GALNAC2|NAP1L1|THOC3|STX5|RTF2|INSIG1|WASF2|FIBCD1|DVL1|RPLP2|FMO3|FAM20B|WDR4|CLDN6|CYP4F22|MEOX1|MIER2|EBNA1BP2|C1QL2|S100A14|MPP5|PHACTR4|C1orf210|CYGB|SEPTIN2|SMO|MRPL2|KLC4|SF3B4|HAUS7|SLC52A2|MAP3K8|MXRA8|ZNF287|CSN1S1|GALNTL5|RHBDF2|FMO2|CXCL2|FMOD|MRPL44|ACTB|ENO3|NA|PTPN1|SNRPD1*
Gene Ontology	GO:0048468	Cell development	1.63E-03	ENSSSCG00000016569|ENSSSCG00000016431|ENSSSCG00000002965|ENSSSCG00000003949|ENSSSCG00000002287|ENSSSCG00000003577|ENSSSCG00000003342|ENSSSCG00000012520|ENSSSCG00000017251|ENSSSCG00000001660|ENSSSCG00000006748|ENSSSCG00000021880	*SMO|GALNTL5|ACTN4|CDC20|MPP5|WASF2|DVL1|NA|SOX9|PTK7|TSPAN2|MXRA8*

Gene Ontology	GO:0043583	Ear development	1.71E-03	ENSSSCG00000001660|ENSSSCG00000016420|ENSSSCG00000017251|ENSSSCG00000003342	*PTK7|INSIG1|SOX9|DVL1*
Gene Ontology	GO:2000027	Regulation of animal organ morphogenesis	1.71E-03	ENSSSCG00000001660|ENSSSCG00000016569|ENSSSCG00000017251|ENSSSCG00000003342	*PTK7|SMO|SOX9|DVL1*
Gene Ontology	GO:0048562	Embryonic organ morphogenesis	1.88E-03	ENSSSCG00000001660|ENSSSCG00000016420|ENSSSCG00000017251|ENSSSCG00000003342|ENSSSCG00000016569	*PTK7|INSIG1|SOX9|DVL1|SMO*
Gene Ontology	GO:0045216	Cell-cell junction organization	1.93E-03	ENSSSCG00000002287|ENSSSCG00000023743|ENSSSCG00000002965	*MPP5|CLDN6|ACTN4*
Gene Ontology	GO:0043209	Myelin sheath	2.01E-03	ENSSSCG00000002287|ENSSSCG00000006748|ENSSSCG00000007585|ENSSSCG00000016370	*MPP5|TSPAN2|ACTB|SEPTIN2*
Gene Ontology	GO:0060284	Regulation of cell development	2.11E-03	ENSSSCG00000016569|ENSSSCG00000002965|ENSSSCG00000003949|ENSSSCG00000017251|ENSSSCG00000012520|ENSSSCG00000003342|ENSSSCG00000001660	*SMO|ACTN4|CDC20|SOX9|NA|DVL1|PTK7*
Gene Ontology	GO:0051962	Positive regulation of nervous system development	2.34E-03	ENSSSCG00000001660|ENSSSCG00000012520|ENSSSCG00000016569|ENSSSCG00000003342|ENSSSCG00000003949	*PTK7|NA|SMO|DVL1|CDC20*
Gene Ontology	GO:0032879	Regulation of localization	2.53E-03	ENSSSCG00000016569|ENSSSCG00000009261|ENSSSCG00000002965|ENSSSCG00000023362|ENSSSCG00000006582|ENSSSCG00000016420|ENSSSCG00000011853|ENSSSCG00000003342|ENSSSCG00000016370|ENSSSCG00000015524|ENSSSCG00000007469|ENSSSCG00000017251|ENSSSCG00000002671	*SMO|CSN2|ACTN4|RHBDF2|S100A14|INSIG1|RUBCN|DVL1|SEPTIN2|FAM20B|PTPN1|SOX9|ATP2C2*
Gene Ontology	GO:0006984	ER-nucleus signaling pathway	2.63E-03	ENSSSCG00000016420|ENSSSCG00000007469	*INSIG1|PTPN1*
Gene Ontology	GO:1905276	Regulation of epithelial tube formation	2.63E-03	ENSSSCG00000001660|ENSSSCG00000003342	*PTK7|DVL1*
Gene Ontology	GO:0060026	Convergent extension	2.63E-03	ENSSSCG00000001660|ENSSSCG00000003342	*PTK7|DVL1*
Gene Ontology	GO:0031346	Positive regulation of cell projection organization	2.91E-03	ENSSSCG00000001660|ENSSSCG00000012520|ENSSSCG00000003577|ENSSSCG00000003342	*PTK7|NA|WASF2|DVL1*
Gene Ontology	GO:0050821	Protein stabilization	3.15E-03	ENSSSCG00000009267|ENSSSCG00000016569|ENSSSCG00000003342	*CSN3|SMO|DVL1*

Gene Ontology	GO:0010720	Positive regulation of cell development	3.20E-03	ENSSSCG00000001660|ENSSSCG00000012520|ENSSSCG00000016569|ENSSSCG00000003342|ENSSSCG00000003949	*PTK7|NA|SMO|DVL1|CDC20*
Gene Ontology	GO:0005911	Cell-cell junction	3.36E-03	ENSSSCG00000001660|ENSSSCG00000003577|ENSSSCG00000023743|ENSSSCG00000002965|ENSSSCG00000012071	*PTK7|WASF2|CLDN6|ACTN4|NA*
Gene Ontology	GO:0051668	Localization within membrane	3.48E-03	ENSSSCG00000016420|ENSSSCG00000003342	*INSIG1|DVL1*
Gene Ontology	GO:0060429	Epithelium development	3.83E-03	ENSSSCG00000016569|ENSSSCG00000002287|ENSSSCG00000003342|ENSSSCG00000004657|ENSSSCG00000017251|ENSSSCG00000002671|ENSSSCG00000001660|ENSSSCG00000017376	*SMO|MPP5|DVL1|CEP152|SOX9|ATP2C2|PTK7|MEOX1*
Gene Ontology	GO:0030100	Regulation of endocytosis	3.90E-03	ENSSSCG00000011853|ENSSSCG00000007469|ENSSSCG00000002965	*RUBCN|PTPN1|ACTN4*
Gene Ontology	GO:0048732	Gland development	4.26E-03	ENSSSCG00000009267|ENSSSCG00000016569|ENSSSCG00000009261|ENSSSCG00000017251|ENSSSCG00000002671	*CSN3|SMO|CSN2|SOX9|ATP2C2*
Gene Ontology	GO:0060071	Wnt signaling pathway, planar cell polarity pathway	4.44E-03	ENSSSCG00000001660|ENSSSCG00000003342	*PTK7|DVL1*
Gene Ontology	GO:0014002	Astrocyte development	4.44E-03	ENSSSCG00000006748|ENSSSCG00000016569	*TSPAN2|SMO*
Gene Ontology	GO:0090175	Regulation of establishment of planar polarity	4.44E-03	ENSSSCG00000001660|ENSSSCG00000003342	*PTK7|DVL1*
Gene Ontology	GO:0071300	Cellular response to retinoic acid	4.44E-03	ENSSSCG00000001660|ENSSSCG00000017251	*PTK7|SOX9*
Gene Ontology	GO:0005856	Cytoskeleton	4.51E-03	ENSSSCG00000002965|ENSSSCG00000006582|ENSSSCG00000016370|ENSSSCG00000003577|ENSSSCG00000003342|ENSSSCG00000007585|ENSSSCG00000004657|ENSSSCG00000001659|ENSSSCG00000003949|ENSSSCG00000012767	*ACTN4|S100A14|SEPTIN2|WASF2|DVL1|ACTB|CEP152|KLC4|CDC20|HAUS7*
Gene Ontology	GO:0006996	Organelle organization	4.69E-03	ENSSSCG00000000114|ENSSSCG00000012767|ENSSSCG00000000522|ENSSSCG00000002965|ENSSSCG00000003949|ENSSSCG00000026293|ENSSSCG00000011853|ENSSSCG00000016420|ENSSSCG00000003577|ENSSSCG00000003342|ENSSSCG00000015524|ENSSSCG00000007469|ENSSSCG00000017251|ENSSSCG00000001660|ENSSSCG00000004657|ENSSSCG00000003701	*PICK1|HAUS7|NAP1L1|ACTN4|CDC20|STX5|RUBCN|INSIG1|WASF2|DVL1|FAM20B|PTPN1|SOX9|PTK7|CEP152|SNRPD1*
Gene Ontology	GO:0001838	Embryonic epithelial tube formation	4.75E-03	ENSSSCG00000001660|ENSSSCG00000017251|ENSSSCG00000003342	*PTK7|SOX9|DVL1*

Gene Ontology	GO:0071840	Cellular component organization or biogenesis	4.95E-03	ENSSSCG00000000114|ENSSSCG00000002965|ENSSSCG00000003949|ENSSSCG00000011853|ENSSSCG00000017251|ENSSSCG00000012520|ENSSSCG00000007469|ENSSSCG00000001660|ENSSSCG00000000522|ENSSSCG00000016420|ENSSSCG00000003577|ENSSSCG00000003342|ENSSSCG00000015524|ENSSSCG00000004657|ENSSSCG00000023743|ENSSSCG00000006748|ENSSSCG00000030177|ENSSSCG00000015717|ENSSSCG00000002287|ENSSSCG00000012767|ENSSSCG00000015270|ENSSSCG00000026293|ENSSSCG00000003701	*PICK1|ACTN4|CDC20|RUBCN|SOX9|NA|PTPN1|PTK7|NAP1L1|INSIG1|WASF2|DVL1|FAM20B|CEP152|CLDN6|TSPAN2|EBNA1BP2|C1QL2|MPP5|HAUS7|FMOD|STX5|SNRPD1*
Gene Ontology	GO:0051049	Regulation of transport	5.12E-03	ENSSSCG00000016370|ENSSSCG00000009261|ENSSSCG00000002965|ENSSSCG00000023362|ENSSSCG00000016420|ENSSSCG00000011853|ENSSSCG00000016569|ENSSSCG00000015524|ENSSSCG00000007469|ENSSSCG00000002671	*SEPTIN2|CSN2|ACTN4|RHBDF2|INSIG1|RUBCN|SMO|FAM20B|PTPN1|ATP2C2*
Gene Ontology	GO:0072175	Epithelial tube formation	5.22E-03	ENSSSCG00000001660|ENSSSCG00000017251|ENSSSCG00000003342	*PTK7|SOX9|DVL1*
Gene Ontology	GO:0051960	Regulation of nervous system development	5.26E-03	ENSSSCG00000016569|ENSSSCG00000003949|ENSSSCG00000017251|ENSSSCG00000012520|ENSSSCG00000003342|ENSSSCG00000001660	*SMO|CDC20|SOX9|NA|DVL1|PTK7*
Gene Ontology	GO:0051179	Localization	5.30E-03	ENSSSCG00000000114|ENSSSCG00000009267|ENSSSCG00000009261|ENSSSCG00000002965|ENSSSCG00000009262|ENSSSCG00000011853|ENSSSCG00000017251|ENSSSCG00000007469|ENSSSCG00000002671|ENSSSCG00000001660|ENSSSCG00000014061|ENSSSCG00000016420|ENSSSCG00000003577|ENSSSCG00000003342|ENSSSCG00000015524|ENSSSCG00000006582|ENSSSCG00000002287|ENSSSCG00000017181|ENSSSCG00000005908|ENSSSCG00000016569|ENSSSCG00000023362|ENSSSCG00000008959|ENSSSCG00000016370|ENSSSCG00000026293	*PICK1|CSN3|CSN2|ACTN4|CSN1S1|RUBCN|SOX9|PTPN1|ATP2C2|PTK7|THOC3|INSIG1|WASF2|DVL1|FAM20B|S100A14|MPP5|CYGB|SLC52A2|SMO|RHBDF2|CXCL2|SEPTIN2|STX5*
Gene Ontology	GO:0090596	Sensory organ morphogenesis	5.50E-03	ENSSSCG00000001660|ENSSSCG00000016420|ENSSSCG00000017251|ENSSSCG00000003342	*PTK7|INSIG1|SOX9|DVL1*
Gene Ontology	GO:0072077	Renal vesicle morphogenesis	5.51E-03	ENSSSCG00000016569|ENSSSCG00000017251	*SMO|SOX9*

Gene Ontology	GO:0120193	Tight junction organization	5.51E-03	ENSSSCG00000002287|ENSSSCG00000002965	*MPP5|ACTN4*
Gene Ontology	GO:0002053	Positive regulation of mesenchymal cell proliferation	5.51E-03	ENSSSCG00000016569|ENSSSCG00000017251	*SMO|SOX9*
Gene Ontology	GO:0070830	Bicellular tight junction assembly	5.51E-03	ENSSSCG00000002287|ENSSSCG00000002965	*MPP5|ACTN4*
Gene Ontology	GO:0120192	Tight junction assembly	5.51E-03	ENSSSCG00000002287|ENSSSCG00000002965	*MPP5|ACTN4*
Gene Ontology	GO:0001736	Establishment of planar polarity	5.51E-03	ENSSSCG00000001660|ENSSSCG00000003342	*PTK7|DVL1*
Gene Ontology	GO:0072087	Renal vesicle development	5.51E-03	ENSSSCG00000016569|ENSSSCG00000017251	*SMO|SOX9*
Gene Ontology	GO:0007595	Lactation	5.51E-03	ENSSSCG00000009267|ENSSSCG00000009261	*CSN3|CSN2*
Gene Ontology	GO:0007164	Establishment of tissue polarity	5.51E-03	ENSSSCG00000001660|ENSSSCG00000003342	*PTK7|DVL1*
Gene Ontology	GO:0016043	Cellular component organization	5.61E-03	ENSSSCG00000000114|ENSSSCG00000002965|ENSSSCG00000003949|ENSSSCG00000011853|ENSSSCG00000017251|ENSSSCG00000012520|ENSSSCG00000007469|ENSSSCG00000001660|ENSSSCG00000000522|ENSSSCG00000016420|ENSSSCG00000003577|ENSSSCG00000003342|ENSSSCG00000015524|ENSSSCG00000004657|ENSSSCG00000023743|ENSSSCG00000006748|ENSSSCG00000015717|ENSSSCG00000002287|ENSSSCG00000012767|ENSSSCG00000015270|ENSSSCG00000026293|ENSSSCG00000003701	*PICK1|ACTN4|CDC20|RUBCN|SOX9|NA|PTPN1|PTK7|NAP1L1|INSIG1|WASF2|DVL1|FAM20B|CEP152|CLDN6|TSPAN2|C1QL2|MPP5|HAUS7|FMOD|STX5|SNRPD1*
Gene Ontology	GO:0098657	Import into cell	5.79E-03	ENSSSCG00000007469|ENSSSCG00000011853|ENSSSCG00000016370|ENSSSCG00000003577|ENSSSCG00000002965	*PTPN1|RUBCN|SEPTIN2|WASF2|ACTN4*
Gene Ontology	GO:0048568	Embryonic organ development	6.29E-03	ENSSSCG00000001660|ENSSSCG00000016420|ENSSSCG00000017251|ENSSSCG00000003342|ENSSSCG00000016569	*PTK7|INSIG1|SOX9|DVL1|SMO*
Gene Ontology	GO:0060485	Mesenchyme development	6.50E-03	ENSSSCG00000001660|ENSSSCG00000017376|ENSSSCG00000016569|ENSSSCG00000017251	*PTK7|MEOX1|SMO|SOX9*
Gene Ontology	GO:0030857	Negative regulation of epithelial cell differentiation	6.68E-03	ENSSSCG00000016569|ENSSSCG00000017251	*SMO|SOX9*
Gene Ontology	GO:0005689	U12-type spliceosomal complex	6.68E-03	ENSSSCG00000006665|ENSSSCG00000003701	*SF3B4|SNRPD1*
Gene Ontology	GO:0060170	Ciliary membrane	6.68E-03	ENSSSCG00000016569|ENSSSCG00000016370	*SMO|SEPTIN2*
Gene Ontology	GO:0010464	Regulation of mesenchymal cell proliferation	6.68E-03	ENSSSCG00000016569|ENSSSCG00000017251	*SMO|SOX9*

Gene Ontology	GO:0006414	Translational elongation	6.68E-03	ENSSSCG00000016232|ENSSSCG00000012842	*MRPL44|RPLP2*
Gene Ontology	GO:0001738	Morphogenesis of a polarized epithelium	6.68E-03	ENSSSCG00000001660|ENSSSCG00000003342	*PTK7|DVL1*
Gene Ontology	GO:0090190	Positive regulation of branching involved in ureteric bud morphogenesis	6.68E-03	ENSSSCG00000016569|ENSSSCG00000017251	*SMO|SOX9*
Gene Ontology	GO:0031018	Endocrine pancreas development	6.68E-03	ENSSSCG00000016569|ENSSSCG00000017251	*SMO|SOX9*
Gene Ontology	GO:0035148	Tube formation	6.78E-03	ENSSSCG00000001660|ENSSSCG00000017251|ENSSSCG00000003342	*PTK7|SOX9|DVL1*
Gene Ontology	GO:0016331	Morphogenesis of embryonic epithelium	6.78E-03	ENSSSCG00000001660|ENSSSCG00000017251|ENSSSCG00000003342	*PTK7|SOX9|DVL1*
Gene Ontology	GO:0050808	Synapse organization	6.78E-03	ENSSSCG00000012520|ENSSSCG00000003342|ENSSSCG00000003949	*NA|DVL1|CDC20*
Gene Ontology	GO:0009896	Positive regulation of catabolic process	7.10E-03	ENSSSCG00000026293|ENSSSCG00000017251|ENSSSCG00000007469|ENSSSCG00000003701|ENSSSCG00000003342	*STX5|SOX9|PTPN1|SNRPD1|DVL1*
Gene Ontology	GO:0050769	Positive regulation of neurogenesis	7.23E-03	ENSSSCG00000001660|ENSSSCG00000012520|ENSSSCG00000016569|ENSSSCG00000003342	*PTK7|NA|SMO|DVL1*
Gene Ontology	GO:0008104	Protein localization	7.31E-03	ENSSSCG00000016569|ENSSSCG00000002965|ENSSSCG00000023362|ENSSSCG00000014061|ENSSSCG00000002287|ENSSSCG00000016370|ENSSSCG00000026293|ENSSSCG00000003342|ENSSSCG00000015524|ENSSSCG00000007469|ENSSSCG00000017251	*SMO|ACTN4|RHBDF2|THOC3|MPP5|SEPTIN2|STX5|DVL1|FAM20B|PTPN1|SOX9*
Gene Ontology	GO:0021915	Neural tube development	7.35E-03	ENSSSCG00000001660|ENSSSCG00000016569|ENSSSCG00000003342	*PTK7|SMO|DVL1*
Gene Ontology	GO:0060348	Bone development	7.35E-03	ENSSSCG00000016420|ENSSSCG00000003577|ENSSSCG00000017251	*INSIG1|WASF2|SOX9*
Gene Ontology	GO:0034330	Cell junction organization	7.95E-03	ENSSSCG00000002287|ENSSSCG00000023743|ENSSSCG00000002965	*MPP5|CLDN6|ACTN4*
Gene Ontology	GO:0003401	Axis elongation	7.96E-03	ENSSSCG00000001660|ENSSSCG00000017251	*PTK7|SOX9*
Gene Ontology	GO:0061213	Positive regulation of mesonephros development	7.96E-03	ENSSSCG00000016569|ENSSSCG00000017251	*SMO|SOX9*
Gene Ontology	GO:2000826	Regulation of heart morphogenesis	7.96E-03	ENSSSCG00000016569|ENSSSCG00000017251	*SMO|SOX9*
Gene Ontology	GO:0048873	Homeostasis of number of cells within a tissue	7.96E-03	ENSSSCG00000016569|ENSSSCG00000017251	*SMO|SOX9*

Gene Ontology	GO:0072273	Metanephric nephron morphogenesis	7.96E-03	ENSSSCG00000016569|ENSSSCG00000017251	*SMO|SOX9*
Gene Ontology	GO:0035567	Non-canonical Wnt signaling pathway	7.96E-03	ENSSSCG00000001660|ENSSSCG00000003342	*PTK7|DVL1*
Gene Ontology	GO:0022008	Neurogenesis	8.12E-03	ENSSSCG00000016569|ENSSSCG00000002287|ENSSSCG00000003342|ENSSSCG00000012520|ENSSSCG00000017251|ENSSSCG00000001660|ENSSSCG00000006748|ENSSSCG00000021880	*SMO|MPP5|DVL1|NA|SOX9|PTK7|TSPAN2|MXRA8*
Gene Ontology	GO:0033043	Regulation of organelle organization	8.50E-03	ENSSSCG00000000114|ENSSSCG00000003949|ENSSSCG00000026293|ENSSSCG00000011853|ENSSSCG00000016420|ENSSSCG00000003577|ENSSSCG00000015524|ENSSSCG00000003701	*PICK1|CDC20|STX5|RUBCN|INSIG1|WASF2|FAM20B|SNRPD1*
Gene Ontology	GO:0010976	Positive regulation of neuron projection development	8.58E-03	ENSSSCG00000001660|ENSSSCG00000012520|ENSSSCG00000003342	*PTK7|NA|DVL1*
Gene Ontology	GO:0030036	Actin cytoskeleton organization	9.26E-03	ENSSSCG00000000114|ENSSSCG00000001660|ENSSSCG00000003577|ENSSSCG00000002965|ENSSSCG00000007469	*PICK1|PTK7|WASF2|ACTN4|PTPN1*
Gene Ontology	GO:0030054	Cell junction	9.31E-03	ENSSSCG00000002965|ENSSSCG00000006582|ENSSSCG00000012071|ENSSSCG00000003577|ENSSSCG00000007585|ENSSSCG00000023743|ENSSSCG00000001660	*ACTN4|S100A14|NA|WASF2|ACTB|CLDN6|PTK7*
Gene Ontology	GO:0072210	Metanephric nephron development	9.33E-03	ENSSSCG00000016569|ENSSSCG00000017251	*SMO|SOX9*
Gene Ontology	GO:0048709	Oligodendrocyte differentiation	9.33E-03	ENSSSCG00000006748|ENSSSCG00000017251	*TSPAN2|SOX9*
Gene Ontology	GO:0043297	Apical junction assembly	9.33E-03	ENSSSCG00000002287|ENSSSCG00000002965	*MPP5|ACTN4*
Gene Ontology	GO:0005634	Nucleus	1.02E-02	ENSSSCG00000009266|ENSSSCG00000002965|ENSSSCG00000003949|ENSSSCG00000026293|ENSSSCG00000011853|ENSSSCG00000017251|ENSSSCG00000021991|ENSSSCG00000000522|ENSSSCG00000014061|ENSSSCG00000006665|ENSSSCG00000007496|ENSSSCG00000006578|ENSSSCG00000015524|ENSSSCG00000023078|ENSSSCG00000004657|ENSSSCG00000017376|ENSSSCG00000014003|ENSSSCG00000030177|ENSSSCG00000006582|ENSSSCG00000002287|ENSSSCG00000028973|ENSSSCG00000012767|ENSSSCG00000018031|ENSSSCG00000016370|ENSSSCG00000016232|ENSSSCG00000003701	*ODAM|ACTN4|CDC20|STX5|RUBCN|SOX9|NDUFV3|NAP1L1|THOC3|SF3B4|RTF2|S100A4|FAM20B|WDR4|CEP152|MEOX1|MIER2|EBNA1BP2|S100A14|MPP5|PHACTR4|HAUS7|ZNF287|SEPTIN2|MRPL44|SNRPD1*

Gene Ontology	GO:0032386	Regulation of intracellular transport	1.02E-02	ENSSSCG00000016420|ENSSSCG00000015524|ENSSSCG00000007469|ENSSSCG00000016569	*INSIG1|FAM20B|PTPN1|SMO*
Gene Ontology	GO:0009790	Embryo development	1.05E-02	ENSSSCG00000016569|ENSSSCG00000015780|ENSSSCG00000016420|ENSSSCG00000003342|ENSSSCG00000017251|ENSSSCG00000001660|ENSSSCG00000017376	*SMO|STOX2|INSIG1|DVL1|SOX9|PTK7|MEOX1*
Gene Ontology	GO:0002009	Morphogenesis of an epithelium	1.07E-02	ENSSSCG00000001660|ENSSSCG00000016569|ENSSSCG00000017251|ENSSSCG00000003342|ENSSSCG00000002287	*PTK7|SMO|SOX9|DVL1|MPP5*
Gene Ontology	GO:0061515	Myeloid cell development	1.08E-02	ENSSSCG00000006748|ENSSSCG00000003577	*TSPAN2|WASF2*
Gene Ontology	GO:1905332	Positive regulation of morphogenesis of an epithelium	1.08E-02	ENSSSCG00000016569|ENSSSCG00000017251	*SMO|SOX9*
Gene Ontology	GO:0061180	Mammary gland epithelium development	1.08E-02	ENSSSCG00000016569|ENSSSCG00000002671	*SMO|ATP2C2*
Gene Ontology	GO:0090189	Regulation of branching involved in ureteric bud morphogenesis	1.08E-02	ENSSSCG00000016569|ENSSSCG00000017251	*SMO|SOX9*
Gene Ontology	GO:0043229	Intracellular organelle	1.10E-02	ENSSSCG00000009266|ENSSSCG00000002965|ENSSSCG00000003949|ENSSSCG00000026293|ENSSSCG00000011853|ENSSSCG00000017251|ENSSSCG00000007469|ENSSSCG00000007497|ENSSSCG00000002671|ENSSSCG00000008973|ENSSSCG00000007715|ENSSSCG00000006578|ENSSSCG00000017177|ENSSSCG00000000522|ENSSSCG00000014061|ENSSSCG00000006665|ENSSSCG00000007496|ENSSSCG00000016420|ENSSSCG00000003577|ENSSSCG00000003342|ENSSSCG00000012842|ENSSSCG00000015524|ENSSSCG00000023078|ENSSSCG00000004657|ENSSSCG00000017376|ENSSSCG00000014003|ENSSSCG00000030177|ENSSSCG00000006582|ENSSSCG00000002287|ENSSSCG00000028973|ENSSSCG00000016569|ENSSSCG00000001658|ENSSSCG00000001659|ENSSSCG00000012767|ENSSSCG00000018031|ENSSSCG00000016370|ENSSSCG00000016232|ENSSSCG00000007585|ENSSSCG00000021991|ENSSSCG00000003701	*ODAM|ACTN4|CDC20|STX5|RUBCN|SOX9|PTPN1|GCNT7|ATP2C2|NA|ABHD11|S100A4|ST6GALNAC2|NAP1L1|THOC3|SF3B4|RTF2|INSIG1|WASF2|DVL1|RPLP2|FAM20B|WDR4|CEP152|MEOX1|MIER2|EBNA1BP2|S100A14|MPP5|PHACTR4|SMO|MRPL2|KLC4|HAUS7|ZNF287|SEPTIN2|ACTB|NDUFV3|SNRPD1*

Gene Ontology	GO:0097435	Supramolecular fiber organization	1.11E-02	ENSSSCG00000000114|ENSSSCG00000003577|ENSSSCG00000002965|ENSSSCG00000003342|ENSSSCG00000015270	*PICK1|WASF2|ACTN4|DVL1|FMOD*
Gene Ontology	GO:0032880	Regulation of protein localization	1.11E-02	ENSSSCG00000016569|ENSSSCG00000023362|ENSSSCG00000016370|ENSSSCG00000003342|ENSSSCG00000015524|ENSSSCG00000007469	*SMO|RHBDF2|SEPTIN2|DVL1|FAM20B|PTPN1*
Gene Ontology	GO:0060070	Canonical Wnt signaling pathway	1.14E-02	ENSSSCG00000001660|ENSSSCG00000017251|ENSSSCG00000003342	*PTK7|SOX9|DVL1*
Gene Ontology	GO:0050767	Regulation of neurogenesis	1.14E-02	ENSSSCG00000001660|ENSSSCG00000012520|ENSSSCG00000016569|ENSSSCG00000017251|ENSSSCG00000003342	*PTK7|NA|SMO|SOX9|DVL1*
Gene Ontology	GO:0022607	Cellular component assembly	1.17E-02	ENSSSCG00000000114|ENSSSCG00000002965|ENSSSCG00000000522|ENSSSCG00000002287|ENSSSCG00000003577|ENSSSCG00000017251|ENSSSCG00000012520|ENSSSCG00000004657|ENSSSCG00000012767|ENSSSCG00000003701|ENSSSCG00000015717	*PICK1|ACTN4|NAP1L1|MPP5|WASF2|SOX9|NA|CEP152|HAUS7|SNRPD1|C1QL2*
Gene Ontology	GO:1903827	Regulation of cellular protein localization	1.22E-02	ENSSSCG00000015524|ENSSSCG00000007469|ENSSSCG00000003342|ENSSSCG00000016569	*FAM20B|PTPN1|DVL1|SMO*
Gene Ontology	GO:0044089	Positive regulation of cellular component biogenesis	1.22E-02	ENSSSCG00000000114|ENSSSCG00000012520|ENSSSCG00000003577|ENSSSCG00000017251	*PICK1|NA|WASF2|SOX9*
Gene Ontology	GO:0000139	Golgi membrane	1.22E-02	ENSSSCG00000007497|ENSSSCG00000026293|ENSSSCG00000017177|ENSSSCG00000002671	*GCNT7|STX5|ST6GALNAC2|ATP2C2*

Gene Ontology	GO:0043226	Organelle	1.23E-02	ENSSSCG00000009266|ENSSSCG00000002965|ENSSSCG00000003949|ENSSSCG00000026293|ENSSSCG00000011853|ENSSSCG00000017251|ENSSSCG00000012520|ENSSSCG00000021991|ENSSSCG00000007497|ENSSSCG00000002671|ENSSSCG00000008973|ENSSSCG00000007715|ENSSSCG00000006578|ENSSSCG00000017177|ENSSSCG00000000522|ENSSSCG00000014061|ENSSSCG00000006665|ENSSSCG00000007496|ENSSSCG00000016420|ENSSSCG00000003577|ENSSSCG00000003342|ENSSSCG00000012842|ENSSSCG00000023472|ENSSSCG00000015524|ENSSSCG00000023078|ENSSSCG00000004657|ENSSSCG00000017376|ENSSSCG00000014003|ENSSSCG00000030177|ENSSSCG00000006582|ENSSSCG00000002287|ENSSSCG00000028973|ENSSSCG00000016569|ENSSSCG00000001658|ENSSSCG00000001659|ENSSSCG00000012767|ENSSSCG00000021880|ENSSSCG00000018031|ENSSSCG00000023362|ENSSSCG00000016370|ENSSSCG00000016232|ENSSSCG00000007585|ENSSSCG00000007469|ENSSSCG00000003701	*ODAM|ACTN4|CDC20|STX5|RUBCN|SOX9|NA|NDUFV3|GCNT7|ATP2C2|NA|ABHD11|S100A4|ST6GALNAC2|NAP1L1|THOC3|SF3B4|RTF2|INSIG1|WASF2|DVL1|RPLP2|FMO3|FAM20B|WDR4|CEP152|MEOX1|MIER2|EBNA1BP2|S100A14|MPP5|PHACTR4|SMO|MRPL2|KLC4|HAUS7|ZNF287|RHBDF2|SEPTIN2|MRPL44|ACTB|PTPN1|SNRPD1*
Gene Ontology	GO:0010463	Mesenchymal cell proliferation	1.24E-02	ENSSSCG00000016569|ENSSSCG00000017251	*SMO|SOX9*
Gene Ontology	GO:0030863	Cortical cytoskeleton	1.24E-02	ENSSSCG00000002965|ENSSSCG00000007585	*ACTN4|ACTB*
Gene Ontology	GO:0090184	Positive regulation of kidney development	1.24E-02	ENSSSCG00000016569|ENSSSCG00000017251	*SMO|SOX9*
Gene Ontology	GO:0003338	Metanephros morphogenesis	1.24E-02	ENSSSCG00000016569|ENSSSCG00000017251	*SMO|SOX9*
Gene Ontology	GO:0061217	Regulation of mesonephros development	1.24E-02	ENSSSCG00000016569|ENSSSCG00000017251	*SMO|SOX9*
Gene Ontology	GO:0044085	Cellular component biogenesis	1.31E-02	ENSSSCG00000000114|ENSSSCG00000002965|ENSSSCG00000000522|ENSSSCG00000002287|ENSSSCG00000003577|ENSSSCG00000017251|ENSSSCG00000012520|ENSSSCG00000004657|ENSSSCG00000012767|ENSSSCG00000003701|ENSSSCG00000030177|ENSSSCG00000015717	*PICK1|ACTN4|NAP1L1|MPP5|WASF2|SOX9|NA|CEP152|HAUS7|SNRPD1|EBNA1BP2|C1QL2*

Gene Ontology	GO:0048731	System development	1.32E-02	ENSSSCG00000009267|ENSSSCG00000009266|ENSSSCG00000009261|ENSSSCG00000003949|ENSSSCG00000002287|ENSSSCG00000016420|ENSSSCG00000003577|ENSSSCG00000003342|ENSSSCG00000015780|ENSSSCG00000012520|ENSSSCG00000016569|ENSSSCG00000017251|ENSSSCG00000002671|ENSSSCG00000001660|ENSSSCG00000017376|ENSSSCG00000006748|ENSSSCG00000021880	*CSN3|ODAM|CSN2|CDC20|MPP5|INSIG1|WASF2|DVL1|STOX2|NA|SMO|SOX9|ATP2C2|PTK7|MEOX1|TSPAN2|MXRA8*
Gene Ontology	GO:0060560	Developmental growth involved in morphogenesis	1.38E-02	ENSSSCG00000001660|ENSSSCG00000017251|ENSSSCG00000003342	*PTK7|SOX9|DVL1*
Gene Ontology	GO:0031647	Regulation of protein stability	1.38E-02	ENSSSCG00000009267|ENSSSCG00000016569|ENSSSCG00000003342	*CSN3|SMO|DVL1*
Gene Ontology	GO:0048856	Anatomical structure development	1.38E-02	ENSSSCG00000009267|ENSSSCG00000009266|ENSSSCG00000009261|ENSSSCG00000016431|ENSSSCG00000002965|ENSSSCG00000003949|ENSSSCG00000002287|ENSSSCG00000016420|ENSSSCG00000003577|ENSSSCG00000003342|ENSSSCG00000015780|ENSSSCG00000012520|ENSSSCG00000016569|ENSSSCG00000004657|ENSSSCG00000017251|ENSSSCG00000002671|ENSSSCG00000001660|ENSSSCG00000017376|ENSSSCG00000006748|ENSSSCG00000021880	*CSN3|ODAM|CSN2|GALNTL5|ACTN4|CDC20|MPP5|INSIG1|WASF2|DVL1|STOX2|NA|SMO|CEP152|SOX9|ATP2C2|PTK7|MEOX1|TSPAN2|MXRA8*
Gene Ontology	GO:0034613	Cellular protein localization	1.39E-02	ENSSSCG00000016569|ENSSSCG00000014061|ENSSSCG00000002287|ENSSSCG00000026293|ENSSSCG00000003342|ENSSSCG00000015524|ENSSSCG00000007469|ENSSSCG00000017251	*SMO|THOC3|MPP5|STX5|DVL1|FAM20B|PTPN1|SOX9*
Gene Ontology	GO:0060341	Regulation of cellular localization	1.39E-02	ENSSSCG00000016420|ENSSSCG00000015524|ENSSSCG00000007469|ENSSSCG00000003342|ENSSSCG00000016569	*INSIG1|FAM20B|PTPN1|DVL1|SMO*

Gene Ontology	GO:0006810	Transport	1.40E-02	ENSSSCG00000000114|ENSSSCG00000009267|ENSSSCG00000016569|ENSSSCG00000009261|ENSSSCG00000002965|ENSSSCG00000023362|ENSSSCG00000009262|ENSSSCG00000014061|ENSSSCG00000026293|ENSSSCG00000011853|ENSSSCG00000016420|ENSSSCG00000003577|ENSSSCG00000003342|ENSSSCG00000016370|ENSSSCG00000015524|ENSSSCG00000007469|ENSSSCG00000017181|ENSSSCG00000002671|ENSSSCG00000005908	*PICK1|CSN3|SMO|CSN2|ACTN4|RHBDF2|CSN1S1|THOC3|STX5|RUBCN|INSIG1|WASF2|DVL1|SEPTIN2|FAM20B|PTPN1|CYGB|ATP2C2|SLC52A2*
Gene Ontology	GO:0016477	Cell migration	1.40E-02	ENSSSCG00000016569|ENSSSCG00000002965|ENSSSCG00000006582|ENSSSCG00000008959|ENSSSCG00000003577|ENSSSCG00000017251|ENSSSCG00000001660	*SMO|ACTN4|S100A14|CXCL2|WASF2|SOX9|PTK7*
Gene Ontology	GO:0007043	Cell-cell junction assembly	1.40E-02	ENSSSCG00000002287|ENSSSCG00000002965	*MPP5|ACTN4*
Gene Ontology	GO:0050661	NADP binding	1.40E-02	ENSSSCG00000023472|ENSSSCG00000015267	*FMO3|FMO2*
Gene Ontology	GO:0007173	Epidermal growth factor receptor signaling pathway	1.40E-02	ENSSSCG00000023362|ENSSSCG00000017251	*RHBDF2|SOX9*
Gene Ontology	GO:0032543	Mitochondrial translation	1.40E-02	ENSSSCG00000001658|ENSSSCG00000016232	*MRPL2|MRPL44*
Gene Ontology	GO:0042552	Myelination	1.40E-02	ENSSSCG00000002287|ENSSSCG00000006748	*MPP5|TSPAN2*
Gene Ontology	GO:0009894	Regulation of catabolic process	1.42E-02	ENSSSCG00000011853|ENSSSCG00000026293|ENSSSCG00000017251|ENSSSCG00000007469|ENSSSCG00000003342|ENSSSCG00000003701	*RUBCN|STX5|SOX9|PTPN1|DVL1|SNRPD1*
Gene Ontology	GO:0060562	Epithelial tube morphogenesis	1.45E-02	ENSSSCG00000001660|ENSSSCG00000016569|ENSSSCG00000017251|ENSSSCG00000003342	*PTK7|SMO|SOX9|DVL1*
Gene Ontology	GO:0045732	Positive regulation of protein catabolic process	1.47E-02	ENSSSCG00000026293|ENSSSCG00000017251|ENSSSCG00000003342	*STX5|SOX9|DVL1*
Gene Ontology	GO:0030029	Actin filament-based process	1.49E-02	ENSSSCG00000000114|ENSSSCG00000001660|ENSSSCG00000003577|ENSSSCG00000002965|ENSSSCG00000007469	*PICK1|PTK7|WASF2|ACTN4|PTPN1*
Gene Ontology	GO:0060627	Regulation of vesicle-mediated transport	1.51E-02	ENSSSCG00000011853|ENSSSCG00000016420|ENSSSCG00000007469|ENSSSCG00000002965	*RUBCN|INSIG1|PTPN1|ACTN4*
Gene Ontology	GO:0120035	Regulation of plasma membrane bounded cell projection organization	1.51E-02	ENSSSCG00000001660|ENSSSCG00000012520|ENSSSCG00000003577|ENSSSCG00000003342	*PTK7|NA|WASF2|DVL1*

Gene Ontology	GO:0070727	Cellular macromolecule localization	1.53E-02	ENSSSCG00000016569|ENSSSCG00000014061|ENSSSCG00000002287|ENSSSCG00000026293|ENSSSCG00000003342|ENSSSCG00000015524|ENSSSCG00000007469|ENSSSCG00000017251	*SMO|THOC3|MPP5|STX5|DVL1|FAM20B|PTPN1|SOX9*
Gene Ontology	GO:0045597	Positive regulation of cell differentiation	1.54E-02	ENSSSCG00000016569|ENSSSCG00000003949|ENSSSCG00000017251|ENSSSCG00000012520|ENSSSCG00000003342|ENSSSCG00000001660	*SMO|CDC20|SOX9|NA|DVL1|PTK7*
Gene Ontology	GO:0031344	Regulation of cell projection organization	1.57E-02	ENSSSCG00000001660|ENSSSCG00000012520|ENSSSCG00000003577|ENSSSCG00000003342	*PTK7|NA|WASF2|DVL1*
Gene Ontology	GO:0030010	Establishment of cell polarity	1.58E-02	ENSSSCG00000001660|ENSSSCG00000002287	*PTK7|MPP5*
Gene Ontology	GO:0048813	Dendrite morphogenesis	1.58E-02	ENSSSCG00000012520|ENSSSCG00000003342	*NA|DVL1*
Gene Ontology	GO:0032526	Response to retinoic acid	1.58E-02	ENSSSCG00000001660|ENSSSCG00000017251	*PTK7|SOX9*
Gene Ontology	GO:0042379	Chemokine receptor binding	1.58E-02	ENSSSCG00000008959|ENSSSCG00000006582	*CXCL2|S100A14*
Gene Ontology	GO:0007589	Body fluid secretion	1.58E-02	ENSSSCG00000009267|ENSSSCG00000009261	*CSN3|CSN2*
Gene Ontology	GO:0002011	Morphogenesis of an epithelial sheet	1.58E-02	ENSSSCG00000002287|ENSSSCG00000003342	*MPP5|DVL1*
Gene Ontology	GO:0031016	Pancreas development	1.58E-02	ENSSSCG00000016569|ENSSSCG00000017251	*SMO|SOX9*
Gene Ontology	GO:0051234	Establishment of localization	1.62E-02	ENSSSCG00000000114|ENSSSCG00000009267|ENSSSCG00000016569|ENSSSCG00000009261|ENSSSCG00000002965|ENSSSCG00000023362|ENSSSCG00000009262|ENSSSCG00000014061|ENSSSCG00000026293|ENSSSCG00000011853|ENSSSCG00000016420|ENSSSCG00000003577|ENSSSCG00000003342|ENSSSCG00000016370|ENSSSCG00000015524|ENSSSCG00000007469|ENSSSCG00000017181|ENSSSCG00000002671|ENSSSCG00000005908	*PICK1|CSN3|SMO|CSN2|ACTN4|RHBDF2|CSN1S1|THOC3|STX5|RUBCN|INSIG1|WASF2|DVL1|SEPTIN2|FAM20B|PTPN1|CYGB|ATP2C2|SLC52A2*

<table>
<tr><td></td><td></td><td></td><td></td><td></td><td></td></tr>
<tr><td>Gene Ontology</td><td>GO:0071944</td><td>Cell periphery</td><td>1.65E-02</td><td>ENSSSCG00000000114|ENSSSCG00000008122|ENSSSCG00000002965|ENSSSCG00000023362|ENSSSCG00000006582|ENSSSCG00000012767|ENSSSCG00000002287|ENSSSCG00000016370|ENSSSCG00000003342|ENSSSCG00000007585|ENSSSCG00000016569|ENSSSCG00000007469|ENSSSCG00000023743|ENSSSCG00000002671|ENSSSCG00000012071|ENSSSCG00000001660|ENSSSCG00000006748|ENSSSCG00000005908</td><td>PICK1|ADRA2B|ACTN4|RHBDF2|S100A14|HAUS7|MPP5|SEPTIN2|DVL1|ACTB|SMO|PTPN1|CLDN6|ATP2C2|NA|PTK7|TSPAN2|SLC52A2</td></tr>
<tr><td>Gene Ontology</td><td>GO:0043227</td><td>Membrane-bounded organelle</td><td>1.69E-02</td><td>ENSSSCG00000009266|ENSSSCG00000002965|ENSSSCG00000003949|ENSSSCG00000026293|ENSSSCG00000011853|ENSSSCG00000017251|ENSSSCG00000012520|ENSSSCG00000021991|ENSSSCG00000007497|ENSSSCG00000002671|ENSSSCG00000008973|ENSSSCG00000007715|ENSSSCG00000006578|ENSSSCG00000017177|ENSSSCG00000000522|ENSSSCG00000014061|ENSSSCG00000006665|ENSSSCG00000007496|ENSSSCG00000016420|ENSSSCG00000003577|ENSSSCG00000003342|ENSSSCG00000023472|ENSSSCG00000015524|ENSSSCG00000023078|ENSSSCG00000004657|ENSSSCG00000017376|ENSSSCG00000014003|ENSSSCG00000030177|ENSSSCG00000006582|ENSSSCG00000002287|ENSSSCG00000028973|ENSSSCG00000016569|ENSSSCG00000001658|ENSSSCG00000012767|ENSSSCG00000021880|ENSSSCG00000018031|ENSSSCG00000023362|ENSSSCG00000016370|ENSSSCG00000016232|ENSSSCG00000007469|ENSSSCG00000003701</td><td>ODAM|ACTN4|CDC20|STX5|RUBCN|SOX9|NA|NDUFV3|GCNT7|ATP2C2|NA|ABHD11|S100A4|ST6GALNAC2|NAP1L1|THOC3|SF3B4|RTF2|INSIG1|WASF2|DVL1|FMO3|FAM20B|WDR4|CEP152|MEOX1|MIER2|EBNA1BP2|S100A14|MPP5|PHACTR4|SMO|MRPL2|HAUS7|MXRA8|ZNF287|SEPTIN2|MRPL44|PTPN1|SNRPD1</td></tr>
<tr><td>Gene Ontology</td><td>GO:1905114</td><td>Cell surface receptor signaling pathway involved in cell-cell signaling</td><td>1.70E-02</td><td>ENSSSCG00000001660|ENSSSCG00000016569|ENSSSCG00000017251|ENSSSCG00000003342</td><td>PTK7|SMO|SOX9|DVL1</td></tr>
<tr><td>Gene Ontology</td><td>GO:0006396</td><td>RNA processing</td><td>1.70E-02</td><td>ENSSSCG00000007494|ENSSSCG00000006665|ENSSSCG00000016232|ENSSSCG00000023078|ENSSSCG00000003701|ENSSSCG00000030177</td><td>CSTF1|SF3B4|MRPL44|WDR4|SNRPD1|EBNA1BP2</td></tr>
</table>

Gene Ontology	GO:0048598	Embryonic morphogenesis	1.73E-02	ENSSSCG00000001660|ENSSSCG00000016420|ENSSSCG00000017251|ENSSSCG00000003342|ENSSSCG00000016569	*PTK7|INSIG1|SOX9|DVL1|SMO*
Gene Ontology	GO:0015893	Drug transport	1.74E-02	ENSSSCG00000016370|ENSSSCG00000005908|ENSSSCG00000017181	*SEPTIN2|SLC52A2|CYGB*
Gene Ontology	GO:0005769	Early endosome	1.74E-02	ENSSSCG00000007469|ENSSSCG00000011853|ENSSSCG00000003577	*PTPN1|RUBCN|WASF2*
Gene Ontology	GO:0051641	Cellular localization	1.76E-02	ENSSSCG00000016569|ENSSSCG00000002965|ENSSSCG00000014061|ENSSSCG00000002287|ENSSSCG00000016420|ENSSSCG00000026293|ENSSSCG00000003342|ENSSSCG00000015524|ENSSSCG00000007469|ENSSSCG00000017251	*SMO|ACTN4|THOC3|MPP5|INSIG1|STX5|DVL1|FAM20B|PTPN1|SOX9*
Gene Ontology	GO:0051051	Negative regulation of transport	1.76E-02	ENSSSCG00000011853|ENSSSCG00000016420|ENSSSCG00000009261|ENSSSCG00000023362	*RUBCN|INSIG1|CSN2|RHBDF2*
Gene Ontology	GO:0006897	Endocytosis	1.76E-02	ENSSSCG00000007469|ENSSSCG00000011853|ENSSSCG00000003577|ENSSSCG00000002965	*PTPN1|RUBCN|WASF2|ACTN4*
Gene Ontology	GO:0038127	ERBB signaling pathway	1.76E-02	ENSSSCG00000023362|ENSSSCG00000017251	*RHBDF2|SOX9*
Gene Ontology	GO:0007272	Ensheathment of neurons	1.76E-02	ENSSSCG00000002287|ENSSSCG00000006748	*MPP5|TSPAN2*
Gene Ontology	GO:0042475	Odontogenesis of dentin-containing tooth	1.76E-02	ENSSSCG00000009266|ENSSSCG00000016569	*ODAM|SMO*
Gene Ontology	GO:0008366	Axon ensheathment	1.76E-02	ENSSSCG00000002287|ENSSSCG00000006748	*MPP5|TSPAN2*
Gene Ontology	GO:0022603	Regulation of anatomical structure morphogenesis	1.78E-02	ENSSSCG00000016569|ENSSSCG00000002965|ENSSSCG00000017251|ENSSSCG00000012520|ENSSSCG00000003342|ENSSSCG00000001660	*SMO|ACTN4|SOX9|NA|DVL1|PTK7*
Gene Ontology	GO:0045666	Positive regulation of neuron differentiation	1.84E-02	ENSSSCG00000001660|ENSSSCG00000012520|ENSSSCG00000003342	*PTK7|NA|DVL1*

Gene Ontology	GO:0048518	Positive regulation of biological process	1.85E-02	ENSSSCG00000006347|ENSSSCG00000000114|ENSSSCG00000018031|ENSSSCG00000002965|ENSSSCG00000006578|ENSSSCG00000003949|ENSSSCG00000026293|ENSSSCG00000016420|ENSSSCG00000003577|ENSSSCG00000003342|ENSSSCG00000016569|ENSSSCG00000012520|ENSSSCG00000015524|ENSSSCG00000007469|ENSSSCG00000017251|ENSSSCG00000002671|ENSSSCG00000006665|ENSSSCG00000001660|ENSSSCG00000017376|ENSSSCG00000006582|ENSSSCG00000003701	*DUSP12|PICK1|ZNF287|ACTN4|S100A4|CDC20|STX5|INSIG1|WASF2|DVL1|SMO|NA|FAM20B|PTPN1|SOX9|ATP2C2|SF3B4|PTK7|MEOX1|S100A14|SNRPD1*
Gene Ontology	GO:0004497	Monooxygenase activity	1.94E-02	ENSSSCG00000023472|ENSSSCG00000023607|ENSSSCG00000015267	*FMO3|CYP4F22|FMO2*
Gene Ontology	GO:0030048	Actin filament-based movement	1.96E-02	ENSSSCG00000003577|ENSSSCG00000002965	*WASF2|ACTN4*
Gene Ontology	GO:0001942	Hair follicle development	1.96E-02	ENSSSCG00000016569|ENSSSCG00000017251	*SMO|SOX9*
Gene Ontology	GO:0014031	Mesenchymal cell development	1.96E-02	ENSSSCG00000016569|ENSSSCG00000017251	*SMO|SOX9*
Gene Ontology	GO:0022404	Molting cycle process	1.96E-02	ENSSSCG00000016569|ENSSSCG00000017251	*SMO|SOX9*
Gene Ontology	GO:0048864	Stem cell development	1.96E-02	ENSSSCG00000016569|ENSSSCG00000017251	*SMO|SOX9*
Gene Ontology	GO:0014032	Neural crest cell development	1.96E-02	ENSSSCG00000016569|ENSSSCG00000017251	*SMO|SOX9*
Gene Ontology	GO:0022405	Hair cycle process	1.96E-02	ENSSSCG00000016569|ENSSSCG00000017251	*SMO|SOX9*
Gene Ontology	GO:0040011	Locomotion	2.03E-02	ENSSSCG00000016569|ENSSSCG00000002965|ENSSSCG00000006582|ENSSSCG00000008959|ENSSSCG00000003577|ENSSSCG00000003342|ENSSSCG00000017251|ENSSSCG00000001660	*SMO|ACTN4|S100A14|CXCL2|WASF2|DVL1|SOX9|PTK7*
Gene Ontology	GO:0007399	Nervous system development	2.03E-02	ENSSSCG00000016569|ENSSSCG00000003949|ENSSSCG00000002287|ENSSSCG00000003342|ENSSSCG00000012520|ENSSSCG00000017251|ENSSSCG00000001660|ENSSSCG00000006748|ENSSSCG00000021880	*SMO|CDC20|MPP5|DVL1|NA|SOX9|PTK7|TSPAN2|MXRA8*
Gene Ontology	GO:0034504	Protein localization to nucleus	2.05E-02	ENSSSCG00000016569|ENSSSCG00000017251|ENSSSCG00000003342	*SMO|SOX9|DVL1*
Gene Ontology	GO:0045595	Regulation of cell differentiation	2.06E-02	ENSSSCG00000016569|ENSSSCG00000002965|ENSSSCG00000003949|ENSSSCG00000016420|ENSSSCG00000003342|ENSSSCG00000012520|ENSSSCG00000017251|ENSSSCG00000001660	*SMO|ACTN4|CDC20|INSIG1|DVL1|NA|SOX9|PTK7*

Gene Ontology	GO:0033036	Macromolecule localization	2.13E-02	ENSSSCG00000016569|ENSSSCG00000002965|ENSSSCG00000023362|ENSSSCG00000014061|ENSSSCG00000002287|ENSSSCG00000016370|ENSSSCG00000026293|ENSSSCG00000003342|ENSSSCG00000015524|ENSSSCG00000007469|ENSSSCG00000017251	*SMO|ACTN4|RHBDF2|THOC3|MPP5|SEPTIN2|STX5|DVL1|FAM20B|PTPN1|SOX9*
Gene Ontology	GO:0098773	Skin epidermis development	2.16E-02	ENSSSCG00000016569|ENSSSCG00000017251	*SMO|SOX9*
Gene Ontology	GO:0006888	Endoplasmic reticulum to Golgi vesicle-mediated transport	2.16E-02	ENSSSCG00000016420|ENSSSCG00000026293	*INSIG1|STX5*
Gene Ontology	GO:0042303	Molting cycle	2.16E-02	ENSSSCG00000016569|ENSSSCG00000017251	*SMO|SOX9*
Gene Ontology	GO:0140053	Mitochondrial gene expression	2.16E-02	ENSSSCG00000001658|ENSSSCG00000016232	*MRPL2|MRPL44*
Gene Ontology	GO:0042633	Hair cycle	2.16E-02	ENSSSCG00000016569|ENSSSCG00000017251	*SMO|SOX9*
Gene Ontology	GO:0009653	Anatomical structure morphogenesis	2.16E-02	ENSSSCG00000009266|ENSSSCG00000002965|ENSSSCG00000002287|ENSSSCG00000016420|ENSSSCG00000003577|ENSSSCG00000003342|ENSSSCG00000012520|ENSSSCG00000016569|ENSSSCG00000017251|ENSSSCG00000001660|ENSSSCG00000017376	*ODAM|ACTN4|MPP5|INSIG1|WASF2|DVL1|NA|SMO|SOX9|PTK7|MEOX1*
Gene Ontology	GO:0033157	Regulation of intracellular protein transport	2.27E-02	ENSSSCG00000015524|ENSSSCG00000007469|ENSSSCG00000016569	*FAM20B|PTPN1|SMO*
Gene Ontology	GO:0048870	Cell motility	2.27E-02	ENSSSCG00000016569|ENSSSCG00000002965|ENSSSCG00000006582|ENSSSCG00000008959|ENSSSCG00000003577|ENSSSCG00000017251|ENSSSCG00000001660	*SMO|ACTN4|S100A14|CXCL2|WASF2|SOX9|PTK7*
Gene Ontology	GO:0051674	Localization of cell	2.27E-02	ENSSSCG00000016569|ENSSSCG00000002965|ENSSSCG00000006582|ENSSSCG00000008959|ENSSSCG00000003577|ENSSSCG00000017251|ENSSSCG00000001660	*SMO|ACTN4|S100A14|CXCL2|WASF2|SOX9|PTK7*
Gene Ontology	GO:0045664	Regulation of neuron differentiation	2.27E-02	ENSSSCG00000001660|ENSSSCG00000012520|ENSSSCG00000017251|ENSSSCG00000003342	*PTK7|NA|SOX9|DVL1*
Gene Ontology	GO:0048729	Tissue morphogenesis	2.30E-02	ENSSSCG00000001660|ENSSSCG00000016569|ENSSSCG00000017251|ENSSSCG00000003342|ENSSSCG00000002287	*PTK7|SMO|SOX9|DVL1|MPP5*

Gene Ontology	GO:0045833	Negative regulation of lipid metabolic process	2.37E-02	ENSSSCG00000016420|ENSSSCG00000011853	*INSIG1|RUBCN*
Gene Ontology	GO:0060349	Bone morphogenesis	2.37E-02	ENSSSCG00000016420|ENSSSCG00000017251	*INSIG1|SOX9*
Gene Ontology	GO:0072171	Mesonephric tubule morphogenesis	2.37E-02	ENSSSCG00000016569|ENSSSCG00000017251	*SMO|SOX9*
Gene Ontology	GO:0072078	Nephron tubule morphogenesis	2.37E-02	ENSSSCG00000016569|ENSSSCG00000017251	*SMO|SOX9*
Gene Ontology	GO:0060606	Tube closure	2.37E-02	ENSSSCG00000001660|ENSSSCG00000003342	*PTK7|DVL1*
Gene Ontology	GO:0001658	Branching involved in ureteric bud morphogenesis	2.37E-02	ENSSSCG00000016569|ENSSSCG00000017251	*SMO|SOX9*
Gene Ontology	GO:0090183	Regulation of kidney development	2.37E-02	ENSSSCG00000016569|ENSSSCG00000017251	*SMO|SOX9*
Gene Ontology	GO:0031532	Actin cytoskeleton reorganization	2.37E-02	ENSSSCG00000001660|ENSSSCG00000007469	*PTK7|PTPN1*
Gene Ontology	GO:0061572	Actin filament bundle organization	2.37E-02	ENSSSCG00000003577|ENSSSCG00000002965	*WASF2|ACTN4*
Gene Ontology	GO:0008013	Beta-catenin binding	2.37E-02	ENSSSCG00000017251|ENSSSCG00000003342	*SOX9|DVL1*
Gene Ontology	GO:0003279	Cardiac septum development	2.37E-02	ENSSSCG00000001660|ENSSSCG00000016569	*PTK7|SMO*
Gene Ontology	GO:0060675	Ureteric bud morphogenesis	2.37E-02	ENSSSCG00000016569|ENSSSCG00000017251	*SMO|SOX9*
Gene Ontology	GO:0001843	Neural tube closure	2.37E-02	ENSSSCG00000001660|ENSSSCG00000003342	*PTK7|DVL1*
Gene Ontology	GO:0051017	Actin filament bundle assembly	2.37E-02	ENSSSCG00000003577|ENSSSCG00000002965	*WASF2|ACTN4*
Gene Ontology	GO:0033673	Negative regulation of kinase activity	2.38E-02	ENSSSCG00000011853|ENSSSCG00000007469|ENSSSCG00000003342	*RUBCN|PTPN1|DVL1*
Gene Ontology	GO:0001664	G protein-coupled receptor binding	2.38E-02	ENSSSCG00000008959|ENSSSCG00000003342|ENSSSCG00000006582	*CXCL2|DVL1|S100A14*
Gene Ontology	GO:0048522	Positive regulation of cellular process	2.42E-02	ENSSSCG00000006347|ENSSSCG00000000114|ENSSSCG00000018031|ENSSSCG00000002965|ENSSSCG00000003949|ENSSSCG00000006578|ENSSSCG00000016420|ENSSSCG00000003577|ENSSSCG00000003342|ENSSSCG00000016569|ENSSSCG00000012520|ENSSSCG00000015524|ENSSSCG00000007469|ENSSSCG00000017251|ENSSSCG00000006665|ENSSSCG00000001660|ENSSSCG00000017376|ENSSSCG00000006582|ENSSSCG00000003701	*DUSP12|PICK1|ZNF287|ACTN4|CDC20|S100A4|INSIG1|WASF2|DVL1|SMO|NA|FAM20B|PTPN1|SOX9|SF3B4|PTK7|MEOX1|S100A14|SNRPD1*

Gene Ontology	GO:0009888	Tissue development	2.42E-02	ENSSSCG00000016569|ENSSSCG00000002287|ENSSSCG00000003342|ENSSSCG00000009266|ENSSSCG00000004657|ENSSSCG00000017251|ENSSSCG00000002671|ENSSSCG00000001660|ENSSSCG00000017376	*SMO|MPP5|DVL1|ODAM|CEP152|SOX9|ATP2C2|PTK7|MEOX1*
Gene Ontology	GO:0019901	Protein kinase binding	2.43E-02	ENSSSCG00000004657|ENSSSCG00000007469|ENSSSCG00000017251|ENSSSCG00000003342	*CEP152|PTPN1|SOX9|DVL1*
Gene Ontology	GO:0005794	Golgi apparatus	2.50E-02	ENSSSCG00000016569|ENSSSCG00000017177|ENSSSCG00000011853|ENSSSCG00000007497|ENSSSCG00000026293|ENSSSCG00000015524|ENSSSCG00000002671	*SMO|ST6GALNAC2|RUBCN|GCNT7|STX5|FAM20B|ATP2C2*
Gene Ontology	GO:0048471	Perinuclear region of cytoplasm	2.52E-02	ENSSSCG00000006578|ENSSSCG00000002965|ENSSSCG00000002671|ENSSSCG00000006582	*S100A4|ACTN4|ATP2C2|S100A14*
Gene Ontology	GO:0005654	Nucleoplasm	2.57E-02	ENSSSCG00000003949|ENSSSCG00000002287|ENSSSCG00000011853|ENSSSCG00000028973|ENSSSCG00000026293|ENSSSCG00000017251|ENSSSCG00000006582|ENSSSCG00000015524|ENSSSCG00000023078|ENSSSCG00000021991|ENSSSCG00000004657	*CDC20|MPP5|RUBCN|PHACTR4|STX5|SOX9|S100A14|FAM20B|WDR4|NDUFV3|CEP152*
Gene Ontology	GO:2000243	Positive regulation of reproductive process	2.58E-02	ENSSSCG00000017251|ENSSSCG00000003949	*SOX9|CDC20*
Gene Ontology	GO:0001046	Core promoter sequence-specific DNA binding	2.58E-02	ENSSSCG00000017376|ENSSSCG00000017251	*MEOX1|SOX9*
Gene Ontology	GO:0007224	Smoothened signaling pathway	2.58E-02	ENSSSCG00000016569|ENSSSCG00000016370	*SMO|SEPTIN2*
Gene Ontology	GO:0061333	Renal tubule morphogenesis	2.58E-02	ENSSSCG00000016569|ENSSSCG00000017251	*SMO|SOX9*
Gene Ontology	GO:0014020	Primary neural tube formation	2.58E-02	ENSSSCG00000001660|ENSSSCG00000003342	*PTK7|DVL1*
Gene Ontology	GO:0005886	Plasma membrane	2.61E-02	ENSSSCG00000000114|ENSSSCG00000008122|ENSSSCG00000023362|ENSSSCG00000006582|ENSSSCG00000012767|ENSSSCG00000002287|ENSSSCG00000016370|ENSSSCG00000003342|ENSSSCG00000007585|ENSSSCG00000016569|ENSSSCG00000007469|ENSSSCG00000023743|ENSSSCG00000002671|ENSSSCG00000012071|ENSSSCG00000001660|ENSSSCG00000006748|ENSSSCG00000005908	*PICK1|ADRA2B|RHBDF2|S100A14|HAUS7|MPP5|SEPTIN2|DVL1|ACTB|SMO|PTPN1|CLDN6|ATP2C2|NA|PTK7|TSPAN2|SLC52A2*

Gene Ontology	GO:0048513	Animal organ development	2.67E-02	ENSSSCG00000009267|ENSSSCG00000009266|ENSSSCG00000009261|ENSSSCG00000015780|ENSSSCG00000016420|ENSSSCG00000003577|ENSSSCG00000003342|ENSSSCG00000016569|ENSSSCG00000017251|ENSSSCG00000002671|ENSSSCG00000001660|ENSSSCG00000017376|ENSSSCG00000006748	*CSN3|ODAM|CSN2|STOX2|INSIG1|WASF2|DVL1|SMO|SOX9|ATP2C2|PTK7|MEOX1|TSPAN2*
Gene Ontology	GO:0006928	Movement of cell or subcellular component	2.72E-02	ENSSSCG00000016569|ENSSSCG00000002965|ENSSSCG00000006582|ENSSSCG00000008959|ENSSSCG00000003577|ENSSSCG00000003342|ENSSSCG00000017251|ENSSSCG00000001660	*SMO|ACTN4|S100A14|CXCL2|WASF2|DVL1|SOX9|PTK7*
Gene Ontology	GO:0031981	Nuclear lumen	2.73E-02	ENSSSCG00000016370|ENSSSCG00000003949|ENSSSCG00000002287|ENSSSCG00000011853|ENSSSCG00000028973|ENSSSCG00000026293|ENSSSCG00000017251|ENSSSCG00000006582|ENSSSCG00000015524|ENSSSCG00000023078|ENSSSCG00000012767|ENSSSCG00000021991|ENSSSCG00000004657|ENSSSCG00000030177	*SEPTIN2|CDC20|MPP5|RUBCN|PHACTR4|STX5|SOX9|S100A14|FAM20B|WDR4|HAUS7|NDUFV3|CEP152|EBNA1BP2*
Gene Ontology	GO:0051348	Negative regulation of transferase activity	2.74E-02	ENSSSCG00000011853|ENSSSCG00000007469|ENSSSCG00000003342	*RUBCN|PTPN1|DVL1*
Gene Ontology	GO:0043231	Intracellular membrane-bounded organelle	2.76E-02	ENSSSCG00000009266|ENSSSCG00000002965|ENSSSCG00000003949|ENSSSCG00000026293|ENSSSCG00000011853|ENSSSCG00000017251|ENSSSCG00000021991|ENSSSCG00000007497|ENSSSCG00000002671|ENSSSCG00000008973|ENSSSCG00000007715|ENSSSCG00000017177|ENSSSCG00000000522|ENSSSCG00000014061|ENSSSCG00000006665|ENSSSCG00000007496|ENSSSCG00000016420|ENSSSCG00000006578|ENSSSCG00000015524|ENSSSCG00000023078|ENSSSCG00000004657|ENSSSCG00000017376|ENSSSCG00000014003|ENSSSCG00000030177|ENSSSCG00000006582|ENSSSCG00000002287|ENSSSCG00000028973|ENSSSCG00000016569|ENSSSCG00000001658|ENSSSCG00000012767|ENSSSCG00000018031|ENSSSCG00000016370|ENSSSCG00000016232|ENSSSCG00000003701	*ODAM|ACTN4|CDC20|STX5|RUBCN|SOX9|NDUFV3|GCNT7|ATP2C2|NA|ABHD11|ST6GALNAC2|NAP1L1|THOC3|SF3B4|RTF2|INSIG1|S100A4|FAM20B|WDR4|CEP152|MEOX1|MIER2|EBNA1BP2|S100A14|MPP5|PHACTR4|SMO|MRPL2|HAUS7|ZNF287|SEPTIN2|MRPL44|SNRPD1|*
Gene Ontology	GO:0042476	Odontogenesis	2.81E-02	ENSSSCG00000009266|ENSSSCG00000016569	*ODAM|SMO*

Gene Ontology	GO:0014033	Neural crest cell differentiation	2.81E-02	ENSSSCG00000016569|ENSSSCG00000017251	*SMO|SOX9*
Gene Ontology	GO:0050807	Regulation of synapse organization	2.81E-02	ENSSSCG00000012520|ENSSSCG00000003949	*NA|CDC20*
Gene Ontology	GO:0051100	Negative regulation of binding	2.81E-02	ENSSSCG00000016569|ENSSSCG00000003342	*SMO|DVL1*
Gene Ontology	GO:0072088	Nephron epithelium morphogenesis	2.81E-02	ENSSSCG00000016569|ENSSSCG00000017251	*SMO|SOX9*
Gene Ontology	GO:0110110	Positive regulation of animal organ morphogenesis	2.81E-02	ENSSSCG00000016569|ENSSSCG00000017251	*SMO|SOX9*
Gene Ontology	GO:0032502	Developmental process	2.95E-02	ENSSSCG00000009267|ENSSSCG00000009266|ENSSSCG00000009261|ENSSSCG00000016431|ENSSSCG00000002965|ENSSSCG00000003949|ENSSSCG00000002287|ENSSSCG00000016420|ENSSSCG00000003577|ENSSSCG00000003342|ENSSSCG00000015780|ENSSSCG00000012520|ENSSSCG00000016569|ENSSSCG00000004657|ENSSSCG00000017251|ENSSSCG00000002671|ENSSSCG00000001660|ENSSSCG00000017376|ENSSSCG00000006748|ENSSSCG00000021880	*CSN3|ODAM|CSN2|GALNTL5|ACTN4|CDC20|MPP5|INSIG1|WASF2|DVL1|STOX2|NA|SMO|CEP152|SOX9|ATP2C2|PTK7|MEOX1|TSPAN2|MXRA8*
Gene Ontology	GO:0007423	Sensory organ development	2.95E-02	ENSSSCG00000001660|ENSSSCG00000016420|ENSSSCG00000017251|ENSSSCG00000003342	*PTK7|INSIG1|SOX9|DVL1*
Gene Ontology	GO:0031982	Vesicle	2.96E-02	ENSSSCG00000016569|ENSSSCG00000002965|ENSSSCG00000006578|ENSSSCG00000006582|ENSSSCG00000002287|ENSSSCG00000011853|ENSSSCG00000016370|ENSSSCG00000003577|ENSSSCG00000026293|ENSSSCG00000003342|ENSSSCG00000012520|ENSSSCG00000007469|ENSSSCG00000002671|ENSSSCG00000008973|ENSSSCG00000021880	*SMO|ACTN4|S100A4|S100A14|MPP5|RUBCN|SEPTIN2|WASF2|STX5|DVL1|NA|PTPN1|ATP2C2|NA|MXRA8*
Gene Ontology	GO:0009887	Animal organ morphogenesis	2.99E-02	ENSSSCG00000009266|ENSSSCG00000016420|ENSSSCG00000017251|ENSSSCG00000016569|ENSSSCG00000003342|ENSSSCG00000001660	*ODAM|INSIG1|SOX9|SMO|DVL1|PTK7*
Gene Ontology	GO:0016055	Wnt signaling pathway	2.99E-02	ENSSSCG00000001660|ENSSSCG00000017251|ENSSSCG00000003342	*PTK7|SOX9|DVL1*
Gene Ontology	GO:0098792	Xenophagy	3.04E-02	ENSSSCG00000003951|ENSSSCG00000003701	*C1orf210|SNRPD1*
Gene Ontology	GO:0060688	Regulation of morphogenesis of a branching structure	3.04E-02	ENSSSCG00000016569|ENSSSCG00000017251	*SMO|SOX9*
Gene Ontology	GO:0072028	Nephron morphogenesis	3.04E-02	ENSSSCG00000016569|ENSSSCG00000017251	*SMO|SOX9*

Gene Ontology	GO:0015630	Microtubule cytoskeleton	3.05E-02	ENSSSCG00000006582|ENSSSCG00000003949|ENSSSCG00000003342|ENSSSCG00000004657|ENSSSCG00000001659|ENSSSCG00000012767	*S100A14|CDC20|DVL1|CEP152|KLC4|HAUS7*
Gene Ontology	GO:0046907	Intracellular transport	3.06E-02	ENSSSCG00000016569|ENSSSCG00000002965|ENSSSCG00000014061|ENSSSCG00000016420|ENSSSCG00000026293|ENSSSCG00000015524|ENSSSCG00000007469	*SMO|ACTN4|THOC3|INSIG1|STX5|FAM20B|PTPN1*
Gene Ontology	GO:0007275	Multicellular organism development	3.09E-02	ENSSSCG00000009267|ENSSSCG00000009266|ENSSSCG00000009261|ENSSSCG00000003949|ENSSSCG00000002287|ENSSSCG00000016420|ENSSSCG00000003577|ENSSSCG00000003342|ENSSSCG00000015780|ENSSSCG00000012520|ENSSSCG00000016569|ENSSSCG00000017251|ENSSSCG00000002671|ENSSSCG00000001660|ENSSSCG00000017376|ENSSSCG00000006748|ENSSSCG00000021880	*CSN3|ODAM|CSN2|CDC20|MPP5|INSIG1|WASF2|DVL1|STOX2|NA|SMO|SOX9|ATP2C2|PTK7|MEOX1|TSPAN2|MXRA8*
Gene Ontology	GO:0071705	Nitrogen compound transport	3.12E-02	ENSSSCG00000000114|ENSSSCG00000016569|ENSSSCG00000023362|ENSSSCG00000014061|ENSSSCG00000016370|ENSSSCG00000026293|ENSSSCG00000015524|ENSSSCG00000007469|ENSSSCG00000005908	*PICK1|SMO|RHBDF2|THOC3|SEPTIN2|STX5|FAM20B|PTPN1|SLC52A2*
Gene Ontology	GO:0198738	Cell-cell signaling by wnt	3.13E-02	ENSSSCG00000001660|ENSSSCG00000017251|ENSSSCG00000003342	*PTK7|SOX9|DVL1*
Gene Ontology	GO:0070013	Intracellular organelle lumen	3.17E-02	ENSSSCG00000023078|ENSSSCG00000003949|ENSSSCG00000002287|ENSSSCG00000011853|ENSSSCG00000028973|ENSSSCG00000026293|ENSSSCG00000017251|ENSSSCG00000016370|ENSSSCG00000006582|ENSSSCG00000015524|ENSSSCG00000001658|ENSSSCG00000012767|ENSSSCG00000021991|ENSSSCG00000004657|ENSSSCG00000030177	*WDR4|CDC20|MPP5|RUBCN|PHACTR4|STX5|SOX9|SEPTIN2|S100A14|FAM20B|MRPL2|HAUS7|NDUFV3|CEP152|EBNA1BP2*
Gene Ontology	GO:0031974	Membrane-enclosed lumen	3.17E-02	ENSSSCG00000023078|ENSSSCG00000003949|ENSSSCG00000002287|ENSSSCG00000011853|ENSSSCG00000028973|ENSSSCG00000026293|ENSSSCG00000017251|ENSSSCG00000016370|ENSSSCG00000006582|ENSSSCG00000015524|ENSSSCG00000001658|ENSSSCG00000012767|ENSSSCG00000021991|ENSSSCG00000004657|ENSSSCG00000030177	*WDR4|CDC20|MPP5|RUBCN|PHACTR4|STX5|SOX9|SEPTIN2|S100A14|FAM20B|MRPL2|HAUS7|NDUFV3|CEP152|EBNA1BP2*

Gene Ontology	GO:0043233	Organelle lumen	3.17E-02	ENSSSCG00000023078|ENSSSCG00000003949|ENSSSCG00000002287|ENSSSCG00000011853|ENSSSCG00000028973|ENSSSCG00000026293|ENSSSCG00000017251|ENSSSCG00000016370|ENSSSCG00000006582|ENSSSCG00000015524|ENSSSCG00000001658|ENSSSCG00000012767|ENSSSCG00000021991|ENSSSCG00000004657|ENSSSCG00000030177	*WDR4|CDC20|MPP5|RUBCN|PHACTR4|STX5|SOX9|SEPTIN2|S100A14|FAM20B|MRPL2|HAUS7|NDUFV3|CEP152|EBNA1BP2*
Gene Ontology	GO:0030182	Neuron differentiation	3.18E-02	ENSSSCG00000016569|ENSSSCG00000017251|ENSSSCG00000012520|ENSSSCG00000003342|ENSSSCG00000001660|ENSSSCG00000006748	*SMO|SOX9|NA|DVL1|PTK7|TSPAN2*
Gene Ontology	GO:0016020	Membrane	3.20E-02	ENSSSCG00000000114|ENSSSCG00000007497|ENSSSCG00000023636|ENSSSCG00000003755|ENSSSCG00000007469|ENSSSCG00000002671|ENSSSCG00000001660|ENSSSCG00000015405|ENSSSCG00000017177|ENSSSCG00000000522|ENSSSCG00000016420|ENSSSCG00000005707|ENSSSCG00000003342|ENSSSCG00000023472|ENSSSCG00000023607|ENSSSCG00000023743|ENSSSCG00000006748|ENSSSCG00000006582|ENSSSCG00000002287|ENSSSCG00000003951|ENSSSCG00000012767|ENSSSCG00000005908|ENSSSCG00000021880|ENSSSCG00000016569|ENSSSCG00000016431|ENSSSCG00000023362|ENSSSCG00000015267|ENSSSCG00000012071|ENSSSCG00000016370|ENSSSCG00000026293|ENSSSCG00000007585|ENSSSCG00000021991|ENSSSCG00000008122	*PICK1|GCNT7|TMEM222|MCOLN2|PTPN1|ATP2C2|PTK7|CD36|ST6GALNAC2|NAP1L1|INSIG1|FIBCD1|DVL1|FMO3|CYP4F22|CLDN6|TSPAN2|S100A14|MPP5|C1orf210|HAUS7|SLC52A2|MXRA8|SMO|GALNTL5|RHBDF2|FMO2|NA|SEPTIN2|STX5|ACTB|NDUFV3|ADRA2B*
Gene Ontology	GO:0010975	Regulation of neuron projection development	3.26E-02	ENSSSCG00000001660|ENSSSCG00000012520|ENSSSCG00000003342	*PTK7|NA|DVL1*
Gene Ontology	GO:0050803	Regulation of synapse structure or activity	3.28E-02	ENSSSCG00000012520|ENSSSCG00000003949	*NA|CDC20*
Gene Ontology	GO:0016358	Dendrite development	3.28E-02	ENSSSCG00000012520|ENSSSCG00000003342	*NA|DVL1*
Gene Ontology	GO:0050660	Flavin adenine dinucleotide binding	3.28E-02	ENSSSCG00000023472|ENSSSCG00000015267	*FMO3|FMO2*
Gene Ontology	GO:0061053	Somite development	3.28E-02	ENSSSCG00000017376|ENSSSCG00000016569	*MEOX1|SMO*
Gene Ontology	GO:0071229	Cellular response to acid chemical	3.28E-02	ENSSSCG00000001660|ENSSSCG00000017251	*PTK7|SOX9*
Gene Ontology	GO:0001708	Cell fate specification	3.28E-02	ENSSSCG00000016569|ENSSSCG00000017251	*SMO|SOX9*

Gene Ontology	GO:0031090	Organelle membrane	3.30E-02	ENSSSCG00000016569|ENSSSCG00000017177|ENSSSCG00000023362|ENSSSCG00000016420|ENSSSCG00000026293|ENSSSCG00000016370|ENSSSCG00000023472|ENSSSCG00000007469|ENSSSCG00000007497|ENSSSCG00000002671|ENSSSCG00000021991	*SMO|ST6GALNAC2|RHBDF2|INSIG1|STX5|SEPTIN2|FMO3|PTPN1|GCNT7|ATP2C2|NDUFV3*
Gene Ontology	GO:0019900	Kinase binding	3.34E-02	ENSSSCG00000004657|ENSSSCG00000007469|ENSSSCG00000017251|ENSSSCG00000003342	*CEP152|PTPN1|SOX9|DVL1*
Gene Ontology	GO:0016705	Oxidoreductase activity, acting on paired donors, with incorporation or reduction of molecular oxygen	3.40E-02	ENSSSCG00000023472|ENSSSCG00000023607|ENSSSCG00000015267	*FMO3|CYP4F22|FMO2*
Gene Ontology	GO:0030154	Cell differentiation	3.50E-02	ENSSSCG00000016569|ENSSSCG00000016431|ENSSSCG00000002965|ENSSSCG00000003949|ENSSSCG00000002287|ENSSSCG00000016420|ENSSSCG00000003577|ENSSSCG00000003342|ENSSSCG00000012520|ENSSSCG00000004657|ENSSSCG00000017251|ENSSSCG00000001660|ENSSSCG00000006748|ENSSSCG00000021880	*SMO|GALNTL5|ACTN4|CDC20|MPP5|INSIG1|WASF2|DVL1|NA|CEP152|SOX9|PTK7|TSPAN2|MXRA8*
Gene Ontology	GO:0050691	Regulation of defense response to virus by host	3.53E-02	ENSSSCG00000003951|ENSSSCG00000003701	*C1orf210|SNRPD1*
Gene Ontology	GO:0035265	Organ growth	3.53E-02	ENSSSCG00000016569|ENSSSCG00000017251	*SMO|SOX9*
Gene Ontology	GO:0001841	Neural tube formation	3.53E-02	ENSSSCG00000001660|ENSSSCG00000003342	*PTK7|DVL1*
Gene Ontology	GO:0002230	Positive regulation of defense response to virus by host	3.53E-02	ENSSSCG00000003951|ENSSSCG00000003701	*C1orf210|SNRPD1*
Gene Ontology	GO:0051093	Negative regulation of developmental process	3.59E-02	ENSSSCG00000016420|ENSSSCG00000009261|ENSSSCG00000002965|ENSSSCG00000017251|ENSSSCG00000016569	*INSIG1|CSN2|ACTN4|SOX9|SMO*
Gene Ontology	GO:0051704	Multi-organism process	3.78E-02	ENSSSCG00000015780|ENSSSCG00000016431|ENSSSCG00000003949|ENSSSCG00000014061|ENSSSCG00000006582|ENSSSCG00000003951|ENSSSCG00000003342|ENSSSCG00000017251|ENSSSCG00000005908|ENSSSCG00000003701	*STOX2|GALNTL5|CDC20|THOC3|S100A14|C1orf210|DVL1|SOX9|SLC52A2|SNRPD1*
Gene Ontology	GO:0072164	Mesonephric tubule development	3.78E-02	ENSSSCG00000016569|ENSSSCG00000017251	*SMO|SOX9*
Gene Ontology	GO:0072163	Mesonephric epithelium development	3.78E-02	ENSSSCG00000016569|ENSSSCG00000017251	*SMO|SOX9*
Gene Ontology	GO:0072080	Nephron tubule development	3.78E-02	ENSSSCG00000016569|ENSSSCG00000017251	*SMO|SOX9*

Gene Ontology	GO:0001657	Ureteric bud development	3.78E-02	ENSSSCG00000016569|ENSSSCG00000017251	*SMO|SOX9*
Gene Ontology	GO:0001656	Metanephros development	3.78E-02	ENSSSCG00000016569|ENSSSCG00000017251	*SMO|SOX9*
Gene Ontology	GO:0061326	Renal tubule development	3.78E-02	ENSSSCG00000016569|ENSSSCG00000017251	*SMO|SOX9*
Gene Ontology	GO:0048646	Anatomical structure formation involved in morphogenesis	3.86E-02	ENSSSCG00000016569|ENSSSCG00000003577|ENSSSCG00000017251|ENSSSCG00000003342|ENSSSCG00000001660|ENSSSCG00000017376	*SMO|WASF2|SOX9|DVL1|PTK7|MEOX1*
Gene Ontology	GO:0061024	Membrane organization	3.96E-02	ENSSSCG00000002287|ENSSSCG00000016420|ENSSSCG00000026293|ENSSSCG00000003342	*MPP5|INSIG1|STX5|DVL1*
Gene Ontology	GO:0051129	Negative regulation of cellular component organization	3.96E-02	ENSSSCG00000011853|ENSSSCG00000016420|ENSSSCG00000003577|ENSSSCG00000002965	*RUBCN|INSIG1|WASF2|ACTN4*
Gene Ontology	GO:0007015	Actin filament organization	3.98E-02	ENSSSCG00000000114|ENSSSCG00000003577|ENSSSCG00000002965	*PICK1|WASF2|ACTN4*
Gene Ontology	GO:0006334	Nucleosome assembly	4.05E-02	ENSSSCG00000017251|ENSSSCG00000000522	*SOX9|NAP1L1*
Gene Ontology	GO:0001823	Mesonephros development	4.05E-02	ENSSSCG00000016569|ENSSSCG00000017251	*SMO|SOX9*
Gene Ontology	GO:0045995	Regulation of embryonic development	4.05E-02	ENSSSCG00000001660|ENSSSCG00000003342	*PTK7|DVL1*
Gene Ontology	GO:0001667	Ameboidal-type cell migration	4.13E-02	ENSSSCG00000016569|ENSSSCG00000003577|ENSSSCG00000017251	*SMO|WASF2|SOX9*
Gene Ontology	GO:0048589	Developmental growth	4.30E-02	ENSSSCG00000001660|ENSSSCG00000016569|ENSSSCG00000017251|ENSSSCG00000003342	*PTK7|SMO|SOX9|DVL1*
Gene Ontology	GO:0034329	Cell junction assembly	4.31E-02	ENSSSCG00000002287|ENSSSCG00000002965	*MPP5|ACTN4*
Gene Ontology	GO:0060828	Regulation of canonical Wnt signaling pathway	4.31E-02	ENSSSCG00000017251|ENSSSCG00000003342	*SOX9|DVL1*
Gene Ontology	GO:0051701	Interaction with host	4.31E-02	ENSSSCG00000014061|ENSSSCG00000005908	*THOC3|SLC52A2*
Gene Ontology	GO:0005875	Microtubule associated complex	4.31E-02	ENSSSCG00000012767|ENSSSCG00000001659	*HAUS7|KLC4*
Gene Ontology	GO:0016709	Oxidoreductase activity, acting on paired donors, with incorporation or reduction of molecular oxygen, NAD(P)H as one donor, and incorporation of one atom of oxygen	4.31E-02	ENSSSCG00000023472|ENSSSCG00000015267	*FMO3|FMO2*

Gene Ontology	GO:0120025	Plasma membrane bounded cell projection	4.40E-02	ENSSSCG00000016569|ENSSSCG00000002965|ENSSSCG00000016370|ENSSSCG00000003577|ENSSSCG00000017181|ENSSSCG00000003342	*SMO|ACTN4|SEPTIN2|WASF2|CYGB|DVL1*
Gene Ontology	GO:0051338	Regulation of transferase activity	4.48E-02	ENSSSCG00000006347|ENSSSCG00000011853|ENSSSCG00000007469|ENSSSCG00000003342|ENSSSCG00000003949	*DUSP12|RUBCN|PTPN1|DVL1|CDC20*
Gene Ontology	GO:0048699	Generation of neurons	4.56E-02	ENSSSCG00000016569|ENSSSCG00000017251|ENSSSCG00000012520|ENSSSCG00000003342|ENSSSCG00000001660|ENSSSCG00000006748	*SMO|SOX9|NA|DVL1|PTK7|TSPAN2*
Gene Ontology	GO:0003205	Cardiac chamber development	4.59E-02	ENSSSCG00000001660|ENSSSCG00000016569	*PTK7|SMO*
Gene Ontology	GO:0072009	Nephron epithelium development	4.59E-02	ENSSSCG00000016569|ENSSSCG00000017251	*SMO|SOX9*
Gene Ontology	GO:0060993	Kidney morphogenesis	4.59E-02	ENSSSCG00000016569|ENSSSCG00000017251	*SMO|SOX9*
Gene Ontology	GO:0030856	Regulation of epithelial cell differentiation	4.59E-02	ENSSSCG00000016569|ENSSSCG00000017251	*SMO|SOX9*
Gene Ontology	GO:0042995	Cell projection	4.72E-02	ENSSSCG00000016569|ENSSSCG00000002965|ENSSSCG00000016370|ENSSSCG00000003577|ENSSSCG00000017181|ENSSSCG00000003342	*SMO|ACTN4|SEPTIN2|WASF2|CYGB|DVL1*
Gene Ontology	GO:0051254	Positive regulation of RNA metabolic process	4.75E-02	ENSSSCG00000018031|ENSSSCG00000002965|ENSSSCG00000006665|ENSSSCG00000016420|ENSSSCG00000003342|ENSSSCG00000016569|ENSSSCG00000017251|ENSSSCG00000017376	*ZNF287|ACTN4|SF3B4|INSIG1|DVL1|SMO|SOX9|MEOX1*
Gene Ontology	GO:0050878	Regulation of body fluid levels	4.77E-02	ENSSSCG00000009267|ENSSSCG00000008122|ENSSSCG00000009261	*CSN3|ADRA2B|CSN2*

<table>
<tr><td>Gene Ontology</td><td>GO:0009987</td><td>Cellular process</td><td>4.82E-02</td><td>ENSSSCG00000000114|ENSSSCG00000016370|ENSSSCG00000002965|ENSSSCG00000003949|ENSSSCG00000026293|ENSSSCG00000011853|ENSSSCG00000017251|ENSSSCG00000012520|ENSSSCG00000007469|ENSSSCG00000007497|ENSSSCG00000002671|ENSSSCG00000001660|ENSSSCG00000006347|ENSSSCG00000015405|ENSSSCG00000006578|ENSSSCG00000017177|ENSSSCG00000000522|ENSSSCG00000007494|ENSSSCG00000006665|ENSSSCG00000007496|ENSSSCG00000016420|ENSSSCG00000003577|ENSSSCG00000003342|ENSSSCG00000012842|ENSSSCG00000015524|ENSSSCG00000023078|ENSSSCG00000023743|ENSSSCG00000004657|ENSSSCG00000017376|ENSSSCG00000006748|ENSSSCG00000030177|ENSSSCG00000015717|ENSSSCG00000006582|ENSSSCG00000002287|ENSSSCG00000028973|ENSSSCG00000003951|ENSSSCG00000017904|ENSSSCG00000016569|ENSSSCG00000001658|ENSSSCG00000012767|ENSSSCG00000020705|ENSSSCG00000021880|ENSSSCG00000018031|ENSSSCG00000016431|ENSSSCG00000023362|ENSSSCG00000015267|ENSSSCG00000008959|ENSSSCG00000015270|ENSSSCG00000016232|ENSSSCG00000008122|ENSSSCG00000003701</td><td>*PICK1|SEPTIN2|ACTN4|CDC20|STX5|RUBCN|SOX9|NA|PTPN1|GCNT7|ATP2C2|PTK7|DUSP12|CD36|S100A4|ST6GALNAC2|NAP1L1|CSTF1|SF3B4|RTF2|INSIG1|WASF2|DVL1|RPLP2|FAM20B|WDR4|CLDN6|CEP152|MEOX1|TSPAN2|EBNA1BP2|C1QL2|S100A14|MPP5|PHACTR4|C1orf210|SMO|MRPL2|HAUS7|MAP3K8|MXRA8|ZNF287|GALNTL5|RHBDF2|FMO2|CXCL2|FMOD|MRPL44|ADRA2B|SNRPD1*</td></tr>
<tr><td>Gene Ontology</td><td>GO:0009893</td><td>Positive regulation of metabolic process</td><td>4.84E-02</td><td>ENSSSCG00000006347|ENSSSCG00000016569|ENSSSCG00000002965|ENSSSCG00000003949|ENSSSCG00000006665|ENSSSCG00000016420|ENSSSCG00000026293|ENSSSCG00000003342|ENSSSCG00000018031|ENSSSCG00000007469|ENSSSCG00000017251|ENSSSCG00000017376|ENSSSCG00000003701</td><td>*DUSP12|SMO|ACTN4|CDC20|SF3B4|INSIG1|STX5|DVL1|ZNF287|PTPN1|SOX9|MEOX1|SNRPD1*</td></tr>
<tr><td>Gene Ontology</td><td>GO:0005615</td><td>Extracellular space</td><td>4.84E-02</td><td>ENSSSCG00000009267|ENSSSCG00000016569|ENSSSCG00000009261|ENSSSCG00000002965|ENSSSCG00000006578|ENSSSCG00000015270|ENSSSCG00000008959|ENSSSCG00000009262|ENSSSCG00000003577|ENSSSCG00000016370|ENSSSCG00000012520|ENSSSCG00000002287|ENSSSCG00000006582|ENSSSCG00000008973|ENSSSCG00000021880</td><td>*CSN3|SMO|CSN2|ACTN4|S100A4|FMOD|CXCL2|CSN1S1|WASF2|SEPTIN2|NA|MPP5|S100A14|NA|MXRA8*</td></tr>
</table>

<table>
<tr><td>Gene Ontology</td><td>GO:0034728</td><td>Nucleosome organization</td><td>4.87E-02</td><td>ENSSSCG00000017251|ENSSSCG00000000522</td><td>SOX9|NAP1L1</td></tr>
<tr><td>Gene Ontology</td><td>GO:0007163</td><td>Establishment or maintenance of cell polarity</td><td>4.87E-02</td><td>ENSSSCG00000001660|ENSSSCG00000002287</td><td>PTK7|MPP5</td></tr>
<tr><td>Gene Ontology</td><td>GO:0030855</td><td>Epithelial cell differentiation</td><td>4.89E-02</td><td>ENSSSCG00000001660|ENSSSCG00000016569|ENSSSCG00000004657|ENSSSCG00000017251</td><td>PTK7|SMO|CEP152|SOX9</td></tr>
<tr><td>Gene Ontology</td><td>GO:0005622</td><td>Intracellular</td><td>4.92E-02</td><td>ENSSSCG00000000114|ENSSSCG00000009266|ENSSSCG00000002965|ENSSSCG00000003949|ENSSSCG00000026293|ENSSSCG00000011853|ENSSSCG00000017251|ENSSSCG00000021991|ENSSSCG00000007497|ENSSSCG00000002671|ENSSSCG00000008973|ENSSSCG00000007715|ENSSSCG00000006578|ENSSSCG00000017177|ENSSSCG00000000522|ENSSSCG00000014061|ENSSSCG00000006665|ENSSSCG00000007496|ENSSSCG00000016420|ENSSSCG00000003577|ENSSSCG00000003342|ENSSSCG00000012842|ENSSSCG00000015524|ENSSSCG00000023078|ENSSSCG00000004657|ENSSSCG00000017376|ENSSSCG00000014003|ENSSSCG00000030177|ENSSSCG00000006582|ENSSSCG00000002287|ENSSSCG00000028973|ENSSSCG00000017904|ENSSSCG00000016569|ENSSSCG00000001658|ENSSSCG00000001659|ENSSSCG00000012767|ENSSSCG00000020705|ENSSSCG00000018031|ENSSSCG00000016370|ENSSSCG00000016232|ENSSSCG00000007585|ENSSSCG00000002959|ENSSSCG00000007469|ENSSSCG00000003701</td><td>PICK1|ODAM|ACTN4|CDC20|STX5|RUBCN|SOX9|NDUFV3|GCNT7|ATP2C2|NA|ABHD11|S100A4|ST6GALNAC2|NAP1L1|THOC3|SF3B4|RTF2|INSIG1|WASF2|DVL1|RPLP2|FAM20B|WDR4|CEP152|MEOX1|MIER2|EBNA1BP2|S100A14|MPP5|PHACTR4|ENO3|SMO|MRPL2|KLC4|HAUS7|ZNF287|SEPTIN2|MRPL44|ACTB|FAM98C|PTPN1|SNRPD1</td></tr>
</table>

Note: NA indicates novel gene.

Table S6 Significant GO and KEGG pathways of lncRNAs in cluster 4, 7, 9, 10 and 12

Database	ID	Term	*P* -value	Genes	Gene symbol
KEGG PATHWAY	Oocyte meiosis	ssc04114	3.91E-03	ENSSSCG00000016726|ENSSSCG00000003949|ENSSSCG00000029264	*ADCY1|CDC20|PKMYT1*
KEGG PATHWAY	Tight junction	ssc04530	1.14E-02	ENSSSCG00000005191|ENSSSCG00000023743|ENSSSCG00000007585	*MPDZ|CLDN6|ACTB*
KEGG PATHWAY	Adherens junction	ssc04520	1.63E-02	ENSSSCG00000007469|ENSSSCG00000007585	*PTPN1|ACTB*
KEGG PATHWAY	Rap1 signaling pathway	ssc04015	1.90E-02	ENSSSCG00000014007|ENSSSCG00000016726|ENSSSCG00000007585	*FLT4|ADCY1|ACTB*
KEGG PATHWAY	Dilated cardiomyopathy (DCM)	ssc05414	2.29E-02	ENSSSCG00000016726|ENSSSCG00000007585	*ADCY1|ACTB*
KEGG PATHWAY	Progesterone-mediated oocyte maturation	ssc04914	2.84E-02	ENSSSCG00000016726|ENSSSCG00000029264	*ADCY1|PKMYT1*
KEGG PATHWAY	Selenocompound metabolism	ssc00450	3.78E-02	ENSSSCG00000010143	*MTR*
KEGG PATHWAY	Leukocyte transendothelial migration	ssc04670	3.79E-02	ENSSSCG00000023743|ENSSSCG00000007585	*CLDN6|ACTB*
KEGG PATHWAY	Platelet activation	ssc04611	4.54E-02	ENSSSCG00000016726|ENSSSCG00000007585	*ADCY1|ACTB*
KEGG PATHWAY	Cell cycle	ssc04110	4.77E-02	ENSSSCG00000003949|ENSSSCG00000029264	*CDC20|PKMYT1*
Gene Ontology	U2-type precatalytic spliceosome	GO:0071005	1.30E-04	ENSSSCG00000010999|ENSSSCG00000006665|ENSSSCG00000023304	*SMU1|SF3B4|SRRM2*
Gene Ontology	Schmidt-Lanterman incisure	GO:0043220	2.09E-04	ENSSSCG00000005191|ENSSSCG00000003547	*MPDZ|NCMAP*
Gene Ontology	THO complex part of transcription export complex	GO:0000445	2.78E-04	ENSSSCG00000023315|ENSSSCG00000014061	*THOC6|THOC3*
Gene Ontology	apicolateral plasma membrane	GO:0016327	5.43E-04	ENSSSCG00000005191|ENSSSCG00000023743	*MPDZ|CLDN6*
Gene Ontology	mRNA splicing, via spliceosome	GO:0000398	2.37E-03	ENSSSCG00000010999|ENSSSCG00000006665|ENSSSCG00000023304	*SMU1|SF3B4|SRRM2*
Gene Ontology	positive regulation of protein tyrosine kinase activity	GO:0061098	2.44E-03	ENSSSCG00000009565|ENSSSCG00000007469	*GAS6|PTPN1*
Gene Ontology	mRNA export from nucleus	GO:0006406	3.87E-03	ENSSSCG00000023315|ENSSSCG00000014061	*THOC6|THOC3*
Gene Ontology	axonogenesis	GO:0007409	4.70E-03	ENSSSCG00000016726|ENSSSCG00000007585	*ADCY1|ACTB*
Gene Ontology	receptor tyrosine kinase binding	GO:0030971	5.29E-03	ENSSSCG00000009565|ENSSSCG00000007469	*GAS6|PTPN1*
Gene Ontology	protein N-terminus binding	GO:0047485	1.12E-02	ENSSSCG00000023304|ENSSSCG00000005383	*SRRM2|ALG2*
Gene Ontology	bicellular tight junction	GO:0005923	1.34E-02	ENSSSCG00000005191|ENSSSCG00000023743	*MPDZ|CLDN6*
Gene Ontology	cell division	GO:0051301	1.34E-02	ENSSSCG00000003949|ENSSSCG00000009653	*CDC20|CDCA2*
Gene Ontology	calcium ion transmembrane transport	GO:0070588	1.84E-02	ENSSSCG00000009565|ENSSSCG00000002671	*GAS6|ATP2C2*

Gene Ontology	structural constituent of myelin sheath	GO:0019911	1.91E-02	ENSSSCG00000003547	*NCMAP*
Gene Ontology	cellular response to interferon-alpha	GO:0035457	1.91E-02	ENSSSCG00000009565	*GAS6*
Gene Ontology	positive regulation of natural killer cell differentiation	GO:0032825	1.91E-02	ENSSSCG00000009565	*GAS6*
Gene Ontology	oligosaccharide-lipid intermediate biosynthetic process	GO:0006490	1.91E-02	ENSSSCG00000005383	*ALG2*
Gene Ontology	negative regulation of fibroblast apoptotic process	GO:2000270	1.91E-02	ENSSSCG00000009565	*GAS6*
Gene Ontology	negative regulation of vascular endothelial growth factor receptor signaling pathway	GO:0030948	1.91E-02	ENSSSCG00000007469	*PTPN1*
Gene Ontology	postsynaptic actin cytoskeleton organization	GO:0098974	1.91E-02	ENSSSCG00000007585	*ACTB*
Gene Ontology	peptidyl-tyrosine dephosphorylation involved in inactivation of protein kinase activity	GO:1990264	1.91E-02	ENSSSCG00000007469	*PTPN1*
Gene Ontology	lymphangiogenesis	GO:0001946	1.91E-02	ENSSSCG00000014007	*FLT4*
Gene Ontology	cobalamin metabolic process	GO:0009235	1.91E-02	ENSSSCG00000010143	*MTR*
Gene Ontology	thioesterase binding	GO:0031996	1.91E-02	ENSSSCG00000012767	*HAUS7*
Gene Ontology	SREBP signaling pathway	GO:0032933	1.91E-02	ENSSSCG00000016420	*INSIG1*
Gene Ontology	lysosomal membrane	GO:0005765	2.12E-02	ENSSSCG00000003755|ENSSSCG00000002672	*MCOLN2|MEAK7*
Gene Ontology	positive regulation of meiotic cell cycle process involved in oocyte maturation	GO:1904146	2.22E-02	ENSSSCG00000003949	*CDC20*
Gene Ontology	lactate transmembrane transport	GO:0035873	2.22E-02	ENSSSCG00000024018	*SLC16A3*
Gene Ontology	regulation of intracellular protein transport	GO:0033157	2.22E-02	ENSSSCG00000007469	*PTPN1*
Gene Ontology	lactate transmembrane transporter activity	GO:0015129	2.22E-02	ENSSSCG00000024018	*SLC16A3*
Gene Ontology	epinephrine binding	GO:0051379	2.22E-02	ENSSSCG00000008122	*ADRA2B*
Gene Ontology	positive regulation of establishment of protein localization to telomere	GO:1904851	2.22E-02	ENSSSCG00000006487	*CCT3*
Gene Ontology	regulation of myelination	GO:0031641	2.22E-02	ENSSSCG00000003547	*NCMAP*
Gene Ontology	regulation of vasoconstriction	GO:0019229	2.22E-02	ENSSSCG00000008122	*ADRA2B*
Gene Ontology	response to amino acid	GO:0043200	2.22E-02	ENSSSCG00000002672	*MEAK7*

Gene Ontology	HAUS complex	GO:0070652	2.22E-02	ENSSSCG00000012767	*HAUS7*
Gene Ontology	zona pellucida receptor complex	GO:0002199	2.22E-02	ENSSSCG00000006487	*CCT3*
Gene Ontology	negative regulation of biomineral tissue development	GO:0070168	2.22E-02	ENSSSCG00000009565	*GAS6*
Gene Ontology	response to progesterone	GO:0032570	2.22E-02	ENSSSCG00000009262	*CSN1S1*
Gene Ontology	positive regulation of CREB transcription factor activity	GO:0032793	2.54E-02	ENSSSCG00000016726	*ADCY1*
Gene Ontology	C2H2 zinc finger domain binding	GO:0070742	2.54E-02	ENSSSCG00000023304	*SRRM2*
Gene Ontology	receptor ligand activity	GO:0048018	2.54E-02	ENSSSCG00000009565	*GAS6*
Gene Ontology	chaperonin-containing T-complex	GO:0005832	2.54E-02	ENSSSCG00000006487	*CCT3*
Gene Ontology	apoptotic cell clearance	GO:0043277	2.54E-02	ENSSSCG00000009565	*GAS6*
Gene Ontology	integral component of plasma membrane	GO:0005887	2.84E-02	ENSSSCG00000014007|ENSSSCG00000016726|ENSSSCG00000024018|ENSSSCG00000008122|ENSSSCG00000003547	*FLT4|ADCY1|SLC16A3|ADRA2B|NCMAP*
Gene Ontology	cobalamin binding	GO:0031419	2.85E-02	ENSSSCG00000010143	*MTR*
Gene Ontology	cAMP biosynthetic process	GO:0006171	2.85E-02	ENSSSCG00000016726	*ADCY1*
Gene Ontology	manganese ion transport	GO:0006828	2.85E-02	ENSSSCG00000002671	*ATP2C2*
Gene Ontology	dendritic cell differentiation	GO:0097028	2.85E-02	ENSSSCG00000009565	*GAS6*
Gene Ontology	adenylate cyclase activity	GO:0004016	2.85E-02	ENSSSCG00000016726	*ADCY1*
Gene Ontology	virus receptor activity	GO:0001618	2.85E-02	ENSSSCG00000023743	*CLDN6*
Gene Ontology	paranode region of axon	GO:0033270	2.85E-02	ENSSSCG00000003547	*NCMAP*
Gene Ontology	protein C-terminus binding	GO:0008022	3.10E-02	ENSSSCG00000005191|ENSSSCG00000003949	*MPDZ|CDC20*
Gene Ontology	mannosylation	GO:0097502	3.16E-02	ENSSSCG00000005383	*ALG2*
Gene Ontology	sterol biosynthetic process	GO:0016126	3.16E-02	ENSSSCG00000016420	*INSIG1*
Gene Ontology	negative regulation of interferon-gamma production	GO:0032689	3.16E-02	ENSSSCG00000009565	*GAS6*
Gene Ontology	vascular endothelial growth factor signaling pathway	GO:0038084	3.16E-02	ENSSSCG00000014007	*FLT4*
Gene Ontology	respiratory system process	GO:0003016	3.16E-02	ENSSSCG00000014007	*FLT4*
Gene Ontology	protein kinase C signaling	GO:0070528	3.16E-02	ENSSSCG00000030518	*PRSS33*

Gene Ontology	microtubule minus-end binding	GO:0051011	3.16E-02	ENSSSCG00000012767	*HAUS7*
Gene Ontology	negative regulation of tumor necrosis factor-mediated signaling pathway	GO:0010804	3.16E-02	ENSSSCG00000009565	*GAS6*
Gene Ontology	positive regulation of protein kinase C signaling	GO:0090037	3.16E-02	ENSSSCG00000014007	*FLT4*
Gene Ontology	vascular endothelial growth factor receptor signaling pathway	GO:0048010	3.16E-02	ENSSSCG00000014007	*FLT4*
Gene Ontology	positive regulation of protein export from nucleus	GO:0046827	3.16E-02	ENSSSCG00000009565	*GAS6*
Gene Ontology	activation of protein kinase B activity	GO:0032148	3.47E-02	ENSSSCG00000008122	*ADRA2B*
Gene Ontology	mitotic sister chromatid cohesion	GO:0007064	3.47E-02	ENSSSCG00000003949	*CDC20*
Gene Ontology	cell motility	GO:0048870	3.47E-02	ENSSSCG00000007585	*ACTB*
Gene Ontology	pore complex assembly	GO:0046931	3.47E-02	ENSSSCG00000006487	*CCT3*
Gene Ontology	dolichol-linked oligosaccharide biosynthetic process	GO:0006488	3.47E-02	ENSSSCG00000005383	*ALG2*
Gene Ontology	calcium transmembrane transporter activity, phosphorylative mechanism	GO:0005388	3.47E-02	ENSSSCG00000002671	*ATP2C2*
Gene Ontology	precatalytic spliceosome	GO:0071011	3.78E-02	ENSSSCG00000010999	*SMU1*
Gene Ontology	negative regulation of endoplasmic reticulum stress-induced intrinsic apoptotic signaling pathway	GO:1902236	3.78E-02	ENSSSCG00000007469	*PTPN1*
Gene Ontology	cell-substrate adhesion	GO:0031589	3.78E-02	ENSSSCG00000009565	*GAS6*
Gene Ontology	pericentriolar material	GO:0000242	3.78E-02	ENSSSCG00000004657	*CEP152*
Gene Ontology	positive regulation of protein dephosphorylation	GO:0035307	3.78E-02	ENSSSCG00000009653	*CDCA2*
Gene Ontology	middle ear morphogenesis	GO:0042474	3.78E-02	ENSSSCG00000016420	*INSIG1*
Gene Ontology	long-term memory	GO:0007616	3.78E-02	ENSSSCG00000016726	*ADCY1*
Gene Ontology	Notch binding	GO:0005112	3.78E-02	ENSSSCG00000016381	*SNED1*
Gene Ontology	nucleolus	GO:0005730	3.94E-02	ENSSSCG00000002672|ENSSSCG00000010994|ENSSSCG00000030177|ENSSSCG00000029264	*MEAK7|NFX1|EBNA1BP2|PKMYT1*
Gene Ontology	nuclear periphery	GO:0034399	4.09E-02	ENSSSCG00000030177	*EBNA1BP2*

Gene Ontology	response to estradiol	GO:0032355	4.09E-02	ENSSSCG00000009262	*CSN1S1*
Gene Ontology	positive regulation of vascular endothelial growth factor production	GO:0010575	4.09E-02	ENSSSCG00000014007	*FLT4*
Gene Ontology	histone deacetylation	GO:0016575	4.09E-02	ENSSSCG00000014003	*MIER2*
Gene Ontology	positive regulation of telomere maintenance via telomerase	GO:0032212	4.09E-02	ENSSSCG00000006487	*CCT3*
Gene Ontology	triglyceride metabolic process	GO:0006641	4.40E-02	ENSSSCG00000016420	*INSIG1*
Gene Ontology	cysteine-type endopeptidase inhibitor activity involved in apoptotic process	GO:0043027	4.40E-02	ENSSSCG00000009565	*GAS6*
Gene Ontology	centriole replication	GO:0007099	4.40E-02	ENSSSCG00000004657	*CEP152*
Gene Ontology	anaphase-promoting complex-dependent catabolic process	GO:0031145	4.40E-02	ENSSSCG00000003949	*CDC20*
Gene Ontology	positive regulation of ERK1 and ERK2 cascade	GO:0070374	4.61E-02	ENSSSCG00000014007|ENSSSCG00000009565	*FLT4|GAS6*
Gene Ontology	growth factor binding	GO:0019838	4.71E-02	ENSSSCG00000014007	*FLT4*
Gene Ontology	viral entry into host cell	GO:0046718	4.71E-02	ENSSSCG00000023743	*CLDN6*
Gene Ontology	adenylate cyclase-activating adrenergic receptor signaling pathway	GO:0071880	4.71E-02	ENSSSCG00000008122	*ADRA2B*
Gene Ontology	TOR signaling	GO:0031929	4.71E-02	ENSSSCG00000002672	*MEAK7*
Gene Ontology	meiotic cell cycle	GO:0051321	4.71E-02	ENSSSCG00000029264	*PKMYT1*
Gene Ontology	lactation	GO:0007595	4.71E-02	ENSSSCG00000009261	*CSN2*
Gene Ontology	ATP binding	GO:0005524	4.76E-02	ENSSSCG00000029264|ENSSSCG00000016726|ENSSSCG00000007585|ENSSSCG00000002671|ENSSSCG00000014007|ENSSSCG00000006487|ENSSSCG00000002375	*PKMYT1|ADCY1|ACTB|ATP2C2|FLT4|CCT3|RPS6KL1*
Gene Ontology	protein kinase activity	GO:0004672	4.93E-02	ENSSSCG00000002375|ENSSSCG00000029264	*RPS6KL1|PKMYT1*

Table S7 The lncRNAs and genes involved in significant modules

Greenyellow	Gene symbol	Green	Gene symbol	Black	Gene symbol	Yellow	Gene symbol	Brown	Gene symbol	Blue	Gene symbol	Turquoise	Gene symbol
XLOC_012374*		XLOC_024971*		XLOC_016878*		XLOC_000412*		XLOC_007694*		ENSSSCG00000006581*		XLOC_023369*	
XLOC_025150*		XLOC_025062*		ENSSSCG00000002287	*MPP5*	XLOC_024728*		ENSSSCG00000002257	*MCTP2*	ENSSSCG00000016379*		XLOC_653128*	
XLOC_008121*		XLOC_025410*		ENSSSCG00000002375	*RPS6KL1*	XLOC_025049*		ENSSSCG00000002285	*GPHN*	ENSSSCG00000000220	*POU6F1*	XLOC_025340*	
XLOC_006489*		ENSSSCG00000005094*		ENSSSCG00000002671	*ATP2C2*	XLOC_025406*		ENSSSCG00000002672	*MEAK7*	ENSSSCG00000001511	*PHF1*	XLOC_004713*	
XLOC_026157*		ENSSSCG00000011196*		ENSSSCG00000003353	*SLC35E2B*	XLOC_010589*		ENSSSCG00000003189	*PRMT1*	ENSSSCG00000001641	*UBR2*	XLOC_020379*	
XLOC_408409*		ENSSSCG00000000040	*SEPTIN3*	ENSSSCG00000004438	*TSPYL1*	ENSSSCG00000000114	*PICK1*	ENSSSCG00000003701	*SNRPD1*	ENSSSCG00000001804	*HOMER2*	XLOC_001405*	
XLOC_012730*		ENSSSCG00000000171	*CKAP4*	ENSSSCG00000004577	*ICE2*	ENSSSCG00000001076	*RNF144B*	ENSSSCG00000003949	*CDC20*	ENSSSCG00000002266	NA	XLOC_570364*	
XLOC_013304*		ENSSSCG00000003344	*VWA1*	ENSSSCG00000005761	*PPP1R26*	ENSSSCG00000001646	*BICRAL*	ENSSSCG00000003951	*C1orf210*	ENSSSCG00000002392	NA	ENSSSCG00000007311*	
XLOC_005330*		ENSSSCG00000003410	*MASP2*	ENSSSCG00000006098	*DPY19L4*	ENSSSCG00000001659	*KLC4*	ENSSSCG00000004294	*SYNCRIP*	ENSSSCG00000002627	*GSTA4*	ENSSSCG00000013753*	
XLOC_015988*		ENSSSCG00000004420	*TRAF3IP2*	ENSSSCG00000006572	*NPR1*	ENSSSCG00000002429	*FOXN3*	ENSSSCG00000004508	*DYM*	ENSSSCG00000002937	NA	ENSSSCG00000000034	*TTLL12*
XLOC_004742*		ENSSSCG00000005707	*FIBCD1*	ENSSSCG00000007027	*SLC20A2*	ENSSSCG00000002628	*CILK1*	ENSSSCG00000005908	*SLC52A2*	ENSSSCG00000002955	*CATSPERG*	ENSSSCG00000000223	*BIN2*
ENSSSCG00000040582*		ENSSSCG00000006651	*ADAMTSL4*	ENSSSCG00000007312	*SCAND1*	ENSSSCG00000002629	*FBXO9*	ENSSSCG00000005909	*FBXL6*	ENSSSCG00000002965	*ACTN4*	ENSSSCG00000000521	*PHLDA1*
ENSSSCG00000006206	NA	ENSSSCG00000007239	*CCM2L*	ENSSSCG00000007826	NA	ENSSSCG00000002959	*FAM98C*	ENSSSCG00000006487	*CCT3*	ENSSSCG00000003192	*IL4I1*	ENSSSCG00000000522	*NAP1L1*
ENSSSCG00000009702	*FBXO8*	ENSSSCG00000007497	*GCNT7*	ENSSSCG00000007896	*TXNDC11*	ENSSSCG00000003199	*AP2A1*	ENSSSCG00000006939	*ZNHIT6*	ENSSSCG00000003342	*DVL1*	ENSSSCG00000001419	*SLC44A4*
ENSSSCG00000011125	*GATA3*	ENSSSCG00000007542	*PRKAR1B*	ENSSSCG00000009267	*CSN3*	ENSSSCG00000003278	NA	ENSSSCG00000007031	*POLB*	ENSSSCG00000003755	*MCOLN2*	ENSSSCG00000001420	*EHMT2*
ENSSSCG00000012479	*PCDH19*	ENSSSCG00000007812	*XPO6*	ENSSSCG00000011470	*ABHD6*	ENSSSCG00000003577	*WASF2*	ENSSSCG00000007064	*MKKS*	ENSSSCG00000003995	*ZNF606*	ENSSSCG00000001421	*ZBTB12*
ENSSSCG00000012741	*MAMLD1*	ENSSSCG00000009162	*SLC9B1*	ENSSSCG00000011850	*MUC4*	ENSSSCG00000004002	*RPS5*	ENSSSCG00000007362	*SRSF6*	ENSSSCG00000004223	*HEY2*	ENSSSCG00000001422	*C2*
ENSSSCG00000038508	*SPTBN2*	ENSSSCG00000009228	*MAPK10*	ENSSSCG00000012532	*TCEAL1*	ENSSSCG00000005383	*ALG2*	ENSSSCG00000007494	*CSTF1*	ENSSSCG00000004772	*BAHD1*	ENSSSCG00000001657	*CUL7*
		ENSSSCG00000009664	*PTK2B*	ENSSSCG00000012613	*NKAP*	ENSSSCG00000005582	*STRBP*	ENSSSCG00000007669	*GNB2*	ENSSSCG00000005015	*SOS2*	ENSSSCG00000001658	*MRPL2*
		ENSSSCG00000009665	*CHRNA2*	ENSSSCG00000013282	*ACCS*	ENSSSCG00000005904	*VPS28*	ENSSSCG00000007708	*BCL7B*	ENSSSCG00000005917	*HSF1*	ENSSSCG00000001660	*PTK7*
		ENSSSCG00000009705	*GALNT7*	ENSSSCG00000016223	*ACSL3*	ENSSSCG00000006166	*NDUFB8*	ENSSSCG00000007713	*BUD23*	ENSSSCG00000005920	NA	ENSSSCG00000002440	*CCDC88C*
		ENSSSCG00000010370	*ANXA8*	ENSSSCG00000016498	*MKRN1*	ENSSSCG00000006350	*FCGR2B*	ENSSSCG00000007715	*ABHD11*	ENSSSCG00000006490	*SMG5*	ENSSSCG00000002708	*TMEM231*
		ENSSSCG00000011195	*GALNT15*	ENSSSCG00000016549	*MKLN1*	ENSSSCG00000006362	*USP21*	ENSSSCG00000007721	*GTF2I*	ENSSSCG00000006582	*S100A14*	ENSSSCG00000002953	NA

ENSSSCG00000012845	*CEND1*	ENSSSCG00000016564	*UBE2H*	ENSSSCG00000006954	*EEF1D*	ENSSSCG00000007803	*TUFM*	ENSSSCG00000006648	*CTSS*	ENSSSCG00000003194	*AKT1S1*
ENSSSCG00000012853	*IRF7*			ENSSSCG00000007155	*C20orf194*	ENSSSCG00000008675	*LETM1*	ENSSSCG00000006665	*SF3B4*	ENSSSCG00000003329	*ACAP3*
ENSSSCG00000012984	*SCYL1*			ENSSSCG00000007228	*HM13*	ENSSSCG00000008682	*NSD2*	ENSSSCG00000007026	*VDAC3*	ENSSSCG00000003578	*FGR*
ENSSSCG00000014805	NA			ENSSSCG00000007252	*DNMT3B*	ENSSSCG00000008788	*RFC1*	ENSSSCG00000007030	*IKBKB*	ENSSSCG00000003773	*AK5*
ENSSSCG00000014843	*CHRDL2*			ENSSSCG00000007806	*RABEP2*	ENSSSCG00000009119	*LARP7*	ENSSSCG00000007206	*RBCK1*	ENSSSCG00000004222	*NCOA7*
ENSSSCG00000016381	*SNED1*			ENSSSCG00000008122	*ADRA2B*	ENSSSCG00000009120	*ZGRF1*	ENSSSCG00000007249	*NOL4L*	ENSSSCG00000004275	*FAM135A*
ENSSSCG00000016409	*UBE3C*			ENSSSCG00000008335	NA	ENSSSCG00000009233	*GPAT3*	ENSSSCG00000007309	*RBM39*	ENSSSCG00000004279	*OGFRL1*
ENSSSCG00000016430	*GALNT11*			ENSSSCG00000008959	*CXCL2*	ENSSSCG00000009560	*TFDP1*	ENSSSCG00000007364	*IFT52*	ENSSSCG00000004293	*SNX14*
ENSSSCG00000016719	*STK31*			ENSSSCG00000008961	*MTHFD2L*	ENSSSCG00000009653	*CDCA2*	ENSSSCG00000007602	*BAIAP2L1*	ENSSSCG00000004435	*NT5DC1*
ENSSSCG00000016976	*ZNF366*			ENSSSCG00000008973	NA	ENSSSCG00000010143	*MTR*	ENSSSCG00000007720	*GTF2IRD1*	ENSSSCG00000004657	*CEP152*
ENSSSCG00000017783	NA			ENSSSCG00000009390	*SPRYD7*	ENSSSCG00000010801	*CDC73*	ENSSSCG00000007804	*SH2B1*	ENSSSCG00000004771	NA
ENSSSCG00000020799	*CPSF3*			ENSSSCG00000012528	*BEX3*	ENSSSCG00000012879	NA	ENSSSCG00000008336	*ANXA4*	ENSSSCG00000004979	*MYO9A*
				ENSSSCG00000012689	*ZNF75D*	ENSSSCG00000013028	*ESRRA*	ENSSSCG00000008681	*NELFA*	ENSSSCG00000004985	*GEMIN2*
				ENSSSCG00000012842	*RPLP2*	ENSSSCG00000013276	*PRDM11*	ENSSSCG00000008947	*COX18*	ENSSSCG00000005022	*NIN*
				ENSSSCG00000012850	*DEAF1*	ENSSSCG00000013468	*THOP1*	ENSSSCG00000009239	NA	ENSSSCG00000005150	*KLHL9*
				ENSSSCG00000012854	*RASSF7*	ENSSSCG00000013478	*DOHH*	ENSSSCG00000009242	*LIN54*	ENSSSCG00000005191	*MPDZ*
				ENSSSCG00000012979	*RNASEH2C*	ENSSSCG00000014003	*MIER2*	ENSSSCG00000009243	*THAP9*	ENSSSCG00000005759	*MRPS2*
				ENSSSCG00000013597	*RPS28*	ENSSSCG00000014436	*ARHGEF37*	ENSSSCG00000009245	*SCD5*	ENSSSCG00000005828	*EGFL7*
				ENSSSCG00000013742	*NFIX*	ENSSSCG00000014893	*NARS2*	ENSSSCG00000009261	*CSN2*	ENSSSCG00000005830	*CCDC183*
				ENSSSCG00000014061	*THOC3*	ENSSSCG00000015336	*SLC25A13*	ENSSSCG00000009262	*CSN1S1*	ENSSSCG00000005907	*ADCK5*
				ENSSSCG00000014062	*SIMC1*	ENSSSCG00000015384	*TOMM7*	ENSSSCG00000009567	*RASA3*	ENSSSCG00000005910	*TMEM249*
				ENSSSCG00000014071	*BTF3*	ENSSSCG00000015524	*FAM20B*	ENSSSCG00000009925	*GIT2*	ENSSSCG00000006301	*TIPRL*
				ENSSSCG00000014149	*MEF2C*	ENSSSCG00000015710	*ACTR3*	ENSSSCG00000010179	*ARV1*	ENSSSCG00000006307	*RCSD1*
				ENSSSCG00000014957	*C11orf97*	ENSSSCG00000016232	*MRPL44*	ENSSSCG00000010181	*C1orf198*	ENSSSCG00000006347	*DUSP12*
				ENSSSCG00000014959	*PIWIL4*	ENSSSCG00000016378	*PASK*	ENSSSCG00000011101	*ITGB1*	ENSSSCG00000006612	*S100A10*
				ENSSSCG00000015271	*PRELP*	ENSSSCG00000016522	*PTN*	ENSSSCG00000011332	*SETD2*	ENSSSCG00000006956	*ZC3H3*

ENSSSCG00000015405	*CD36*	ENSSSCG00000016738	*H2AZ2*	ENSSSCG00000012519	*GPRASP1*	ENSSSCG00000007162	*UBOX5*
ENSSSCG00000016431	*GALNTL5*	ENSSSCG00000016756	*BLVRA*	ENSSSCG00000012520	NA	ENSSSCG00000007212	*C20orf96*
ENSSSCG00000016726	*ADCY1*	ENSSSCG00000017126	*NARF*	ENSSSCG00000012586	NA	ENSSSCG00000007585	*ACTB*
ENSSSCG00000016753	*POLD2*	ENSSSCG00000017626	*SRSF1*	ENSSSCG00000012617	*UPF3B*	ENSSSCG00000007664	*AGFG2*
ENSSSCG00000016755	*POLM*	ENSSSCG00000020665	*TAF6L*	ENSSSCG00000012695	*INTS6L*	ENSSSCG00000007717	*METTL27*
				ENSSSCG00000012767	*HAUS7*	ENSSSCG00000007808	*NFATC2IP*
				ENSSSCG00000012811	*VBP1*	ENSSSCG00000007967	*ZNF213*
				ENSSSCG00000012848	*EPS8L2*	ENSSSCG00000007985	*PGAP6*
				ENSSSCG00000012875	*TPCN2*	ENSSSCG00000008578	*ADCY3*
				ENSSSCG00000012956	*PACS1*	ENSSSCG00000009234	*ABRAXAS1*
				ENSSSCG00000013467	*SGTA*	ENSSSCG00000009236	*HELQ*
				ENSSSCG00000013602	*HNRNPM*	ENSSSCG00000009563	*TMCO3*
				ENSSSCG00000014443	*CAMK2A*	ENSSSCG00000009565	*GAS6*
				ENSSSCG00000015644	*IKBKE*	ENSSSCG00000009683	*KIF13B*
				ENSSSCG00000016370	*SEPTIN2*	ENSSSCG00000010180	*TTC13*
				ENSSSCG00000016432	*PRKAG2*	ENSSSCG00000010621	*ADD3*
				ENSSSCG00000016569	*SMO*	ENSSSCG00000010640	*NRAP*
				ENSSSCG00000016679	NA	ENSSSCG00000010780	*CYP2E1*
				ENSSSCG00000017128	*HEXD*	ENSSSCG00000011075	*KIAA1217*
				ENSSSCG00000017137	*METRNL*	ENSSSCG00000011085	*MLLT10*
						ENSSSCG00000011107	*CCNY*
						ENSSSCG00000011179	*MRPL3*
						ENSSSCG00000012843	*PIDD1*
						ENSSSCG00000013463	*LSM7*
						ENSSSCG00000013715	NA
						ENSSSCG00000013744	*TRMT1*

ENSSSCG00000013839	*RASAL3*
ENSSSCG00000014007	*FLT4*
ENSSSCG00000014539	*ZFP62*
ENSSSCG00000014802	*NUMA1*
ENSSSCG00000015267	*FMO2*
ENSSSCG00000015270	*FMOD*
ENSSSCG00000015780	*STOX2*
ENSSSCG00000015814	NA
ENSSSCG00000016368	*FARP2*
ENSSSCG00000016572	*TNPO3*
ENSSSCG00000016720	*PGAM2*
ENSSSCG00000016721	*DBNL*
ENSSSCG00000016754	*AEBP1*
ENSSSCG00000016763	*GLI3*
ENSSSCG00000016894	*ARL15*
ENSSSCG00000016968	*BDP1*
ENSSSCG00000017133	*RAB40B*
ENSSSCG00000017136	*TBCD*
ENSSSCG00000017379	*ETV4*
ENSSSCG00000020879	*FBXW7*
ENSSSCG00000021220	*CKB*
ENSSSCG00000022129	*ARSL*
ENSSSCG00000023933	*CRACR2B*
ENSSSCG00000029600	NA
ENSSSCG00000037035	*UVSSA*

Note: * indicates lncRNAs. NA indicates novel gene.
